# Immunoinformatics Approach to Design a Multi-Epitope Nanovaccine against *Leishmania* Parasite: Elicitation of Cellular Immune Responses

**DOI:** 10.3390/vaccines11020304

**Published:** 2023-01-30

**Authors:** Maritsa Margaroni, Maria Agallou, Evgenia Tsanaktsidou, Olga Kammona, Costas Kiparissides, Evdokia Karagouni

**Affiliations:** 1 Immunology of Infection Laboratory, Hellenic Pasteur Institute, 125 21 Athens, Greece; 2 Chemical Process & Energy Resources Institute, Centre for Research and Technology Hellas, 57 001 Thessaloniki, Greece; 3 Department of Chemical Engineering, Aristotle University of Thessaloniki, 54 124 Thessaloniki, Greece

**Keywords:** leishmaniasis, reverse vaccinology, MHCI-binding epitopes, MHCII-binding epitopes, multi-epitope vaccine, cellular immunity, memory T cells, cytokine production

## Abstract

Leishmaniasis is a vector-borne disease caused by an intracellular parasite of the genus *Leishmania* with different clinical manifestations that affect millions of people worldwide, while the visceral form may be fatal if left untreated. Since the available chemotherapeutic agents are not satisfactory, vaccination emerges as the most promising strategy for confronting leishmaniasis. In the present study, a reverse vaccinology approach was adopted to design a pipeline starting from proteome analysis of three different *Leishmania* species and ending with the selection of a pool of MHCI- and MHCII-binding epitopes. Epitopes from five parasite proteins were retrieved and fused to construct a multi-epitope chimeric protein, named LeishChim. Immunoinformatics analyses indicated that LeishChim was a stable, non-allergenic and immunogenic protein that could bind strongly onto MHCI and MHCII molecules, suggesting it as a potentially safe and effective vaccine candidate. Preclinical evaluation validated the in silico prediction, since the LeishChim protein, encapsulated simultaneously with monophosphoryl lipid A (MPLA) into poly(D,L-lactide-co-glycolide) (PLGA) nanoparticles, elicited specific cellular immune responses when administered to BALB/c mice. These were characterized by the development of memory CD4^+^ T cells, as well as IFNγ- and TNFα-producing CD4^+^ and CD8^+^ T cells, supporting the potential of LeishChim as a vaccine candidate.

## 1. Introduction

Leishmaniasis is a group of diseases that are caused by intracellular parasites of the genus *Leishmania* and display a spectrum of clinical manifestations from self-healing cutaneous to life-threatening visceral forms of disease [1]. The diseases are prevalent in large areas of tropical, subtropical and Mediterranean countries. According to the WHO, 310 million people are at risk; nearly 90,000 new cases of visceral leishmaniasis annually occur, with 30,000 deaths per year [2]. *Leishmania* is an obligate intracellular parasite with two different life forms: amastigote and promastigote. More than 20 species are responsible for the four common manifestations of the disease which accounts for cutaneous leishmaniasis (CL), mucocutaneous leishmaniasis (MCL), diffuse cutaneous leishmaniasis (DCL) and visceral leishmaniasis (VL) [3,4]. The first line of confronting the disease includes drug treatment; however, the currently available drugs are characterized by high toxicity, parasite resistance and high cost [5,6,7]. In that frame, alternative control measures such as the development of effective vaccines have been suggested, aiming to prevent the spread of leishmaniasis. Vaccination has been proposed as the most effective strategy for controlling infectious diseases, since it can induce long-term protection with appropriate immune responses [8]. Several vaccination strategies, including killed or attenuated parasites, subunit or DNA vaccines, have been developed [9,10]; however, no vaccine for humans has entered the market until now.

The elicitation of T cell-mediated immune responses is considered critical for *Leishmania* infection control. In particular, the interaction of antigen-presenting cells with CD4^+^ and/or CD8^+^ T cells mediate T helper 1 (T_H_1) responses through the secretion of pro-inflammatory cytokines, such as IFNγ and TNFα, which in turn enhance the activation of macrophages and the subsequent parasite control. The efficiency of CD4^+^ T_H_1 and cytotoxic CD8^+^ T cell responses to mediate resistance or cure in VL depends on their memory phenotype. On the contrary, T_H_2 responses associated with an anti-inflammatory cytokine profile inhibit macrophage activation favoring disease establishment (reviewed in [11,12]).

Based on the fact that the elicitation of T_H_1 CD4^+^ and cytotoxic CD8^+^ T cells has been associated with effective vaccination against experimental leishmaniasis [13,14], vaccine formulations consisting of different antigenic helper and cytotoxic T lymphocyte epitopes (HTL and CTL) and adjuvants have been tested by several research groups [15]. The advance in immunoinformatics approaches has contributed to the identification of specific HTL and CTL epitopes that could be included in multi-epitope vaccines [16]. This vaccination strategy, contrary to previous efforts including killed or attenuated parasites, appears to have several advantages such as specificity, stability, absence of infectious agents and large-scale production at low cost [15].

Based on the above knowledge regarding vaccine development, we conducted a subtractive hierarchical analysis on the *Leishmania* proteome with the ultimate goal of designing a universal multi-epitope chimeric protein vaccine. Our analysis resulted in the identification of five antigenic parasite proteins, from which their most promising epitopes were selected and fused into a chimeric protein, namely LeishChim. The physicochemical properties, the secondary and tertiary structure, as well as the immune profile of this protein, were predicted using bioinformatics tools. Subsequently, LeishChim’s antigenicity was also assessed in vivo. For this purpose, protein was co-encapsulated with monophosphoryl lipid A (MPLA), a known TLR-4 ligand, into biodegradable poly(D,L-lactide-co-glycolide) (PLGA) nanoparticles (NPs) in order to enhance the delivery as well as its immunogenic properties. PLGA NPs possess several advantages, such as protection from degradation by enzymes, controlled antigen release, as well as adjuvant co-encapsulation [17,18], making them ideal candidates as delivery vehicles for tumor [19], viral [20] or parasitic antigens [21,22,23]. Eventually, the immune profile elicited by the nanovaccine formulation was further evaluated in an experimental BALB/c model.

## 2. Materials and Methods

### 2.1. Data Retrieval and Proteome Analysis

The proteome of the *L. infantum*, *L. major* and *L. braziliensis* species was retrieved as FASTA format from TriTrypDB database (http//:tritrypdb.org, accessed on 23 November 2018). BLASTp tool was used to identify proteins, having >80% conservancy among the three studied species (https://blast.ncbi.nlm.nih.gov/Blast.cgi?PAGE=Proteins, accessed on 5 December 2018). Followingly, cellular localization of the retrieved proteins was identified with CELLO. CELLO (http://cello.life.nctu.edu.tw, accessed on 6 December 2018) is a multi-class SVM classification system that uses 4 types of sequence coding schemes: the amino acid composition, the dipeptide composition, the partitioned amino acid composition and the sequence composition based on the physicochemical properties of amino acids. It combines votes from these classifiers and uses the jury votes to determine the final assignment. The accuracy for prediction of CELLO algorithm is 87% for eukaryotic sequences [24]. In parallel, SignalP v5.0 (http://www.cbs.dtu.dk/services/SignalP/, accessed on 15 December 2018) algorithm was used to predict secreted proteins. The SignalP 5.0 server predicts the presence of signal peptides and the location of their cleavage sites in proteins from archaea, Gram-positive bacteria, Gram-negative bacteria and eukarya. Version 5.0 uses a deep neural network-based approach that improves signal peptide prediction across all domains of life and distinguishes between three types of prokaryotic signal peptides [25].

### 2.2. Antigenicity and Allergenicity Evaluation

The antigenicity of the retrieved proteins was predicted via employment of AntigenPro and VaxiJen. AntigenPro (http://scratch.proteomics.ics.uci.edu/, accessed on 5 December 2018) is a homologue-free, sequence-based prediction model capable of screening entire proteomes for the antigens most likely to generate a protective humoral immune response with an accuracy of 82% [26]. Threshold was set to 0.4. VaxiJen v2.0 (http://www.ddg-pharmfac.net/vaxijen/VaxiJen/VaxiJen.html, accessed on 15 January 2019) is an alignment-independent method based on auto-cross covariance (ACC) transformation of protein sequences into uniform vectors of principal amino acid properties. The accuracy of the method depends on organism selection and varies from 70% to 89% [27]. For the needs of this study, the parasite was selected as the target organism and the threshold was set to 0.5. Proteins with antigenicity values above threshold in both algorithms were further evaluated for the probability to cause allergic reactions. The allergenicity of leishmanial proteins was evaluated by three different algorithms: AllerTop v.2.0, AlgPred and AllergenFP v.1.0. AllerTop v.2.0 server (http://www.ddg-pharmfac.net/AllerTOP/index.html, accessed on 20 January 2019) is an available online server based on ACC transformation of protein sequences into uniform equal-length vectors. Using k-nearest neighbors (kNN) algorithm, it predicts allergens and non-allergens with 85.3% accuracy [28]. The AlgPred, available at https://webs.iiitd.edu.in/raghava/algpred/submission.html (accessed on 20 January 2019, utilizes a dataset of 578 allergens and 700 non-allergens. AlgPred is an SVM (support vector machine)-based method that predicts allergens with an accuracy of 85% [29]. AllergenFP (https://ddg-pharmfac.net/AllergenFP/, accessed on 21 January 2019) is an alignment-free server that distinguishes between allergens and non-allergens by their structural and physicochemical properties. The algorithm’s accuracy is 87% for allergens and 89% for non-allergens [30]. Proteins predicted as antigenic and non-allergens by all three algorithms were then subjected to epitope mapping analysis.

### 2.3. T Cell Epitope Prediction, Epitope Selection and Vaccine Construction

Three different algorithms were used for the prediction of epitopes of antigenic and non-allergenic proteins recognized from human MHC class I haplotypes: IEDB, NetMHCpan and SYFPEITHI. IEDB is an online server (http://tools.iedb.org/mhci/, accessed on 5 February 2019) that predicts ΜHC-binding epitopes by different prediction methods. In this study, the IEDB recommended method was used, while HLA allele reference set was chosen. Cut-off score was adjusted to <1.0 and peptides recognized from more than three HLA alleles were finally qualified [31]. Regarding the NetMHCpan algorithm (http://www.cbs.dtu.dk/services/NetMHCpan/, accessed on 7 February 2019), epitopes with binding force to the histocompatibility molecules <50 nM (Strong Binder, SB) or <500 nM (Weak Binder, WB), respectively, were selected [32]. For SYFPEITHI server, available at http://www.syfpeithi.de/bin/MHCServer.dll/EpitopePrediction.htm (accessed on 8 February 2019, the acceptance limits were set >20 [33].

The prediction of MHCII-binding epitopes was also performed with three servers: IEDB, NETMHCIIpan and SYFPEITHI. IEDB (http://tools.iedb.org/mhcii/, accessed on 10 February 2019) was employed to predict 15-mer-length epitopes. This online resource contains an extensive collection of experimentally measured immune epitopes. Antigenic proteins were submitted to IEDB server as 15 mers overlapping by ten residues. In order to split the proteins, an online server was used (www.bioinformatics.nl/cgi-bin/emboss/splitter, accessed on 10 February 2019). A percentile rank cut-off <20 was employed and peptides binding to more than 50%, i.e., 13, of the full HLA reference set alleles, were finally selected [31]. The NETMHCIIpan 3.2 server is an online tool (http://www.cbs.dtu.dk/services/NetMHCIIpan/, accessed on 15 February 2019) constructed using an extended dataset of quantitative MHC–peptide binding affinity data [34]. A set of 20 DQ, DP and DR alleles was used, common with those used in IEDB reference set. Threshold was set as <10 and peptides binding more than 10 alleles were finally selected. The third online tool for MHCII-binding epitopes’ prediction was SYFPEITHI (http://www.syfpeithi.de/bin/MHCServer.dll/EpitopePrediction.htm, accessed on 10 February 2019). Cut-off was set >20 and peptides binding to more than three out of the six available alleles were qualified for further study. 

The above-mentioned tools were also used for the prediction of epitopes recognized by BALB/c MHCI (H2-Dd, H2-Kd and H2-Ld) and MHCII (H2-IAd and H2-IEd) molecules aiming to identify epitopes commonly recognized by human and mouse.

The set of epitopes predicted from the above analysis were run in BLASTp tool in order to exclude those having >45% homology to human or murine genome in order to avoid autoimmune or allergic reactions. Next, proteins including >5 MHCI and/or MHCII epitopes that covered >20% of their sequence and, moreover, showed <45% of homology to human or murine sequence, were forwarded for chimeric protein design. Subsequently, sequences including neighboring or overlapping epitopes from proteins LinJ.34.0420 (Hypothetical protein), LinJ.18.0650 (Serine/threonine kinase-like protein), LinJ.28.2200 (DNA-directed RNA polymerase-like protein), LinJ.28.0780 (Hypothetical protein) and LinJ.20.0600 (Conserved unknown protein) were assembled in tandem to construct a chimeric protein named LeishChim.

### 2.4. Immunoinformatics Analysis of Chimeric Protein

#### 2.4.1. Physicochemical Properties

The physicochemical properties of LeishChim were determined using the Protparam server (https://web.expasy.org/protparam/, accessed on 11 March 2019) [35]. The predicted physicochemical parameters included molecular weight (MW), instability index, grand average of hydropathicity (GRAVY), aliphatic index and theoretical pI.

#### 2.4.2. Secondary and Tertiary Structure

The secondary structure of the chimeric protein was predicted using PSIPRED algorithm [36], whereas protein’s tertiary structure was predicted via i-TASSER server (https://zhanglab.dcmb.med.umich.edu/I-TASSER/, accessed on 11 March 2019). i-TASSER identifies structural templates from the PDB by the multiple-threading approach LOMETS, with full-length atomic models constructed by iterative template-based fragment assembly simulations [37]. The quality of the model predicted by i-TASSER was estimated by a confidence score (C-score). A model with higher C-score is of higher confidence. The visualization of the 3D model was conducted using PyMOL molecular visualization system. 

#### 2.4.3. Refinement and Validation of Tertiary Structure Model

The tertiary structure of LeishChim was refined with GalaxyRefine server (http://galaxy.seoklab.org/cgi-bin/submit.cgi?type=REFINE, accessed on 11 March 2019). This server relies on a method that first rebuilds side chains and performs side-chain repacking and subsequent overall structure relaxation by molecular dynamics simulation [38]. GalaxyRefine improves the initial models with a probability > 50% [39]. In order to validate the 3D model and detect potential errors in the predicted model, ProSA-web server was applied, while its correctness was determined by ERRAT and Ramachandran plots. ProSA-web (https://prosa.services.came.sbg.ac.at/prosa.php, accessed on 11 March 2019) calculates an overall quality score for a specific input structure. The server requires only Ca atoms so that low resolution structures can be evaluated. ProSA-web calculates the interaction energy of each residue with the rest of the structure and shows an error in the protein structure if the calculated score is out of the protein native range [40]. The ERRAT server (https://servicesn.mbi.ucla.edu/ERRAT/, accessed on 12 March 2019) differentiates between correctly and incorrectly determined regions of the protein structures based on characteristic atomic interaction [41]. Ramachandran plots were assessed with PROCHEK (https://www.ebi.ac.uk/thornton-srv/software/PROCHECK/index.html, accessed on 12 March 2019), which checks the stereochemical quality of a protein structure, producing a number of PostScript plots analyzing its overall and residue-by-residue geometry [42].

#### 2.4.4. In Silico Prediction of LeishChim’s Docking to MHC Class I and II Molecules

The prediction of the chimeric protein’s ability to dock to different MHCI and MHCII molecules was conducted with PatchDock server (https://bioinfo3d.cs.tau.ac.il/PatchDock/patchdock.html, accessed on 12 March 2019). PatchDock is inspired by object recognition and image segmentation techniques used in Computer Vision. The algorithm has three main steps: molecular shape representation, surface patch matching and filtering and scoring [43]. Docking was predicted against HLA-A*0201, HLA-A*0101, HLA-B*0702, HLA-B*3501, HLA-DRB1*03:01, HLA-DRB5*01:01, HLA-DRB1*01:01, HLA-DRB3*02:02. FireDock server was utilized for the refinement of the docking model. FireDock also refines scores solutions produced by fast rigid-body docking algorithms according to an energy function [44]. 

#### 2.4.5. Molecular Dynamics (MD) Simulation of LeishChim-MHCI/MHCII Complexes

The prediction of the stability of LeishChim protein’s binding to MHCI and MHCII molecules was performed with MD simulation using iMODS webserver (http://imods.chaconlab.rog, accessed on 17 January 2023). This particular server was employed due to the advantage of being efficient and faster compared to other processes of MD simulations [45]. iMODS server creates protein models as a set of atoms connected by harmonic springs and uses normal mode analysis (NMA) to analyze the collective motion of proteins [46]. Deformability, B-factors, eigenvalues, mode variance plot, covariance map and elastic network are provided to assess the flexibility and stiffness of the complex, as well as the correlated, uncorrelated and anti-correlated motions between dynamical regions [47].

#### 2.4.6. CTL, HTL, B Cell Linear and Conformational Epitope Prediction

NetCTL 1.2 server (http://www.cbs.dtu.dk/services/NetCTL/, accessed on 25 August 2020) was utilized to predict the CTL epitopes in LeishChim. Prediction involves a combination of three approaches, MHCI binding affinity, TAP transport efficiency and proteasomal C-terminal cleavage. CTL epitope prediction was restricted by 12 MHCI supertypes [48]. MHCI peptide binding epitope prediction was attained by the use of artificial neural networks, TAP transport efficiency was achieved by an approach based on weight matrix [49], while C-terminal proteasomal cleavage site was predicted by the artificial neural network from NetChop 3.1 server (https://services.healthtech.dtu.dk/service.php?NetChop-3.1, accessed on 25 August 2020). Scores of all three predictions were merged together and sensitivity or specificity values can be achieved by the conversion of the threshold from the merged score. Threshold for epitope identification was set at 0.75. The prediction of MHCII-binding epitopes was performed as described above (Section 2.3).

Furthermore, ElliPro was used to predict linear and conformational B cell epitopes in chimeric protein. This server predicts antibody epitopes based on a protein antigen’s 3D structure [50]. Cut-off was set at 0.5.

#### 2.4.7. Immune Simulations

In silico immune simulations were performed using C-ImmSim online server (https://kraken.iac.rm.cnr.it/C-IMMSIM/, accessed on 25 November 2020) to predict the immune profile raised by LeishChim. The C-ImmSim utilizes the Celada–Seiden model for describing both humoral and cellular immune profiles of mammalian immune system responding to designed vaccines [51]. Simulation was run using 2 injections at a time step of 45, while vaccine was administered with no LPS. All other parameters were as by default, i.e., random seed 12345, simulation volume 10, simulation steps 100. As host molecules, MHCI A0101 and 0201, B0702 and 0735 as well as MHCII DRB1 0101 and 0301 were chosen.

### 2.5. Expression of Chimeric Protein

The chimeric multi-epitope protein LeishChim was submitted for codon optimization and was commercially synthesized by GeneCust (Boynes, France). Briefly, the recombinant protein was expressed in *Escherichia coli* BL21 (DE3) and the pET-22b(+) vector was used to insert the optimized sequence. The expression was induced with 0.5 mM IPTG for 3 h at 37 °C. Then, the protein was purified in Ni-NTA column and analyzed by SDS-PAGE. The endotoxin was removed by the endotoxin removal beads and the endotoxin level was less than 1 EU/μg.

### 2.6. PLGA Vaccine Preparation and Characterization

#### 2.6.1. Materials

Poly(lactide-co-glycolide) (PLGA) (Resomer RG752H, MW: 4–15 kDa), polyvinyl alcohol (PVA) (MW: 30–70 kDa, 87–90% hydrolyzed), monophosphoryl lipid A (MPLA), phosphate-buffered saline (PBS, 10x, pH 7.4) were purchased from Sigma. All other reagents were of analytical grade and commercially available. 

#### 2.6.2. Preparation and Characterization of PLGA Nanoparticles

PLGA nanoparticles (NPs) containing the chimeric protein LeishChim and the adjuvant monophosphoryl lipid A (MPLA) were prepared by the double emulsion method. Initially, 2.9 mL of a PLGA solution in chloroform (31 mg/mL) was mixed with 0.1 mL of an MPLA solution (10 mg/mL) in methanol:chloroform (1:4 *v*/*v*). A water-in-oil (w/o) emulsion was subsequently formed by adding 0.3 mL of LeishChim solution in urea 8M (5 mg/mL) into that of PLGA/MPLA. The emulsification was performed in an ice bath with the aid of a microtip sonicator (Sonicator Sonics Vibra Cell VC-505) at 40% amplitude for 45 s. Subsequently, the w/o emulsion was added into an aqueous PVA solution (12 mL, 1 w/v %) and the mixture was emulsified via sonication at 40% amplitude for 2 min. The resulting double (w/o/w) emulsion was stirred overnight to allow the evaporation of chloroform. The PLGA NPs were then purified by means of four successive centrifugation–redispersion cycles (in HPLC water, at 20,000 rpm and 4 °C for 10 min) and were subsequently lyophilized (CoolSafe 55-4, Labogene). Blank, MPLA-loaded and LeishChim-loaded PLGA NPs were also prepared and used as controls. For the preparation of LeishChim-loaded PLGA NPs, the protein solution (0.3 mL, 5 mg/mL) was added into 3.0 mL of a PLGA chloroform solution (30 mg/mL). Finally, for the synthesis of the MPLA-loaded and the blank NPs, the protein solution was replaced with 0.3 mL of PBS. 

The average particle diameter of the PLGA NPs was determined by photon correlation spectroscopy and their zeta potential via aqueous electrophoresis measurements (Malvern Nano ZS90, Worcestershire, UK). All measurements were performed with aqueous dispersions of PLGA NPs prior to their lyophilization.

#### 2.6.3. Quantification of Antigen Loading

A micro-bicinchoninic acid (micro BCA) protein assay kit (Pierce Biotechnology, Rockford, IL, USA) was used to quantify LeishChim loading (wt%) in the PLGA NPs. In brief, 2.5 mg of lyophilized NPs were initially dissolved in 0.25 mL of DMSO for 1 h and further dissolved in 1.25 mL of 0.05N NaOH/0.5% SDS for 3 h at 25 °C. LeishChim concentration was determined using a micro BCA protein assay kit according to the manufacturer’s instructions for 96-microwell plates (Corning Inc., Corning, NY, USA). The absorbance of the NP solutions was measured at 562 nm using a microplate reader (EL808IU-PC, BioTek Instruments, Inc., Winooski, VT, USA). Blank PLGA NPs were also used as controls. The antigen encapsulation efficiency (%) was calculated by the ratio of LeishChim mass in the NPs over the LeishChim mass in the recipe. Similarly, LeishChim loading was calculated by the ratio of the encapsulated mass of the protein over the total mass of NPs. 

#### 2.6.4. Quantification of MPLA Loading

A Limulus Amebocyte Lysate (LAL) kit (Kinetic-QCL 192 test kit, 50-650U, LONZA) was used for quantifying MPLA loading (wt%) in the PLGA NPs. More specifically, the mass of MPLA in the nanoparticles was determined by subtracting the measured quantity of MPLA in the supernatants (collected after the washing cycles of the PLGA NPs and diluted in LAL reagent water) from the initial quantity of MPLA in the recipe. MPLA encapsulation efficiency was equal to the ratio of the measured MPLA mass in the PLGA NPs over the mass of MPLA in the recipe. Likewise, MPLA loading was equal to the ratio of encapsulated MPLA mass over the total mass of NPs.

#### 2.6.5. In Vitro Release Studies

The in vitro release of LeishChim and MPLA from PLGA NPs was performed in PBS at 37 °C. A number of vials containing 0.5 mg of NPs dispersed in 0.5 mL of PBS were incubated in a thermomixer (Thermomixer Compact, Eppendorf) at 37 °C and 1400 rpm. At specific time points (e.g., 0, 1, 2, 4, 6, 8, 12, 24, 48 h, 1, 2, 3, 4 weeks) 0.5 mL of the NPs dispersion was collected following centrifugation at 15,000 rpm and 4 °C for 10 min. The amounts of LeishChim and MPLA in the collected supernatants were quantified by the micro-BCA and LAL kits, respectively. 

#### 2.6.6. In Vitro Stability

The storage stability of the PLGA NPs containing LeishChim and MPLA was examined in PBS (pH 7.4) at 4 °C. Several vials containing 1 mg of PLGA NPs dispersed in 1 mL of PBS were incubated at 4 °C and the particle size distribution (PSD) was measured at various time points (i.e., 0, 2 and 4 weeks).

### 2.7. Experimental Animals

For our studies, female BALB/c mice (6 to 8 weeks old) were used. Mice were housed under specific pathogen-free environmental (SPF) conditions in Hellenic Pasteur Institute and were provided with sterile food and water ad libitum. All procedures complied to European Directive 2010/63/EU and PD 86/2020-A’ 199, ethical use and welfare of laboratory animals based on 3+1R and the guidelines of PREPARE and ARRIVEs. The experimental protocol was positively evaluated by the Institutional Protocol Evaluation Committee, and it was licensed under the registered code 6381/11-12-2017 by the Official Veterinary Authorities of Attiki’s Prefecture. Animals’ welfare was assessed by the competent users and was supervised daily by the members of Institutional Welfare Body.

### 2.8. Vaccination Schedule

**Figure 1:**
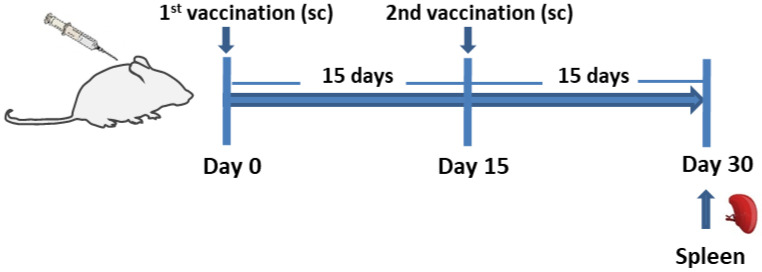
Vaccination schedule. BALB/c mice were vaccinated subcutaneously twice, with 15 days intervals, with (i) PLGA-LeishChim, (ii) PLGA-LeishChim-MPLA or (iii) soluble LeishChim and 15 days post booster vaccination, spleens were removed.

BALB/c mice (*n* = 5 animals/group) were vaccinated subcutaneously into the scruff with (i) PLGA-LeishChim, (ii) PLGA-LeishChim-MPLA or (iii) soluble LeishChim twice with 15 days interval. Mice injected with PBS served as control group. Each mouse received 2.5 µg of the chimeric protein and 3 µg of MPLA encapsulated in 300 µg of PLGA. Soluble LeishChim was given at similar dose to that encapsulated into PLGA NPs. Fifteen days post booster vaccination, spleens were collected from 5 mice from each group, and spleen cells were used for determining cellular immune responses (Figure 1).

### 2.9. LeishChim-Specific Spleen Cell Proliferation Assay

Spleens were aseptically removed, and single cell suspensions were cultured in triplicates in 96-well round-bottom plates at a density of 2 × 10^5^ cells/200 µL/well in the presence of LeishChim (5 µg/mL) for 96 h. Cells exposed to ConA (6 µg/mL) or medium alone served as positive and negative control of the assay, respectively. Cells proliferation was determined by measuring [^3^H]-TdR (Perkin-Elmer, Boston, MA, USA) incorporation during the last 18 h of the culture period on a microplate scintillation β-counter (Microbeta Trilux, Wallac, Turcu, Finland). Results were obtained as counts per minute (cpm) and presented as stimulation index (S.I.). S.I. was calculated according to the following formula: S.I. = cpm measured in lymphocytes in the presence of antigen or mitogen / cpm measured in lymphocytes in medium alone.

### 2.10. Flow Cytometry Analysis for Determination of LeishChim-Specific Memory and Cytokine-Producing T Cells

Single spleen cell suspensions were cultured in 24-well cell culture plates at a density of 1 × 10^6^ cells/mL in the presence of 5 µg/mL LeishChim for 24 h. Next, the cells were triple-stained with anti-CD44-APC (clone IM7), anti-CD62L-PE (clone MEL-14) and anti-CD4-PerCP-Cy5.5 (clone RM4-5) or anti-CD8-PerCP-Cy5.5 (clone 53–6.7) mAbs at a dilution of 1:100 in PBS supplemented with 3% *v*/*v* FBS (FACS buffer) and were incubated for 30 min at 4 °C in the dark. For intracellular cytokine staining, cells were incubated with 10 µg/mL Brefeldin A (Cayman, Ann Arbor, MI, USA) for the last 4 h of incubation, were fixed with paraformaldehyde (2% *w*/*w*) and were permeabilized with FACS buffer/0.1% saponin. Subsequently, spleen cells were stained with anti-IFNγ-PE (clone XMG1.2) and anti-TNF-α-FITC (clone MP6-XT22). All antibodies used were from Biolegend. Cells were analyzed using a FACS Calibur system (Becton-Dickinson, San Jose, CA, USA) running CellQuest software. Data were analyzed using FlowJo software version 10.0 (Tree Star Inc., Ashland, OR, USA).

### 2.11. Statistical Analysis

All results are expressed as mean ± standard deviation (SD). GraphPad Prism version 6.0 software (San Diego, CA, USA) was used for statistical analysis. One-way ANOVA with multiple-comparisons Tukey–Kramer post-test or two-way ANOVA with multiple-comparisons Bonferroni post-test were performed, when required, in order to assess statistically significant differences among experimental groups. A value of *p* < 0.05 was considered significant for all analyses.

## 3. Results

### 3.1. Conserved Leishmania Protein Retrieval and Identification of Cellular Localization

In order to identify proteins that could be used as vaccine candidates against all clinical forms of leishmaniasis, the whole proteome of *L. infantum*, *L. major* and *L. braziliensis* was retrieved as FASTA format from TriTrypDB database. Analysis with BLASTp unveiled 232 proteins showing >80% homology among the three species. According to subcellular localization, 14 proteins were identified as extracellular, 11 as plasma membrane proteins, 56 as cytoplasmic, 70 as nuclear proteins and 74 as mitochondrial proteins, while only 2 proteins were identified as secreted (LinJ.31.0930 and LinJ.31.0960) (Table S1).

### 3.2. Antigenicity and Allergenicity Evaluation

An important step in a pipeline that aims to the design of a vaccine candidate protein is the antigenicity prediction. For that reason, the antigenicity of the retrieved proteins was predicted with the use of VaxiJen and AntigenPro servers. According to the results obtained, 66 proteins were identified as antigenic with values > 0.5 in both algorithms. Next, these proteins were evaluated as potential allergens, since candidate antigens should be non-allergens for hosts. Proteins that were predicted as probable allergens even in one of the three servers used (AllerTop v.2.0, AlgPred και AllergenFP v.1.0.) were excluded from further study. The evaluation resulted in 26 antigenic non-allergenic proteins. Eleven of these proteins were nuclear including ribosomal proteins and RNA polymerases, seven were cytoplasmic among which were three isoforms of beta tubulin, six were ribosomal proteins localized in mitochondria and two proteins were extracellular. Out of the 26 proteins, 4 were hypothetical or of unknown function (Table 1).

### 3.3. MHC I- and II-Binding Epitope Identification, Selection and Vaccine Construction

The inclusion of specific epitopes that bind to MHCI or MHCII molecules in chimeric constructs increase the possibility of elicitation of specific humoral and cellular immune responses, a prerequisite for a successful vaccine candidate. For that reason, epitope analysis was conducted in the 26 selected proteins using IEDB, NetMHCpan, NetMHCIIpan and SYFPEITHI algorithms. Epitopes identified by all three algorithms for human and mouse MHCI and MHCII alleles were selected (File S2, Table 2).

**Figure 2:**
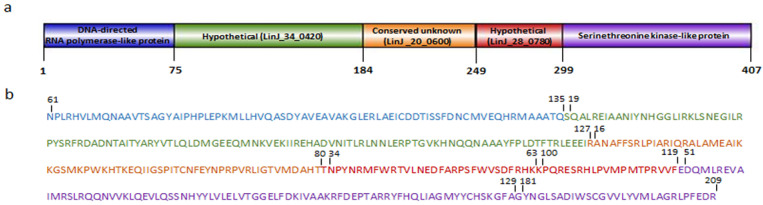
Schematic representation of the LeishChim construct. (**a**) The construct is composed of sequences obtained from 5 different proteins which were placed in tandem. (**b**) Amino acid sequence of LeishChim. Different font colors correspond to each protein from which sequence was derived and numbers indicate which part of each protein was included in LeishChim. Illustrator for Biological sequences (IBS) version 1.0 used for protein illustration is available at http://ibs.biocuckoo.org/online.php#, accessed on 5 December 2018 [52].

The selected MHCI- and MHCII-binding epitopes were further analyzed in order to exclude those showing >45% homology against human or murine amino acid sequences, so that autoimmune or allergic reactions to be avoided. The protein epitopes that showed low homology and were recognized from BALB/c mouse and/or human MHCI or MHCII molecules were further selected (Table S2). Moving down our pipeline, the number of proteins serving as the pool for epitope selection was further reduced to nine, which included those with >5 MHCI and MHCII epitopes, which in total covered >20% of protein’s sequence and, moreover, showed <45% of homology to human and murine sequence. Finally, amino acid sequences from proteins DNA-directed RNA polymerase-like protein (LinJ.28.2200), Hypothetical protein (LinJ.34.0420), Conserved unknown (LinJ.20.0600), Hypothetical protein (LinJ.28.0780) and Serine/threonine kinase-like protein (LinJ.18.0650) which included neighboring or overlapping epitopes strongly binding to MHCI and or MHCII molecules were selected (Table 3). Subsequently, the sequences were fused in tandem resulting in a 407-amino-acid construct consisting of 11 MHCI and 50 MHCII epitopes (Figure 2) that will be referred to as LeishChim.

### 3.4. Immunoinformatics Evaluation of Chimeric Protein

#### 3.4.1. Physicochemical Properties, Antigenicity and Allergenicity Evaluation

The physicochemical properties of a protein reflect its stability, solubility or the difficulty of development and, subsequently, its suitability as a vaccine candidate. For that reason, physicochemical characterization of the chimeric multi-epitope protein was conducted using Protparam server. The molecular weight (MW) of LeishChim was computed as 46,757.76 Da. The protein was predicted as basic with a theoretical pI value of 9.05. The instability index was computed to be 45.65, while the aliphatic index was determined as 85.11. The GRAVY value for LeishChim was determined as −0.338, indicating that the protein is hydrophilic. The half-life, which is related to the stability of the protein, was 1.4 h in mammalian cells in vitro and >10 h in *E. coli* in vivo (Table 4).

The results retrieved from AllerTop v.2.0, AlgPred and AllergenFP v.1.0 servers characterized LeishChim protein as a non-allergen, suggesting that when used as a vaccine candidate, it will not induce autoimmune or allergic reactions.

The probability of LeishChim antigenicity was predicted at 0.50 by AntigenPro and 0.59 by VaxiJen, indicating possible stimulation of efficient immune responses.

#### 3.4.2. Secondary and Tertiary Structure

**Figure 3:**
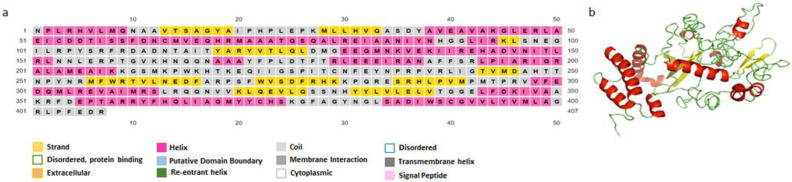
Secondary and tertiary structure of LeishChim. (**a**) PSIPRED was used to predict its secondary structure. (**b**) The tertiary structure was predicted with i-TASSER algorithm, and the best model had a C-score of −2.42.

The PSIPRED algorithm was applied to predict LeishChim’s secondary structure. According to the results obtained, LeishChim consisted of 42.02% helixes, 41.03% coils και 16.95% strands (Figure 3). Subsequently, the 3D model of the chimeric protein was generated using the i-TASSER server, which introduced the top five models based on C-score. Model 1 was chosen as the best model with the highest C-score value of −2.42.

#### 3.4.3. Refinement and Validation of the Tertiary Structure

**Figure 4:**
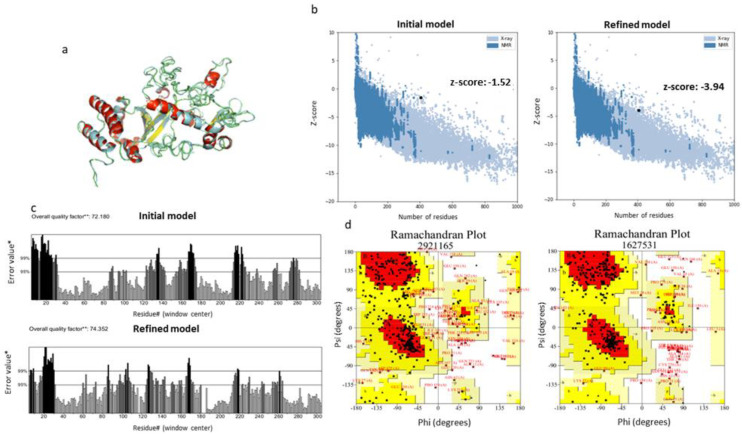
Refinement and validation of LeishChim’s tertiary structure. (**a**) Protein’s refined model via application of GalaxyRefine server. The new structure is pictured in grey color. (**b**) Validation of the initial and the refined tertiary structure of chimeric protein with PROS-A web server. (**c**) ERRAT analysis before and after the refinement. In the ERRAT plot, regions of the 3D model that can be rejected at 95% confidence level are shown in gray lines and regions, while those that can be rejected at 99% level confidence are depicted in black lines. (**d**) Ramachandran analysis of the initial and refined protein model via PROCHECK server application. The most favored areas are represented with darker color.

Possible errors in the initial models predicted by i-TASSER were assessed using the ProSA-web server. According to the results obtained, the z-score of the initial structure was −1.52, which was not among the scores predicted for native proteins of similar length. For that reason, the predicted model was refined with the GalaxyRefine server (Figure 4a). Indeed, the structure was improved, as the z-score was −3.94 (Figure 4b). The potential errors in the refined model were calculated with the ERRAT server and Ramachandran plots. The ERRAT algorithm showed that LeishChim’s structure model was improved with a value of 74.352 compared to 72.180 predicted before refinement (Figure 4c). Moreover, Ramachandran plot analysis revealed that 67.8% of the amino acids of the refined model were located in the favored regions, 26.9% were located in the allowed regions, while only 5.2% of the amino acids were located in the disallowed regions (Figure 4d).

#### 3.4.4. In Silico Prediction of LeishChim’s Docking onto MHCI and MHCII Molecules

Further, LeishChim’s ability to bind onto MHCI and MHCII molecules was predicted. Particularly, the PatchDock server was used as an indirect way to assess the probability of protein epitopes to be recognized by MHC class I and II molecules and, consequently, to elicit strong T cell responses. For this purpose, four common human MHCI (HLA-A*0201, HLA-A*0101, HLA-B*0702, HLA-B*3501) and MHCII (HLA-DRB1*03:01, HLA-DRB5*01:01, HLA-DRB1*01:01, HLA-DRB3*02:02) molecules were chosen for the analysis. Further refinement with the FireDock server revealed that LeishChim-MHCI/II predicted constructs presented low values of global energy, indicating strong binding of the protein onto MHCI as well as MHCII molecules binding pockets (Table 5). 

#### 3.4.5. Stability Prediction of the LeishChim and MHCI/MHCII Complexes

**Figure 5:**
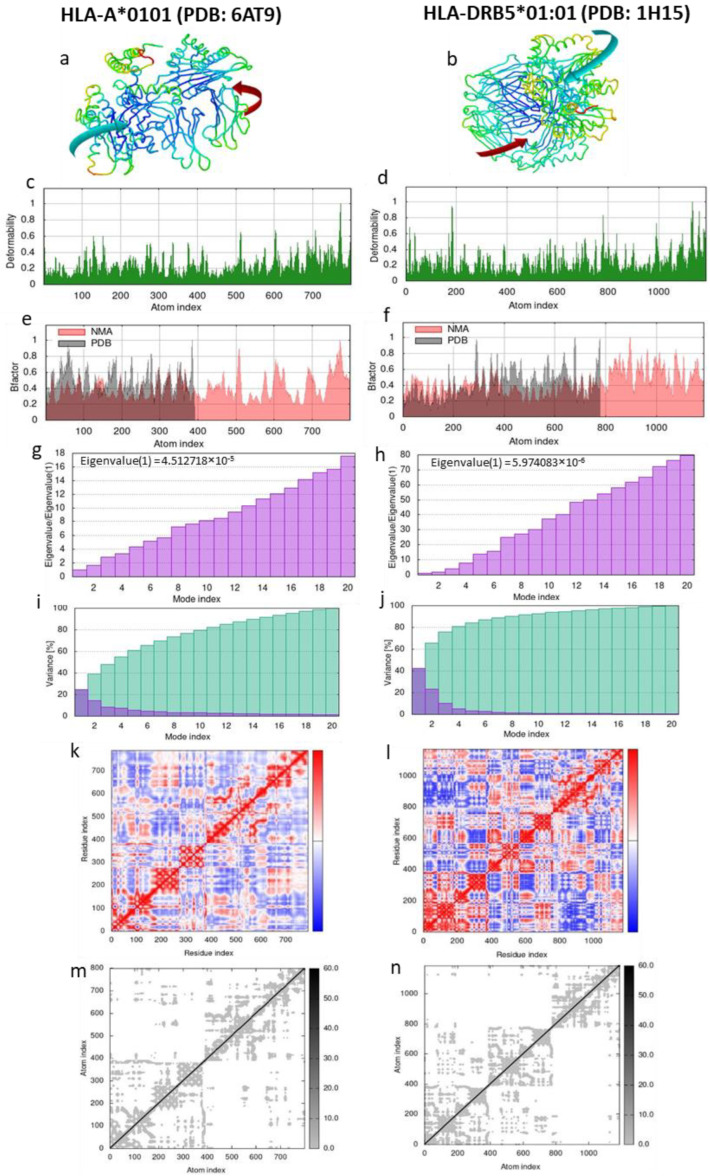
MD simulation of the LeishChim and MHCI or MHCII complexes with Normal Mode Analysis (NMA). (**a**,**b**) NMA mobility of the LeishChim-MHCI/II complexes with affine model arrows. (**c**,**d**) Deformability plots. (**e**,**f**) B-factor plots. (**g**,**h**) Eigenvalue plots. (**i**,**j**) Normal mode variance plots. The purple bars indicate variance of individual modes, while the green indicate cumulative variance. (**k**,**l**) Covariance map. Red, white and blue colors correspond to correlated, uncorrelated and anti-correlated motions. (**m**,**n**) Elastic network. The darker gray colors correspond to stiffer spring.

Molecular dynamics (MD) simulation was performed in order to predict the stability of the complex between the LeishChim protein and MHCI and/or MHCII molecules. HLA-A*0101 (PDB: 6AT9) and HLA-DRB5*01:01 (PDB: 1H15) molecules were chosen since they had the lowest binding energy to the protein according to Table 5. According to NMA analysis, the affine-model-based arrow showed that the LeishChim protein and MHCI or MHCII molecules were directed towards each other, indicating strong binding (Figure 5a,b). The peaks in Figure 5c,d depict the deformability regions of the complexes, while B factor is an index of the protein’s mobility (Figure 5e,f). The eigenvalues that represent the stiffness of the complexes show that LeishChim forms a more stable complex with MHCII molecule compared to MHCI (Figure 5g,h). In the LeishChim-MHCI complex, approximately 80% of the variance was justified by the first 10 modes (Figure 5i) compared to the first four modes in the LeishChim-MHCII complex (Figure 5j), further supporting the stronger binding of the vaccine candidate with MHCII molecule. Moreover, the coupling between pairs of residues in the covariance matrix were illustrated with red colors, while uncorrelated and anti-correlated motions were illustrated with blue and white colors, respectively (Figure 5k,l). An elastic network model revealed the pairs of atoms linked via springs, where stiffer springs appeared with darker grays (Figure 5m,n).

#### 3.4.6. B Cell Linear and Conformational Epitope Prediction

Apart from cellular immune responses, a potential vaccine candidate should also be able to elicit humoral immune responses as well. For that purpose, the ElliPro server was used for prediction of B cell epitopes. The analysis revealed that LeishChim contained 14 linear and 11 conformational epitopes with average scores of 0.663 and 0.676, respectively, indicating the feasibility for LeishChim to induce humoral responses (Table S3).

#### 3.4.7. CTL and HTL Epitope Prediction

Since LeishChim consisted of epitopes assembled in tandem, we wanted to determine if, upon processing steps in the cell, the MHCI epitopes originally predicted would be generated. NetCTL 1.2 predicted a total of 106 CTL epitopes for LeishChim with 28 of them being recognized from two or more HLA I alleles (Table S4). Importantly, seven out of these epitopes were also predicted in the initial epitope analysis. Regarding MHCII-binding epitopes, the IEDB analysis revealed 11 epitopes, while 7 out of these were predicted in the original analysis. These results implied that LeishChim can be correctly processed by the antigen-presenting machinery of the cell (Table S4).

#### 3.4.8. In Silico Immune Simulation Profile of LeishChim

**Figure 6:**
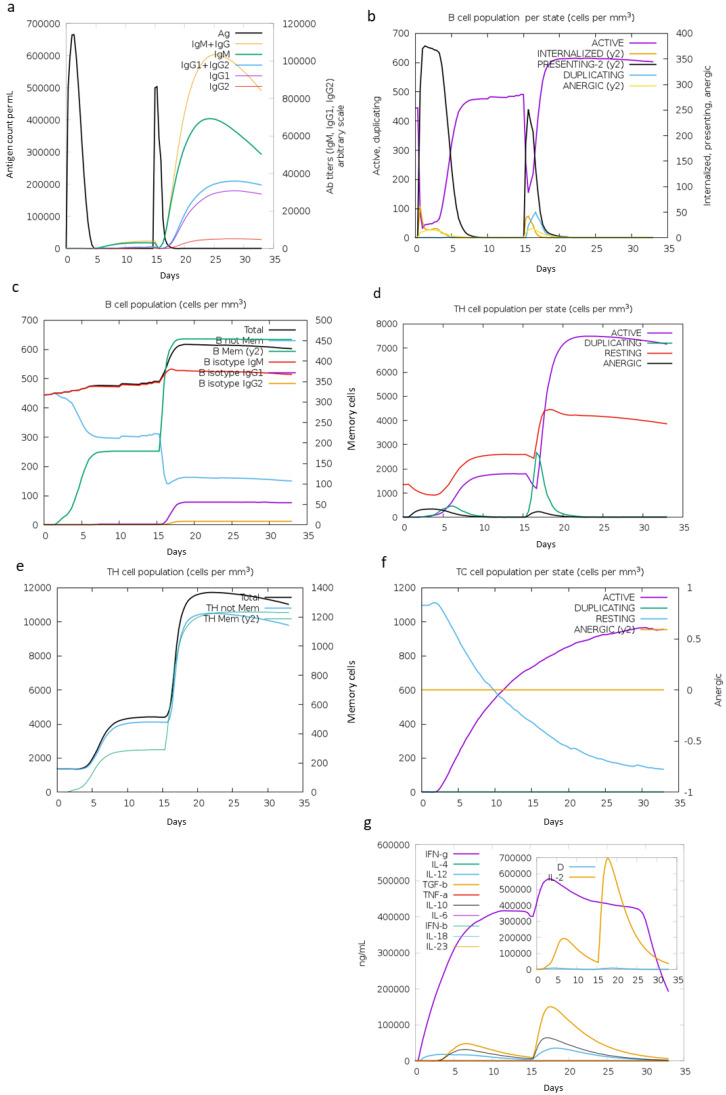
In silico simulation of immune response against LeishChim in a two-immunization protocol. (**a**) Immunoglobulins and antigen levels, (**b**) B cell population per state, (**c**) generation of B cell population, (**d**) T helper (T_H_) cell population per state, (**e**) generation of T_H_ population, (**f**) cytotoxic T (T_C_) cell population per state and (**g**) production of cytokines. The inner graph in (**g**) indicates the Simpson index D of IL-2. Simpson Index D was inferred as measurement of diversity.

The capability of LeishChim to raise humoral and cellular immune responses was eventually determined in silico using the C-ImmSim server. The immune simulation predicted that two immunizations with an interval of 15 days can induce both cellular and humoral immune responses. Specifically, an increase in IgM levels was shown, reflecting the primary immune response to vaccination. Elevated titers of IgG1 and IgG2 antibodies were also detected post second vaccination, indicating increased secondary immune response, while on the subsequent exposure of two injections, decreased levels of antigen were observed (Figure 6a) that could be attributed to the predicted increase in active and memory B cells post first, as well as post second immunization (Figure 6b,c). Importantly, the number of helper and cytotoxic T cells showed similar kinetics (Figure 6d,e), while the increase in memory helper T cells was in accordance with a faster and higher response to second immunization (Figure 6f). This was followed by high levels of IFNγ and IL-2 production, contrary to low IL-10 levels, further suggesting the potential of LeishChim to induce cellular immune responses of the Th1 type.

### 3.5. Immunization with Multi-Epitope Chimeric Protein Encapsulated in PLGA NPs Elicited Antigen-Specific Cellular Immune Responses

LeishChim’s antigenicity was further evaluated in vivo in the experimental model of BALB/c mice. Since a major limitation of multi-epitope constructs is their relatively low immunogenicity, LeishChim was encapsulated into PLGA nanoparticles adjuvanted with the MPLA. The PLGA NPs were characterized concerning their physicochemical properties as well as protein and adjuvant encapsulation and release properties (File S1). The PLGA NPs used had an average diameter of 313.2 ± 6.6 to 345.2 ± 3.0 nm and a negative zeta potential among −43.03 ± 7.68 to −31.30 ± 5.80 mV (File S1). For the purposes of the study, mice were injected twice with a two-week interval with those nanovaccines, and the induction of antigen-specific immune responses was studied two weeks after booster vaccination.

**Figure 7:**
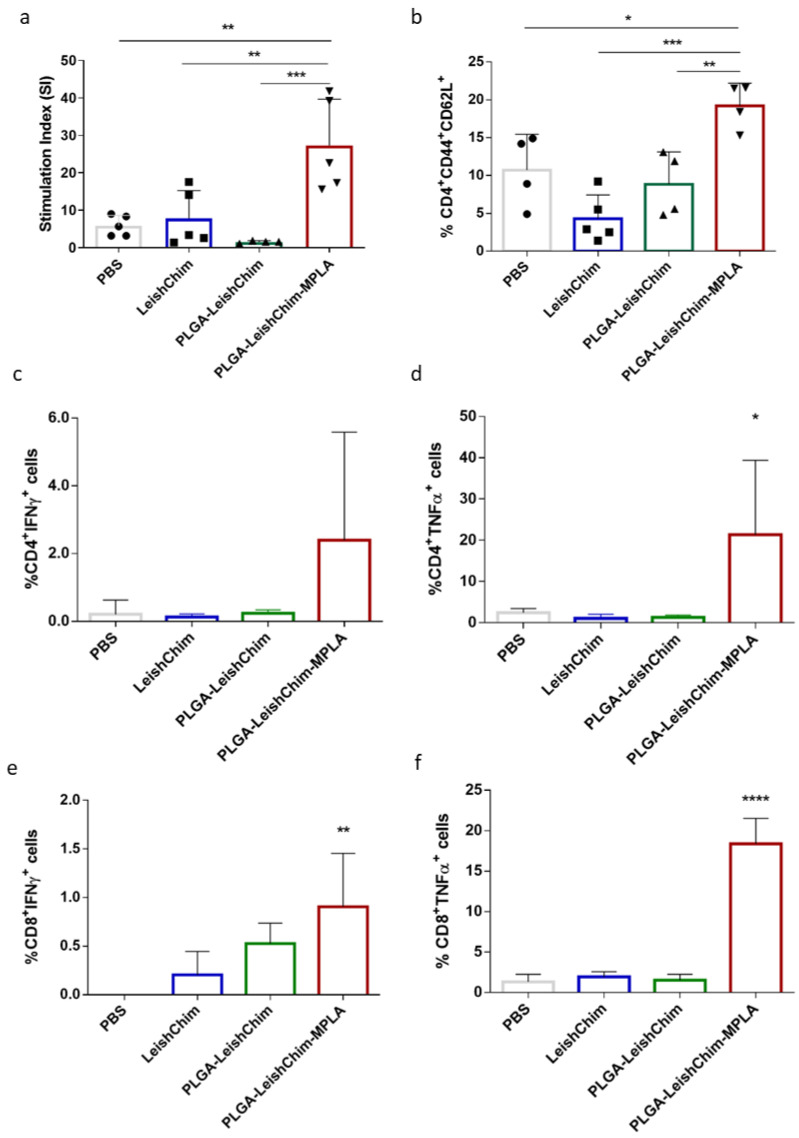
Cellular immune responses in vaccinated BALB/c mice. BALB/c mice (*n* = 5/group) were vaccinated subcutaneously with PBS, LeishChim, PLGA-LeishChim or PLGA-LeishChim-MPLA twice with 15 days interval. (**a**) Fifteen days post booster vaccination, spleen cells were harvested and were stimulated in vitro with LeishChim (5 μg/mL) for 96 h in 5% CO_2_ at 37 °C. LeishChim-specific proliferation was measured by assessing thymidine incorporation. Samples were run in triplicates. In parallel, spleen cells were stimulated with LeishChim (5 μg/mL) for 24 h or 48 h for the detection of (**b**) central memory (CD44^+^CD62L^+^) CD4^+^ T cells and (**c**–**f**) IFNγ- or TNFα-producing CD8^+^ and CD4^+^ T cells with flow cytometry. The results are presented as the mean value ± SD of each group of mice. Significant differences between vaccinated and PBS control group are indicated by asterisks: * *p* < 0.05, ** *p* < 0.01, *** *p* < 0.001 or **** *p* < 0.0001.

The cellular immune responses elicited in vaccinated mice were assessed with the detection of specific anti-LeishChim spleen cells proliferation. According to results, mice vaccinated with PLGA-LeishChim-MPLA exhibited significantly higher proliferation levels in response to LeishChim, compared to non-vaccinated (27.30 ± 12.36 vs. 5.89 ± 2.74, *p* < 0.01), as well as the LeishChim (27.30 ± 12.36 vs. 7.81 ± 7.45, *p* < 0.01) control mice groups, as revealed by stimulation index values (Figure 7a), indicating the development of antigen-specific cells. Of note, spleen cells obtained from mice vaccinated with PLGA-LeishChim exhibited significantly low levels of proliferation comparable to those detected in control mouse groups.

Evaluation of the T cell populations differentiated after vaccination unveiled that the PLGA-LeishChim-MPLA group had developed LeishChim-specific CD4^+^ central memory T cells (CD4^+^CD44^+^CD62L^+^) as compared to the control and PLGA-LeishChim mouse groups (PLGA-LeishChim-MPLA: 19.20 ± 2.99% vs. PBS: 8.89 ± 2.83%, *p* < 0.01) (Figure 7b). Intracellular cytokine staining for IFNγ and TNFα Th1 cytokines revealed that the existence of single-cytokine IFNγ- and TNFα-producing CD4^+^ as well as CD8^+^ T cells in the PLGA-LeishChim-MPLA group were significantly increased compared to PBS (Figure 7c–f). Collectively, the above findings suggested that vaccination with LeishChim encapsulated into PLGA NPs adjuvanted with MPLA induced antigen-specific CD4^+^ as well as CD8^+^ T cells of the Th1 type.

## 4. Discussion

The development of an effective vaccine against leishmaniasis, although highly desirable, has been proven a difficult target due to the complexity of the host–parasite interaction [53,54,55]. First generation vaccines against leishmaniasis included live or attenuated parasites. However, concerns regarding severe side effects made leishmanization unsuitable for human immunization protocols [56]. Moreover, the conventional methods of vaccine preparation involving preclinical as well as clinical studies require complex, time-consuming and expensive protocols for ensuring vaccine efficacy [57]. To overcome these restrictions, sophisticated reverse vaccinology along with proteomics approaches have been adopted to design multi-epitope peptide-based vaccines containing several B and T-cell epitopes [58] able to induce antigen-specific responses [59]. These multi-epitope vaccines have many advantages over the conventional vaccines in terms of potency to cumulatively raise innate, humoral and cellular immune responses [60]. This approach, aiming towards the design of potential vaccines against a variety of pathogens such as bacteria [57,59,60,61,62,63,64,65] and viruses [66,67,68], has successfully led to the design of a commercial vaccine against the serogroup B Neisseria meningitides in 2003 [61,69]. Thus, in the case of visceral leishmaniasis (VL), the most severe form of the disease, several researchers have focused on the development of multi-epitope vaccines exhibiting significant levels of protection against *Leishmania* infection supporting that an efficient vaccine against VL should contain more than one antigen. Specifically, Leish-111f, Leish-110f, KSAC and Q protein multicomponent vaccines have shown to elicit better protective immune responses than vaccines consisted of one protein only [70,71,72,73]. However, until now, none of the designed vaccines have passed clinical trials and entered the market [74]. 

In line with the need for identifying new and effective vaccine candidates that could be able to confront the different clinical forms of the disease, we designed a multi-epitope chimeric protein utilizing immunoinformatics analysis. For this purpose, epitopes were derived from proteins that were chosen prior based on their significant homology >80% among *L. infantum*, *L. major* and *L. braziliensis,* the species causing three different forms of the disease: visceral, cutaneous and mucocutaneous, respectively. The selected proteins were identified using an hierarchical proteome subtractive analysis using the whole proteome of the three different *Leishmania* species, an approach that has been previously applied on the proteome of *L. braziliensis* for the development of an anti-*Leishmania* vaccine [75]. On the contrary, the majority of research targeting the development of multi-epitope vaccines applies bioinformatic analysis using known antigenic *Leishmania* proteins with specific function [76,77,78,79,80,81]. Our analysis unveiled 232 conserved proteins among species which were further shortlisted to 66 based on their antigenicity evaluation using two different algorithms, VaxiJen and AntigenPro. Among the proteins predicted as antigenic were ribosomal proteins, DNA/RNA polymerases and kinases that play a role in parasite growth and viability, transcription factors, mitochondrial proteins, nuclear proteins such histones and a considerable number of hypothetical proteins or proteins with unknown function. Importantly, proteins predicted in silico as antigenic in our study, such as beta tubulin, ribosomal proteins and histones, have already been suggested as immunogenic molecules and have already been tested as potential vaccine candidates against visceral as well as cutaneous leishmaniasis [82,83,84,85]. 

It is well documented that epitopes of an antigen can effectively lead to induction of specific immune responses against the whole antigen. In particular, MHCII epitopes trigger CD4^+^ T_H_ cell activation that release pro-inflammatory cytokines aiming towards B cell activation for antibody production and macrophage activation [86,87].

Moreover, MHCI epitopes are necessary for CD8^+^ T cells that help with infection limitation [88]. T cells identify the peptide epitopes presented by the MHC molecules that are surface proteins of antigen-presenting cells (APCs) via the T cell receptors (TCR), eventually leading to their activation [89]. In the case of *Leishmania*, due to its intracellular nature, T cell responses are vital for controlling diseases. Thus, in the present study, in silico screening of the amino acid sequence of the selected proteins for the identification of MHCI- and MHCII-binding epitopes by applying the IEDB, SYFPEITHI, NetMHCI and NetMHCII servers yielded a significant number of possible epitopes from all five proteins. However, only a few of them were predicted by all algorithms used to have binding efficiency to more than one supertype or allele. The chosen epitopes presented the highest scores regarding MHC-binding as well as large population coverage based on the number of MHCI and II subtypes for which they had high affinity values. Eventually, five *L. infantum* antigenic proteins, namely DNA-directed RNA polymerase-like protein, serine/threonine kinase-like protein, hypothetical proteins LinJ.34.0420 and LinJ.28.0780 and the conserved unknown protein LinJ.20.0600, were selected for chimeric protein design based on their MHCII and MHCI epitope density. Selection of the hypothetical proteins is supported by previous findings suggesting a possible significant role in host invasion or parasite survival of those proteins. Moreover, there are numerous publications proposing hypothetical proteins, either their full sequence or selected epitopes, as candidate vaccine antigens against leishmaniasis [77,90,91,92,93,94,95]. Specifically, it has been shown that spleen cells obtained from mice vaccinated with LiHy produced significant levels of IFNγ after *L. infantum* infection, suggesting it as a potential vaccine candidate [96]. Among the proteins selected in our study was Serine/threonine kinase-like protein. *Leishmania* serine/threonine kinases, such as GSK-3, MAPKs and Aurora kinases, play an important role in the parasite’s survival and replication [97] and some of them have been proposed as potential vaccine candidates [98]. Finally, DNA-directed RNA polymerase-like protein has not yet been studied, to our knowledge, as a vaccine candidate or as part of chimeric vaccine candidates. 

Because single peptides are weak immune stimulators per se and are at risk of degradation by endopeptidase or exopeptidase activity at the injection site or circulation, putting them together in long peptide assemblies reduces the degradation risk and enhances antigenicity [99]. Eventually, in order to design a molecule with increased antigenicity, the shortlisted epitopes were put in tandem to design a chimeric protein molecule, named LeishChim. Since an efficient vaccine should not only induce strong immune responses but should also have acceptable physicochemical and structural properties during the production process, LeishChim’s physicochemical characteristics were first evaluated. According to LeishChim’s in silico characterization, LeishChim was determined in silico to be non-allergenic, proving safety issues related to vaccine. Evaluation of its physicochemical parameters, which have an important impact on vaccination efficiency [100], showed that LeishChim had the appropriate molecular weight of 46,757.76 Da for a vaccine candidate [66,101], with a theoretical pI of 9.05, showing the protein’s basic nature and further suggesting a stable interaction inside the human body [66]. The estimated in vivo half-life (>10 h) was considered to be satisfactory according to previous findings [66,77,101]. Another important parameter is the aliphatic index which is associated with the protein’s thermostability. LeishChim’s value was 85.11, indicating the protein as a thermostable molecule at normal body temperature. Moreover, the negative GRAVY value (−0.338) demonstrated the hydrophilicity of the chimeric protein, which is associated with easier formulation and purification [66]. Overall, the above parameters ensured the thermodynamically stable nature of LeishChim. The initial tertiary structure of the construct was achieved using i-TASSER and was further refined using the GalaxyRefine server. The output model was found to be valid by the ProSA and PROCHECK servers according to Z-score and Ramachandran plot data obtained, respectively. Furthermore, analysis conducted with PathDock as well as MD simulations indicated strong binding of LeishChim to MHCI and MHCII molecules. 

Since the selected MHCI and MHCII epitopes were placed in tandem in the absence of driving linkers for enzymatic digestion and epitope presentation via the MHCI and II pathway [102], LeishChim’s processing into previously identified CTL (MHCI) and HTL (MHCII) epitopes was investigated using the NetCTL and IEDB algorithms. Importantly, among the CTL and HTL epitopes predicted to be contained in LeishChim’s amino acid sequence were included epitopes originally predicted during the first analysis which were chosen for inclusion in the protein construct. Prediction of B cell epitopes with the ElliPro server identified 14 linear and 11 conformational B cell epitopes indicating LeishChim’s efficacy to induce immune responses, including, apart from cellular, and humoral responses. Immune simulation of LeishChim’s capability to initiate an immune response showed that it can efficiently and strongly induce both T and B cell immune responses. Specifically, the populations of helper and cytotoxic T cells elevated after the first dose and further increased after the second one. Moreover, B cells presented a similar trend, followed by elevated titers of IgM and IgG1 and IgG2 antibodies after the first and second dose, respectively. Of note, vaccination simulation with LeishChim induced production of IFNγ in higher levels compared to IL-10, suggesting that vaccination with LeishChim could induce the T_H_1-type immune response that is protective against leishmaniasis [74]. IFNγ-producing T cells stimulate the activation of macrophages to produce NO and reactive oxygen species that are highly effective in intracellular *Leishmania* amastigotes clearance [103,104]. On the contrary, IL-10 production primarily plays a role in parasite establishment or disease progression through down-modulation of innate as well as acquired immunity via prevention of DC migration in the spleen to activate T cells [105,106] and eventually suppression of T_H_1 cells [107].

The immunoinformatics approach followed in the present study was validated in vivo by vaccinating BALB/c mice with LeishChim encapsulated in PLGA NPs along with MPLA as adjuvant. LeishChim was chosen to be encapsulated into PLGA NPs due to several reasons. First, although chimeric and polypeptide vaccines, such as LeishChim, show strong antigenicity, they usually exhibit low immunogenicity. Thus, a strategy to enhance immunogenicity is to encapsulate them into nanoparticulate carriers [15]. Among them, PLGA particles, the most commonly used polymer-based particles, have been widely used as antigen carriers due to certain characteristics such as prevention of antigen degradation. Moreover, PLGA nanoparticles show significant adjuvanticity attributed to the efficient uptake by professional APCs (reviewed in [108]). Previous studies have shown that PLGA nanoparticles encapsulating *Leishmania* antigens along with different adjuvants, such as MPLA, have been adopted as vaccine candidates against experimental leishmaniasis, highlighting the potential of such nanoformulations [21,23,109,110,111,112]. 

Vaccination results confirmed LeishChim’s antigenicity, since mice vaccination with PLGA-LeishChim-MPLA elicited the differentiation of antigen-specific CD4^+^ and CD8^+^ T cell populations, as evidenced by lymphoproliferation assays and FACS analysis. Further characterization of those populations indicated the induction of central memory CD4^+^ T cells (CD4^+^CD44^+^CD62L^+^) that, based on previous studies, play a role in *Leishmania* parasite control [113,114,115]. Central memory T cell populations have a crucial role in the induction of long-lasting immunity induced by vaccination, which is a prerequisite for a vaccine’s success against leishmaniasis [116,117]. Importantly, those CD4^+^ and CD8^+^ T cells were potent IFNγ and TNFα cytokine producers, indicating the induction of antigen-specific T_H_1 and CTL immune responses, as well. It is known that leishmanicidal mechanisms, such as NO production from macrophages, are activated by IFNγ-producing CD4^+^ T cells and are further enhanced by TNFα [117,118], promoting resistance against parasite infection. Additionally, CD8^+^ T cells have been shown to play an important role in confronting VL, acting better as effector cells for eliminating *Leishmania* [119,120,121]. The development of protecting IFNγ-producing CD8^+^ T cells is induced by PLGA nanoparticles loaded with different *Leishmania* antigens [21,109,110] and is associated with PLGA’s ability to promote extracellular antigen cross-presentation [122,123], as shown by previous studies of ours. 

## 5. Conclusions

In conclusion, the present study, taking advantage of the progression of immunoinformatics, employed different computational approaches and suggested a pipeline for the design and characterization of a multi-epitope chimeric protein retrieving highly conserved proteins among the proteomes of *L. infantum*, *L. major* and *L. braziliensis*. This pipeline may contribute as an efficient strategy for vaccine development against leishmaniasis, as well as other pathogen-related diseases, while it offers the advantage of saving resources compared to in vivo evaluation of a large number of vaccine candidates. The physicochemical characterization of LeishChim showed that this protein was stable, antigenic and non-allergenic, and, thus, compatible with humans or animals. Post-design epitope analysis of the protein construct revealed a significant number of MHCI- and MHCII-binding epitopes, as well as B cell epitopes, further supporting LeishChim’s probability of raising appropriate immune responses to combat *Leishmania* infection. The elicitation of specific cellular immune responses against LeishChim in an experimental murine model advocates over the correctness of the developed pipeline, promoting the notion that further translational research on the proposed vaccine construct may contribute to the development of an experimental vaccine for confronting different clinical forms of leishmaniasis. 

## Figures and Tables

**Table 1:** Characteristics of the *Leishmania* spp. Proteins predicted as candidate antigens.

No	Sequence ID	Function	Cellular Localization ^1^	Antigenicity (VaxiJen ^2^, ANTIGENPro ^3^)
1	LinJ.35.1030	casein kinase, putative	Cytoplasm	0.5737/0.836
2	LinJ.35.1540	rieske iron-sulfuric protein, mitochondrion precursor	Cytoplasm	0.5906/0.785
3	LinJ.08.1290	beta tubulin	Cytoplasm	0.5436/0.764
4	LinJ.21.2240	beta tubulin	Cytoplasm	0.5349/0.780
5	LinJ.36.5660	branch point binding protein, putative	Cytoplasm	0.7933/0.917
6	LinJ.08.1280	beta tubulin	Cytoplasm	0.5334/0.784
7	LinJ.28.0780	hypothetical protein	Cytoplasm	0.5545/0.652
8	LinJ.28.2940	receptor for activated C kinase 1	Extracellular	0.6190/0.814
9	LinJ.22.1300	cyclophilin 6, putative	Extracellular	0.5135/0.775
10	LinJ.16.0470	60S ribosomal protein L21, putative	Mitochondria	0.5437/0.665
11	LinJ.20.0600	conserved protein, unknown function	Mitochondria	0.6737/0.735
12	LinJ.34.0420	hypothetical protein, conserved	Mitochondria	0.5455/0.779
13	LinJ.34.3620	ribosomal protein L14, putative	Mitochondria	0.8221/0.624
14	LinJ.35.3810	60S ribosomal protein L27A/L29, putative	Mitochondria	0.7317/0.754
15	LinJ.24.0040	60S ribosomal protein L17, putative	Mitochondria	0.6032/0.850
16	LinJ.20.0250	transmembrane protein, putative	Nucleus	0.5086/0.576
17	LinJ.18.0650	serine/threonine kinase-like protein, putative	Nucleus	0.5320/0.546
18	LinJ.33.1560	RNA-binding protein, putative	Nucleus	0.5448/0.809
19	LinJ.28.2200	DNA-directed RNA polymerase-like protein	Nucleus	0.5213/0.918
20	LinJ.10.0050	ribosomal protein l35a, putative	Nucleus	0.6450/0.869
21	LinJ.28.0210	Histone H2B variant V	Nucleus	0.5420/0.616
22	LinJ.32.0930	60S ribosomal protein L18a, putative	Nucleus	0.5872/0.509
23	LinJ.33.3340	small nuclear ribonucleoprotein SmD2	Nucleus	0.5678/0.534
24	LinJ.36.6680	40S ribosomal protein S8, putative	Nucleus	0.8525/0.616
25	LinJ.21.0440	Protein of unknown function (DUF667)	Nucleus	0.5903/0.840
26	LinJ.27.1450	DNA-directed RNA polymerase II-like protein	Nucleus	0.5001/0.731

^1^ Cellular localization was predicted with CELLO server. ^2,3^ Antigenicity was predicted with VaxiJen and AntigenPro algorithms.

**Table 2:** Number of predicted epitopes, identified with IEDB, NetMHCpan, NetMHCIIpan and SYFPEITHI algorithms, against human and mouse MHCI and MHCII molecules.

No	Protein (Code)	No of MHCI Epitopes		No of MHCII Epitopes	
		Human	Mouse	Human	Mouse
1	LinJ.08.1290	2	13	4	38
2	LinJ.08.1280	2	13	3	38
3	LinJ.21.2240	2	13	3	40
4	LinJ.22.1300	2	6	4	18
5	LinJ.21.0440	3	6	5	24
6	LinJ.36.6680	3	6	3	61
7	LinJ.16.0470	0	5	0	2
8	LinJ.32.0930	1	5	0	5
9	LinJ.24.0040	2	4	0	9
10	LinJ.34.0420	2	4	1	29
11	LinJ.18.0650	0	4	2	35
12	LinJ.28.2200	3	3	0	21
13	LinJ.28.0780	1	3	0	50
14	LinJ.35.1540	6	3	2	64
15	LinJ.20.0250	5	3	1	37
16	LinJ.36.5660	4	2	3	38
17	LinJ.34.3620	1	2	3	55
18	LinJ.33.1560	3	2	1	10
19	LinJ.33.3340	1	2	5	3
20	LinJ.35.3810	1	1	3	3
21	LinJ.28.0210	1	1	4	44
22	LinJ.10.0050	4	1	0	39
23	LinJ.20.0600	0	1	6	25
24	LinJ.27.1450	3	0	0	6
25	LinJ.35.1030	2	11	4	26
26	LinJ.28.2940	2	5	0	35

**Table 3:** List of the finally selected *Leishmania* antigenic proteins.

No	Protein (Code)	Number of MHCI Epitopes	Number of MHCII Epitopes	(%) Coverage of Amino Acid Sequence	Homology Human/Mouse
1	LinJ.34.0420	4	2	30.8%	no
2	LinJ.18.0650	4	9	23.6%	41.29/42.97
3	LinJ.28.2200	3	4	46.7%	40.26/29.92
4	LinJ.28.0780	3	50	81.1%	no
5	LinJ.20.0600	1	14	38.2%	no

**Table 4:** Physicochemical properties of LeishChim multi-epitope chimeric protein predicted by Protparam server.

Physicochemical Characteristic	Amino Acid Number	Molecular Weight	Instability Index	GRAVY	Half Life	Aliphatic Index	Theoretical pI
Score	407	46,757.76 Da	45.65	−0.338	>10 h (*E. coli*, in vivo)	85.11	9.05

**Table 5:** Binding energies of LeishChim with different HLA alleles.

Protein	HLA Alleles (PDB ID)	Global Energy ^1^	aVdW ^2^	rVdW ^3^	ACE ^4^	HB ^5^
LeishChim	HLA-A*0201 (PDB: 1I4F)	2.37	−4.43	0.79	−0.01	−0.22
	HLA-A*0101 (PDB: 6AT9)	−12.54	−19.50	10.78	3.91	−2.85
	HLA-B*0702 (PDB: 5EO0)	0.00	0.00	0.00	0.00	0.00
	HLA-B*3501 (PDB:2FYY)	5.58	−21.45	13.08	11.14	−0.52
	HLA-DRB1*03:01 (PDB: 1A6A)	−22.06	−40.07	21.56	11.65	−3.75
	HLA-DRB5*01:01 (PDB: 1H15)	−2.69	−34.44	38.98	1.97	−4.19
	HLA-DRB1*01:01 (PDB: 2FSE)	−17.35	−26.01	11.39	6.73	−0.35
	HLA-DRB3*02:02 (PDB: 3C5J)	0.43	−6.65	3.13	2.47	0.00

^1^ Global Energy: the binding energy of the solution. ^2^ aVdW: softened attractive van der Waals energy. ^3^ rVdW: softened repulsive van der Waals energy. ^4^ ACE: atomic contact energy. ^5^ HB: hydrogen and disulfide bonds.

## Data Availability

Not applicable.

## Supplementary Materials

The following supporting information can be downloaded at: https://www.mdpi.com/article/10.3390/vaccines11020304/s1, File S1. Preparation and characterization of PLGA Nanoparticles, File S2. Human and Murine MHCI and MHCII epitopes, Table S1. Subcellular localization of proteins retrieved for TriTypDB database showing >80% homology among *L. infantum*, *L. major* and *L. braziliensis*, Table S2. MHCI and MHCII epitopes selected as candidates for vaccine construct, Table S3. Linear and conformational B cell epitopes predicted for LeishChim with ElliPro server, Table S4. CTL and HTL epitopes predicted for LeishChim with NetCTL and IEDB servers, respectively. Epitopes originally retrieved from *Leishmania* proteins are shown in blue font.
