# Supplementary material for: Immunoinformatics Approach to Design a Multi-Epitope Nanovaccine against Leishmania Parasite: Elicitation of Cellular Immune Responses
Source: Vaccines (Basel). 2023 Jan 30;11(2):304. doi: 10.3390/vaccines11020304 (PMC9960668; doi:10.3390/vaccines11020304)
Supplement: Supplementary file 1 [file vaccines-11-00304-s001.zip › File S1_Revised_Round2.pdf]

Preparation and characterization of PLGA Nanoparticles

Results

Preparation and characterization of PLGA NPs

PLGA NPs with an average diameter in the range of 311.1-345.2 nm and a negative zeta potential value, varying from -31.3 to -43.0 mV (Table 1) were obtained. The observed small decrease in the absolute zeta potential value of the LeishChim loaded PLGA NPs could be due to the presence of LeishChim that partly neutralized the free anionic surface carboxyl groups.

Antigen and adjuvant loading in PLGA NPs

The respective values for LeishChim and MPLA loading and encapsulation efficiency are also presented in Table 1. As can be seen, a medium encapsulation efficiency for LeishChim was obtained independently of the presence of adjuvant.

Table 1. Properties of PLGA nanoparticles. Results are presented as mean ± SD (n=3).

Formulation	Average size (nm)	Zeta potential (mV)	Antigen loading (wt%)	Antigen enc. eff. (%)	MPLA loading (wt%)	MPLA enc. eff. (%)
PLGA	320.6±5.9	-43.03±7.68	-	-	-	-
PLGA-LeishChim	313.2±6.6	-31.30±5.80	0.89±0.05	55.40±2.90	-	-
PLGA-MPLA	311.1±4.2	-36.10±6.10	-	-	1.06±0.00	97.21±0.11
PLGA-LeishChim-MPLA	345.2±3.0	-35.00±6.30	0.75±0.01	46.20±0.45	1.08±0.00	98.47±0.07

In vitro release studies

The in vitro release profiles of LeishChim and MPLA from the PLGA nanoparticles in PBS at 37 °C are shown in Figures 1 and 2, respectively. It is apparent that the release profile of LeishChim was characterized by a burst release followed by a short phase of fast release and a lag phase of minimum release. In more detail, approximately 20% of LeishChim was released from both PLGA-LeishChim (Fig. 1A) and PLGA-LeishChim-MPLA (Fig. 1B) NPs during the first 2 h As can be observed, only 50% of LeishChim was released after 4 weeks (672 h) indicating that the PLGA matrix has not been completely hydrolysed in this time period (Fig. 1). Regarding MPLA, it appeared to exhibit an initial phase of zero release followed by a short phase of fast release and a final lag phase of minimum release. Nevertheless, only ~6% of the total MPLA amount was released in PBS after 4 weeks (672 h) of incubation at 37 °C (Fig. 2).

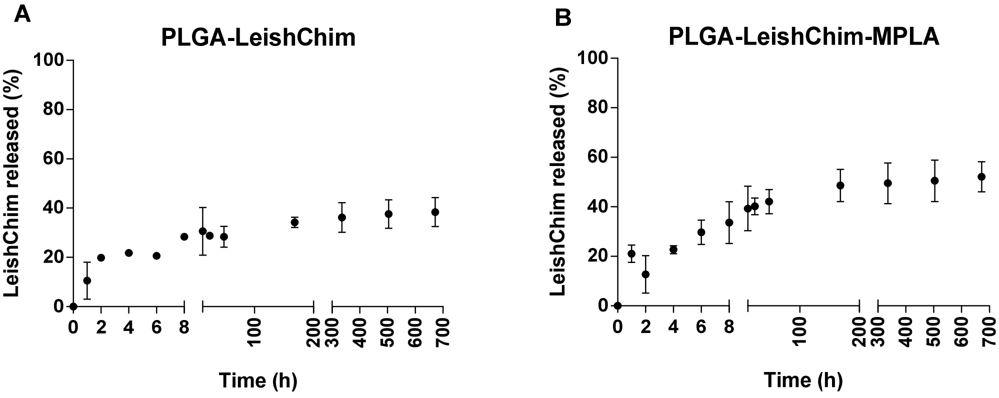

Figure 1. In vitro release profiles of LeishChim from (A) PLGA-LeishChim and (B) PLGA-LeishChim-MPLA in PBS at 37 °C

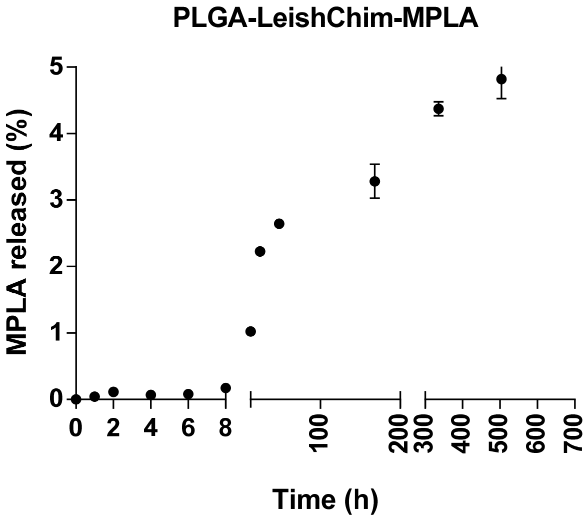

Figure 2. In vitro release profiles of MPLA from PLGA-LeishChim-MPLA NPs in PBS at 37 °C

In vitro stability

The evolution of the PSD of the LeishChim/MPLA loaded PLGA NPs during their incubation in PBS for 4 weeks at 4 °C is shown in Figure 3. No significant changes of the PSD were observed in this time frame, denoting that the PLGA NPs were stable under these experimental conditions. Additionally, the measured zeta potential values of the NPs (Table 1) imply reduced particle aggregation because of the presence of electrostatic forces and thus justify the observed particle stability.

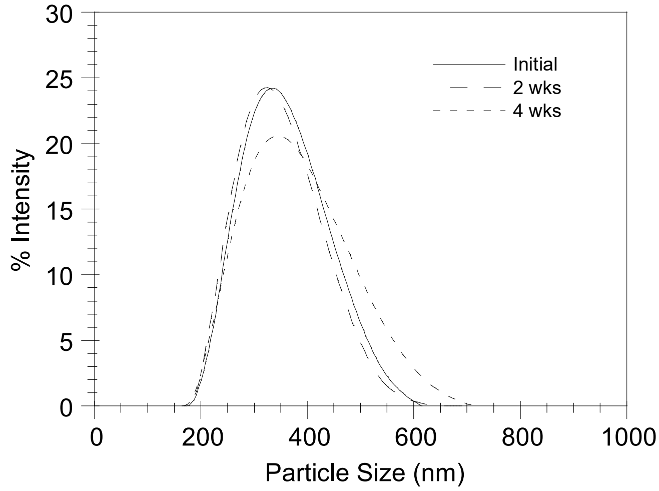

Figure 3. Stability of the PSD of PLGA-LeishChim-MPLA NPs in PBS at 4 °C.
