# Supplementary material for: Immunoinformatics Approach to Design a Multi-Epitope Nanovaccine against Leishmania Parasite: Elicitation of Cellular Immune Responses
Source: Vaccines (Basel). 2023 Jan 30;11(2):304. doi: 10.3390/vaccines11020304 (PMC9960668; doi:10.3390/vaccines11020304)
Supplement: Supplementary file 1 [file vaccines-11-00304-s001.zip › Table S1.pdf]

Table S1. Subcellular localization of proteins retrieved for TriTypDB database showing >80% homology among *L. infantum, L. major* and *L. braziliensis*.

CELLO RESULTS

```
SeqID: li|LinJ_30_2820_mRNA|
       CELLO prediction:
                       (predictor      location        reliable-index)
                       Composition     Mitochondrial   0.545
                       Di-peptide      Mitochondrial   0.821
                       part-Comp.      Mitochondrial   0.556
                       chemo-typy      Cytoplasmic     0.478
                       Neighboring     Cytoplasmic     0.322

       Combined SVM classifier:
                                       Extracellular   0.120
                                       PlasmaMembrane  0.034
                                       Cytoplasmic     1.319
                                       Cytoskeletal    0.020
                                       ER              0.031
                                       Golgi           0.011
                                       Lysosomal               0.019
                                       Mitochondrial   2.287 *
                                       Chloroplast     0.420
                                       Peroxisomal     0.180
                                       Vacuole         0.016
                                       Nuclear         0.544


**********************************************************************
************
SeqID: li|LinJ_36_1160_mRNA|
       CELLO prediction:
                       (predictor      location        reliable-index)
                       Composition     Nuclear         0.424
                       Di-peptide      Nuclear         0.471
                       part-Comp.      Nuclear         0.337
                       chemo-typy      Nuclear         0.693
                       Neighboring     Mitochondrial   0.377

       Combined SVM classifier:
                                       Extracellular   0.160
                                       PlasmaMembrane  0.093
                                       Cytoplasmic     0.587
                                       Cytoskeletal    0.026
                                       ER              0.017
                                       Golgi           0.012
                                       Lysosomal               0.014
                                       Mitochondrial   1.442
                                       Chloroplast     0.351
                                       Peroxisomal     0.078
                                       Vacuole         0.017
                                       Nuclear         2.200 *


**********************************************************************
************
SeqID: li|LinJ_27_2320_mRNA|
       CELLO prediction:
                       (predictor      location        reliable-index)
                       Composition     Mitochondrial   0.397
```

Table S1. Subcellular localization of proteins retrieved for TriTypDB database showing >80% homology among *L. infantum, L. major* and *L. braziliensis*.

```
                       Di-peptide     Nuclear        0.526
                       part-Comp.     Mitochondrial  0.595
                       chemo-typy     Mitochondrial  0.471
                       Neighboring    Nuclear        0.359

        Combined SVM classifier:
                                      Extracellular  0.340
                                      PlasmaMembrane 0.149
                                      Cytoplasmic    0.500
                                      Cytoskeletal   0.023
                                      ER             0.060
                                      Golgi          0.023
                                      Lysosomal              0.023
                                      Mitochondrial  1.992 *
                                      Chloroplast    0.669
                                      Peroxisomal    0.088
                                      Vacuole        0.030
                                      Nuclear        1.103


*********************************************************************
************
SeqID: li|LinJ_18_0650_mRNA|
        CELLO prediction:
                       (predictor     location       reliable-index)
                       Composition    Cytoplasmic    0.337
                       Di-peptide     Nuclear        0.389
                       part-Comp.     Nuclear        0.375
                       chemo-typy     Mitochondrial  0.368
                       Neighboring    Cytoplasmic    0.350

        Combined SVM classifier:
                                      Extracellular  0.095
                                      PlasmaMembrane 0.121
                                      Cytoplasmic    1.582 *
                                      Cytoskeletal   0.015
                                      ER             0.020
                                      Golgi          0.035
                                      Lysosomal              0.018
                                      Mitochondrial  1.303 *
                                      Chloroplast    0.204
                                      Peroxisomal    0.126
                                      Vacuole        0.009
                                      Nuclear        1.471 *


*********************************************************************
************
SeqID: li|LinJ_35_4150_mRNA|
        CELLO prediction:
                       (predictor     location       reliable-index)
                       Composition    Mitochondrial  0.292
                       Di-peptide     Cytoplasmic    0.331
                       part-Comp.     Cytoplasmic    0.339
                       chemo-typy     Mitochondrial  0.604
                       Neighboring    Cytoplasmic    0.378

        Combined SVM classifier:
                                      Extracellular  0.680
                                      PlasmaMembrane 0.031
                                      Cytoplasmic    1.505 *
```

Table S1. Subcellular localization of proteins retrieved for TriTypDB database showing >80% homology among *L. infantum*, *L. major* and *L. braziliensis*.

```
                                        Cytoskeletal    0.027
                                        ER              0.025
                                        Golgi           0.028
                                        Lysosomal               0.012
                                        Mitochondrial   1.374 *
                                        Chloroplast     0.271
                                        Peroxisomal     0.030
                                        Vacuole         0.008
                                        Nuclear         1.009 *


************************************************************************
************
SeqID: li|LinJ_07_0520_mRNA|
        CELLO prediction:
                        (predictor      location        reliable-index)
                        Composition     Mitochondrial   0.457
                        Di-peptide      Cytoplasmic     0.351
                        part-Comp.      PlasmaMembrane  0.503
                        chemo-typy      Nuclear         0.315
                        Neighboring     PlasmaMembrane  0.682

        Combined SVM classifier:
                                        Extracellular   0.338
                                        PlasmaMembrane  1.694 *
                                        Cytoplasmic     0.884
                                        Cytoskeletal    0.054
                                        ER              0.031
                                        Golgi           0.026
                                        Lysosomal               0.024
                                        Mitochondrial   1.049 *
                                        Chloroplast     0.300
                                        Peroxisomal     0.093
                                        Vacuole         0.013
                                        Nuclear         0.494


************************************************************************
************
SeqID: li|LinJ_09_0970_mRNA|
        CELLO prediction:
                        (predictor      location        reliable-index)
                        Composition     Nuclear         0.679
                        Di-peptide      Cytoplasmic     0.897
                        part-Comp.      Cytoplasmic     0.600
                        chemo-typy      Cytoplasmic     0.754
                        Neighboring     Cytoplasmic     0.790

        Combined SVM classifier:
                                        Extracellular   0.033
                                        PlasmaMembrane  0.027
                                        Cytoplasmic     3.232 *
                                        Cytoskeletal    0.012
                                        ER              0.101
                                        Golgi           0.043
                                        Lysosomal               0.004
                                        Mitochondrial   0.065
                                        Chloroplast     0.163
                                        Peroxisomal     0.018
                                        Vacuole         0.021
                                        Nuclear         1.280
```

Table S1. Subcellular localization of proteins retrieved for TriTypDB database showing >80% homology among *L. infantum, L. major* and *L. braziliensis*.

```
*********************************************************************
************
SeqID: li|LinJ_31_3320_mRNA|
       CELLO prediction:
                       (predictor     location       reliable-index)
                       Composition    Cytoplasmic    0.379
                       Di-peptide     Nuclear        0.696
                       part-Comp.     Nuclear        0.615
                       chemo-typy     Nuclear        0.755
                       Neighboring    Nuclear        0.503

       Combined SVM classifier:
                                      Extracellular  0.128
                                      PlasmaMembrane 0.035
                                      Cytoplasmic    0.773
                                      Cytoskeletal   0.013
                                      ER             0.013
                                      Golgi          0.007
                                      Lysosomal              0.008
                                      Mitochondrial  0.940
                                      Chloroplast    0.274
                                      Peroxisomal    0.034
                                      Vacuole        0.016
                                      Nuclear        2.760 *


*********************************************************************
************
SeqID: li|LinJ_03_0240_mRNA|
       CELLO prediction:
                       (predictor     location       reliable-index)
                       Composition    Cytoplasmic    0.341
                       Di-peptide     Mitochondrial  0.610
                       part-Comp.     Nuclear        0.778
                       chemo-typy     Mitochondrial  0.589
                       Neighboring    Nuclear        0.467

       Combined SVM classifier:
                                      Extracellular  0.266
                                      PlasmaMembrane 0.042
                                      Cytoplasmic    0.778
                                      Cytoskeletal   0.018
                                      ER             0.021
                                      Golgi          0.016
                                      Lysosomal              0.010
                                      Mitochondrial  1.639 *
                                      Chloroplast    0.188
                                      Peroxisomal    0.026
                                      Vacuole        0.012
                                      Nuclear        1.983 *


*********************************************************************
************
SeqID: li|LinJ_35_3840_mRNA|
       CELLO prediction:
                       (predictor     location       reliable-index)
                       Composition    Mitochondrial  0.385
                       Di-peptide     Mitochondrial  0.624
```

Table S1. Subcellular localization of proteins retrieved for TriTypDB database showing >80% homology among *L. infantum, L. major* and *L. braziliensis*.

```
                       part-Comp.      Cytoplasmic     0.340
                       chemo-typy      Mitochondrial   0.352
                       Neighboring     Mitochondrial   0.416

        Combined SVM classifier:
                                       Extracellular   0.313
                                       PlasmaMembrane  0.143
                                       Cytoplasmic     1.111
                                       Cytoskeletal    0.013
                                       ER              0.023
                                       Golgi           0.012
                                       Lysosomal               0.020
                                       Mitochondrial   1.937 *
                                       Chloroplast     0.756
                                       Peroxisomal     0.111
                                       Vacuole         0.018
                                       Nuclear         0.543


*********************************************************************
************
SeqID: li|LinJ_22_0720_mRNA|
        CELLO prediction:
                       (predictor      location        reliable-index)
                       Composition     Nuclear         0.547
                       Di-peptide      Nuclear         0.860
                       part-Comp.      Nuclear         0.741
                       chemo-typy      Mitochondrial   0.425
                       Neighboring     Nuclear         0.692

        Combined SVM classifier:
                                       Extracellular   0.070
                                       PlasmaMembrane  0.011
                                       Cytoplasmic     0.527
                                       Cytoskeletal    0.032
                                       ER              0.013
                                       Golgi           0.016
                                       Lysosomal               0.010
                                       Mitochondrial   0.797
                                       Chloroplast     0.432
                                       Peroxisomal     0.057
                                       Vacuole         0.017
                                       Nuclear         3.019 *


*********************************************************************
************
SeqID: li|LinJ_35_0190_mRNA|
        CELLO prediction:
                       (predictor      location        reliable-index)
                       Composition     Nuclear         0.564
                       Di-peptide      Cytoplasmic     0.349
                       part-Comp.      Cytoplasmic     0.314
                       chemo-typy      Chloroplast     0.478
                       Neighboring     Cytoplasmic     0.286

        Combined SVM classifier:
                                       Extracellular   0.533
                                       PlasmaMembrane  0.078
                                       Cytoplasmic     1.441 *
                                       Cytoskeletal    0.021
```

Table S1. Subcellular localization of proteins retrieved for TriTypDB database showing >80% homology among *L. infantum, L. major* and *L. braziliensis*.

```
                                        ER              0.129
                                        Golgi           0.068
                                        Lysosomal               0.081
                                        Mitochondrial   0.275
                                        Chloroplast     0.811
                                        Peroxisomal     0.188
                                        Vacuole         0.047
                                        Nuclear         1.328 *


************************************************************************
************
SeqID: li|LinJ_11_1180_mRNA|
        CELLO prediction:
                        (predictor      location        reliable-index)
                        Composition     Mitochondrial   0.520
                        Di-peptide      Cytoplasmic     0.401
                        part-Comp.      Mitochondrial   0.431
                        chemo-typy      Extracellular   0.310
                        Neighboring     Mitochondrial   0.433

        Combined SVM classifier:
                                        Extracellular   0.468
                                        PlasmaMembrane  0.245
                                        Cytoplasmic     1.045
                                        Cytoskeletal    0.022
                                        ER              0.032
                                        Golgi           0.013
                                        Lysosomal               0.014
                                        Mitochondrial   1.868 *
                                        Chloroplast     0.752
                                        Peroxisomal     0.078
                                        Vacuole         0.015
                                        Nuclear         0.449


************************************************************************
************
SeqID: li|LinJ_33_2710_mRNA|
        CELLO prediction:
                        (predictor      location        reliable-index)
                        Composition     Mitochondrial   0.644
                        Di-peptide      Mitochondrial   0.421
                        part-Comp.      Extracellular   0.341
                        chemo-typy      Mitochondrial   0.427
                        Neighboring     Mitochondrial   0.253

        Combined SVM classifier:
                                        Extracellular   0.811
                                        PlasmaMembrane  0.306
                                        Cytoplasmic     0.559
                                        Cytoskeletal    0.015
                                        ER              0.200
                                        Golgi           0.019
                                        Lysosomal               0.131
                                        Mitochondrial   1.984 *
                                        Chloroplast     0.680
                                        Peroxisomal     0.085
                                        Vacuole         0.026
                                        Nuclear         0.183
```

Table S1. Subcellular localization of proteins retrieved for TriTypDB database showing >80% homology among *L. infantum, L. major* and *L. braziliensis.*

```
*********************************************************************
************
SeqID: li|LinJ_16_0950_mRNA|
       CELLO prediction:
                      (predictor     location       reliable-index)
                      Composition    Cytoplasmic    0.698
                      Di-peptide     Cytoplasmic    0.749
                      part-Comp.     Cytoplasmic    0.429
                      chemo-typy     Nuclear        0.446
                      Neighboring    Cytoplasmic    0.750

       Combined SVM classifier:
                                     Extracellular  0.227
                                     PlasmaMembrane 0.124
                                     Cytoplasmic    3.034 *
                                     Cytoskeletal   0.010
                                     ER             0.027
                                     Golgi          0.025
                                     Lysosomal             0.022
                                     Mitochondrial  0.565
                                     Chloroplast    0.144
                                     Peroxisomal    0.039
                                     Vacuole        0.009
                                     Nuclear        0.775


*********************************************************************
************
SeqID: li|LinJ_35_2250_mRNA|
       CELLO prediction:
                      (predictor     location       reliable-index)
                      Composition    Nuclear        0.273
                      Di-peptide     Mitochondrial  0.401
                      part-Comp.     Nuclear        0.554
                      chemo-typy     Nuclear        0.406
                      Neighboring    Extracellular  0.302

       Combined SVM classifier:
                                     Extracellular  0.673
                                     PlasmaMembrane 0.070
                                     Cytoplasmic    1.243 *
                                     Cytoskeletal   0.048
                                     ER             0.029
                                     Golgi          0.016
                                     Lysosomal             0.012
                                     Mitochondrial  0.827
                                     Chloroplast    0.269
                                     Peroxisomal    0.040
                                     Vacuole        0.020
                                     Nuclear        1.755 *


*********************************************************************
************
SeqID: li|LinJ_30_3060_mRNA|
       CELLO prediction:
                      (predictor     location       reliable-index)
                      Composition    Chloroplast    0.379
                      Di-peptide     Cytoplasmic    0.325
                      part-Comp.     Cytoplasmic    0.473
```

Table S1. Subcellular localization of proteins retrieved for TriTypDB database showing >80% homology among *L. infantum, L. major* and *L. braziliensis*.

```
                       chemo-typy     Cytoplasmic    0.500
                       Neighboring    Chloroplast    0.470

        Combined SVM classifier:
                                      Extracellular  0.309
                                      PlasmaMembrane 0.072
                                      Cytoplasmic    1.650 *
                                      Cytoskeletal   0.016
                                      ER             0.073
                                      Golgi          0.042
                                      Lysosomal              0.020
                                      Mitochondrial  0.739
                                      Chloroplast    1.389 *
                                      Peroxisomal    0.152
                                      Vacuole        0.070
                                      Nuclear        0.468


*********************************************************************
************
SeqID: li|LinJ_35_1900_mRNA|
        CELLO prediction:
                       (predictor     location       reliable-index)
                       Composition    Cytoplasmic    0.722
                       Di-peptide     Nuclear        0.581
                       part-Comp.     Nuclear        0.355
                       chemo-typy     Nuclear        0.917
                       Neighboring    Nuclear        0.318

        Combined SVM classifier:
                                      Extracellular  0.072
                                      PlasmaMembrane 0.026
                                      Cytoplasmic    1.514
                                      Cytoskeletal   0.020
                                      ER             0.016
                                      Golgi          0.009
                                      Lysosomal              0.010
                                      Mitochondrial  0.829
                                      Chloroplast    0.146
                                      Peroxisomal    0.031
                                      Vacuole        0.013
                                      Nuclear        2.315 *


*********************************************************************
************
SeqID: li|LinJ_22_1040_mRNA|
        CELLO prediction:
                       (predictor     location       reliable-index)
                       Composition    Mitochondrial  0.706
                       Di-peptide     Nuclear        0.548
                       part-Comp.     Mitochondrial  0.903
                       chemo-typy     Mitochondrial  0.582
                       Neighboring    Mitochondrial  0.828

        Combined SVM classifier:
                                      Extracellular  0.120
                                      PlasmaMembrane 0.022
                                      Cytoplasmic    0.279
                                      Cytoskeletal   0.008
                                      ER             0.009
```

Table S1. Subcellular localization of proteins retrieved for TriTypDB database showing >80% homology among *L. infantum, L. major* and *L. braziliensis.*

```
                                       Golgi           0.003
                                       Lysosomal               0.014
                                       Mitochondrial   3.335 *
                                       Chloroplast     0.176
                                       Peroxisomal     0.055
                                       Vacuole         0.005
                                       Nuclear         0.975


**********************************************************************
************
SeqID: li|LinJ_33_1560_mRNA|
        CELLO prediction:
                        (predictor     location        reliable-index)
                        Composition    Nuclear         0.551
                        Di-peptide     Nuclear         0.439
                        part-Comp.     Nuclear         0.344
                        chemo-typy     Nuclear         0.694
                        Neighboring    Nuclear         0.736

        Combined SVM classifier:
                                       Extracellular   0.251
                                       PlasmaMembrane  0.207
                                       Cytoplasmic     0.660
                                       Cytoskeletal    0.043
                                       ER              0.024
                                       Golgi           0.017
                                       Lysosomal               0.023
                                       Mitochondrial   0.749
                                       Chloroplast     0.118
                                       Peroxisomal     0.091
                                       Vacuole         0.054
                                       Nuclear         2.763 *


**********************************************************************
************
SeqID: li|LinJ_36_0950_mRNA|
        CELLO prediction:
                        (predictor     location        reliable-index)
                        Composition    Nuclear         0.363
                        Di-peptide     PlasmaMembrane  0.317
                        part-Comp.     Extracellular   0.318
                        chemo-typy     Cytoplasmic     0.309
                        Neighboring    Cytoplasmic     0.260

        Combined SVM classifier:
                                       Extracellular   0.855
                                       PlasmaMembrane  0.948
                                       Cytoplasmic     0.985 *
                                       Cytoskeletal    0.022
                                       ER              0.064
                                       Golgi           0.144
                                       Lysosomal               0.042
                                       Mitochondrial   0.312
                                       Chloroplast     0.509
                                       Peroxisomal     0.131
                                       Vacuole         0.042
                                       Nuclear         0.946
```

Table S1. Subcellular localization of proteins retrieved for TriTypDB database showing >80% homology among *L. infantum, L. major* and *L. braziliensis*.

```
*********************************************************************
************
SeqID: li|LinJ_26_0840_mRNA|
       CELLO prediction:
                       (predictor      location        reliable-index)
                       Composition     Cytoplasmic     0.404
                       Di-peptide      Mitochondrial   0.743
                       part-Comp.      Mitochondrial   0.538
                       chemo-typy      Mitochondrial   0.383
                       Neighboring     Mitochondrial   0.670

       Combined SVM classifier:
                                       Extracellular   0.159
                                       PlasmaMembrane  0.040
                                       Cytoplasmic     1.402
                                       Cytoskeletal    0.010
                                       ER              0.012
                                       Golgi           0.008
                                       Lysosomal               0.015
                                       Mitochondrial   2.698 *
                                       Chloroplast     0.182
                                       Peroxisomal     0.104
                                       Vacuole         0.011
                                       Nuclear         0.359


*********************************************************************
************
SeqID: li|LinJ_30_3650_mRNA|
       CELLO prediction:
                       (predictor      location        reliable-index)
                       Composition     Cytoplasmic     0.360
                       Di-peptide      Chloroplast     0.354
                       part-Comp.      Cytoplasmic     0.877
                       chemo-typy      Cytoplasmic     0.772
                       Neighboring     Cytoplasmic     0.432

       Combined SVM classifier:
                                       Extracellular   0.085
                                       PlasmaMembrane  0.024
                                       Cytoplasmic     2.673 *
                                       Cytoskeletal    0.014
                                       ER              0.014
                                       Golgi           0.009
                                       Lysosomal               0.016
                                       Mitochondrial   0.702
                                       Chloroplast     0.713
                                       Peroxisomal     0.054
                                       Vacuole         0.023
                                       Nuclear         0.674


*********************************************************************
************
SeqID: li|LinJ_30_3280_mRNA|
       CELLO prediction:
                       (predictor      location        reliable-index)
                       Composition     Mitochondrial   0.456
                       Di-peptide      Mitochondrial   0.453
                       part-Comp.      Mitochondrial   0.605
                       chemo-typy      Mitochondrial   0.625
```

Table S1. Subcellular localization of proteins retrieved for TriTypDB database showing >80% homology among *L. infantum, L. major* and *L. braziliensis*.

```
                       Neighboring    Mitochondrial  0.484

       Combined SVM classifier:
                                      Extracellular  0.132
                                      PlasmaMembrane 0.037
                                      Cytoplasmic    0.750
                                      Cytoskeletal   0.018
                                      ER             0.120
                                      Golgi          0.020
                                      Lysosomal              0.056
                                      Mitochondrial  2.623 *
                                      Chloroplast    0.469
                                      Peroxisomal    0.044
                                      Vacuole        0.029
                                      Nuclear        0.702


**********************************************************************
************
SeqID: li|LinJ_17_1380_mRNA|
       CELLO prediction:
                       (predictor     location       reliable-index)
                       Composition    Extracellular  0.333
                       Di-peptide     Mitochondrial  0.424
                       part-Comp.     Chloroplast    0.252
                       chemo-typy     Mitochondrial  0.427
                       Neighboring    Chloroplast    0.360

       Combined SVM classifier:
                                      Extracellular  0.775
                                      PlasmaMembrane 0.642
                                      Cytoplasmic    0.481
                                      Cytoskeletal   0.025
                                      ER             0.043
                                      Golgi          0.019
                                      Lysosomal              0.050
                                      Mitochondrial  1.357 *
                                      Chloroplast    1.108 *
                                      Peroxisomal    0.207
                                      Vacuole        0.041
                                      Nuclear        0.252


**********************************************************************
************
SeqID: li|LinJ_34_2100_mRNA|
       CELLO prediction:
                       (predictor     location       reliable-index)
                       Composition    PlasmaMembrane 0.479
                       Di-peptide     Mitochondrial  0.233
                       part-Comp.     Cytoplasmic    0.423
                       chemo-typy     Mitochondrial  0.438
                       Neighboring    Nuclear        0.405

       Combined SVM classifier:
                                      Extracellular  0.147
                                      PlasmaMembrane 1.083 *
                                      Cytoplasmic    1.189 *
                                      Cytoskeletal   0.032
                                      ER             0.031
                                      Golgi          0.254
```

Table S1. Subcellular localization of proteins retrieved for TriTypDB database showing >80% homology among *L. infantum, L. major* and *L. braziliensis*.

```
                                        Lysosomal               0.015
                                        Mitochondrial   0.812
                                        Chloroplast     0.362
                                        Peroxisomal     0.063
                                        Vacuole         0.016
                                        Nuclear         0.996


**********************************************************************
************
SeqID: li|LinJ_13_1410_mRNA|
        CELLO prediction:
                        (predictor      location        reliable-index)
                        Composition     Cytoplasmic     0.647
                        Di-peptide      Nuclear         0.726
                        part-Comp.      Cytoplasmic     0.763
                        chemo-typy      Nuclear         0.844
                        Neighboring     Cytoplasmic     0.655

        Combined SVM classifier:
                                        Extracellular   0.145
                                        PlasmaMembrane  0.040
                                        Cytoplasmic     2.233 *
                                        Cytoskeletal    0.011
                                        ER              0.024
                                        Golgi           0.010
                                        Lysosomal               0.013
                                        Mitochondrial   0.550
                                        Chloroplast     0.061
                                        Peroxisomal     0.027
                                        Vacuole         0.018
                                        Nuclear         1.869 *


**********************************************************************
************
SeqID: li|LinJ_36_0370_mRNA|
        CELLO prediction:
                        (predictor      location        reliable-index)
                        Composition     Mitochondrial   0.367
                        Di-peptide      Nuclear         0.755
                        part-Comp.      Nuclear         0.573
                        chemo-typy      Nuclear         0.508
                        Neighboring     Mitochondrial   0.452

        Combined SVM classifier:
                                        Extracellular   0.156
                                        PlasmaMembrane  0.045
                                        Cytoplasmic     0.911
                                        Cytoskeletal    0.020
                                        ER              0.017
                                        Golgi           0.010
                                        Lysosomal               0.010
                                        Mitochondrial   1.306
                                        Chloroplast     0.318
                                        Peroxisomal     0.030
                                        Vacuole         0.010
                                        Nuclear         2.168 *
```

Table S1. Subcellular localization of proteins retrieved for TriTypDB database showing >80% homology among *L. infantum, L. major* and *L. braziliensis*.

```
**********************************************************************
************
SeqID: li|LinJ_36_1310_mRNA|
       CELLO prediction:
                       (predictor     location       reliable-index)
                       Composition    Nuclear        0.635
                       Di-peptide     Nuclear        0.646
                       part-Comp.     Nuclear        0.665
                       chemo-typy     Nuclear        0.816
                       Neighboring    Mitochondrial  0.511

       Combined SVM classifier:
                                      Extracellular  0.030
                                      PlasmaMembrane 0.032
                                      Cytoplasmic    0.461
                                      Cytoskeletal   0.014
                                      ER             0.004
                                      Golgi          0.005
                                      Lysosomal              0.003
                                      Mitochondrial  1.023
                                      Chloroplast    0.178
                                      Peroxisomal    0.036
                                      Vacuole        0.004
                                      Nuclear        3.209 *


**********************************************************************
************
SeqID: li|LinJ_35_1320_mRNA|
       CELLO prediction:
                       (predictor     location       reliable-index)
                       Composition    Cytoplasmic    0.517
                       Di-peptide     Nuclear        0.722
                       part-Comp.     Nuclear        0.501
                       chemo-typy     Nuclear        0.793
                       Neighboring    Nuclear        0.455

       Combined SVM classifier:
                                      Extracellular  0.108
                                      PlasmaMembrane 0.036
                                      Cytoplasmic    1.013
                                      Cytoskeletal   0.013
                                      ER             0.013
                                      Golgi          0.007
                                      Lysosomal              0.008
                                      Mitochondrial  0.907
                                      Chloroplast    0.234
                                      Peroxisomal    0.032
                                      Vacuole        0.014
                                      Nuclear        2.615 *


**********************************************************************
************
SeqID: li|LinJ_36_5350_mRNA|
       CELLO prediction:
                       (predictor     location       reliable-index)
                       Composition    Mitochondrial  0.638
                       Di-peptide     Cytoplasmic    0.400
                       part-Comp.     Mitochondrial  0.449
                       chemo-typy     Cytoplasmic    0.545
```

Table S1. Subcellular localization of proteins retrieved for TriTypDB database showing >80% homology among *L. infantum, L. major* and *L. braziliensis*.

```
                       Neighboring     Cytoplasmic     0.527

        Combined SVM classifier:
                                       Extracellular   0.078
                                       PlasmaMembrane  0.083
                                       Cytoplasmic     1.684 *
                                       Cytoskeletal    0.027
                                       ER              0.024
                                       Golgi           0.020
                                       Lysosomal               0.008
                                       Mitochondrial   2.088 *
                                       Chloroplast     0.476
                                       Peroxisomal     0.355
                                       Vacuole         0.009
                                       Nuclear         0.148


**********************************************************************
************
SeqID: li|LinJ_26_0540_mRNA|
        CELLO prediction:
                       (predictor      location        reliable-index)
                       Composition     Extracellular   0.401
                       Di-peptide      Extracellular   0.806
                       part-Comp.      Extracellular   0.446
                       chemo-typy      Chloroplast     0.258
                       Neighboring     Cytoplasmic     0.269

        Combined SVM classifier:
                                       Extracellular   2.103 *
                                       PlasmaMembrane  0.313
                                       Cytoplasmic     0.726
                                       Cytoskeletal    0.015
                                       ER              0.111
                                       Golgi           0.080
                                       Lysosomal               0.075
                                       Mitochondrial   0.195
                                       Chloroplast     0.570
                                       Peroxisomal     0.038
                                       Vacuole         0.165
                                       Nuclear         0.609


**********************************************************************
************
SeqID: li|LinJ_20_0600_mRNA|
        CELLO prediction:
                       (predictor      location        reliable-index)
                       Composition     Mitochondrial   0.848
                       Di-peptide      Mitochondrial   0.917
                       part-Comp.      Mitochondrial   0.863
                       chemo-typy      Mitochondrial   0.501
                       Neighboring     Mitochondrial   0.893

        Combined SVM classifier:
                                       Extracellular   0.109
                                       PlasmaMembrane  0.047
                                       Cytoplasmic     0.334
                                       Cytoskeletal    0.018
                                       ER              0.010
                                       Golgi           0.006
```

Table S1. Subcellular localization of proteins retrieved for TriTypDB database showing >80% homology among *L. infantum, L. major* and *L. braziliensis*.

```
                                       Lysosomal              0.008
                                       Mitochondrial  4.021 *
                                       Chloroplast    0.181
                                       Peroxisomal    0.050
                                       Vacuole        0.007
                                       Nuclear        0.209


**********************************************************************
************
SeqID: li|LinJ_29_2480_mRNA|
       CELLO prediction:
                       (predictor     location       reliable-index)
                       Composition    Nuclear        0.401
                       Di-peptide     Mitochondrial  0.502
                       part-Comp.     Nuclear        0.375
                       chemo-typy     Mitochondrial  0.570
                       Neighboring    Nuclear        0.311

       Combined SVM classifier:
                                       Extracellular  0.414
                                       PlasmaMembrane 0.109
                                       Cytoplasmic    0.593
                                       Cytoskeletal   0.015
                                       ER             0.019
                                       Golgi          0.011
                                       Lysosomal              0.013
                                       Mitochondrial  1.696 *
                                       Chloroplast    0.328
                                       Peroxisomal    0.026
                                       Vacuole        0.016
                                       Nuclear        1.760 *


**********************************************************************
************
SeqID: li|LinJ_32_4050_mRNA|
       CELLO prediction:
                       (predictor     location       reliable-index)
                       Composition    Mitochondrial  0.380
                       Di-peptide     Cytoplasmic    0.721
                       part-Comp.     Mitochondrial  0.419
                       chemo-typy     Nuclear        0.377
                       Neighboring    Mitochondrial  0.558

       Combined SVM classifier:
                                       Extracellular  0.045
                                       PlasmaMembrane 0.025
                                       Cytoplasmic    1.850 *
                                       Cytoskeletal   0.010
                                       ER             0.016
                                       Golgi          0.008
                                       Lysosomal              0.011
                                       Mitochondrial  1.666 *
                                       Chloroplast    0.134
                                       Peroxisomal    0.086
                                       Vacuole        0.006
                                       Nuclear        1.142
```

Table S1. Subcellular localization of proteins retrieved for TriTypDB database showing >80% homology among *L. infantum, L. major* and *L. braziliensis*.

```
*******************************************************************
************
SeqID: li|LinJ_25_0260_mRNA|
       CELLO prediction:
                       (predictor      location        reliable-index)
                       Composition     Extracellular   0.399
                       Di-peptide      Cytoplasmic     0.612
                       part-Comp.      Cytoplasmic     0.510
                       chemo-typy      Cytoplasmic     0.531
                       Neighboring     Cytoplasmic     0.507

       Combined SVM classifier:
                                       Extracellular   0.479
                                       PlasmaMembrane  0.045
                                       Cytoplasmic     2.547 *
                                       Cytoskeletal    0.023
                                       ER              0.037
                                       Golgi           0.016
                                       Lysosomal               0.011
                                       Mitochondrial   0.794
                                       Chloroplast     0.197
                                       Peroxisomal     0.213
                                       Vacuole         0.006
                                       Nuclear         0.632


*******************************************************************
************
SeqID: li|LinJ_34_0420_mRNA|
       CELLO prediction:
                       (predictor      location        reliable-index)
                       Composition     Mitochondrial   0.247
                       Di-peptide      Mitochondrial   0.448
                       part-Comp.      Mitochondrial   0.499
                       chemo-typy      Extracellular   0.316
                       Neighboring     Mitochondrial   0.468

       Combined SVM classifier:
                                       Extracellular   0.541
                                       PlasmaMembrane  0.117
                                       Cytoplasmic     0.712
                                       Cytoskeletal    0.164
                                       ER              0.065
                                       Golgi           0.042
                                       Lysosomal               0.029
                                       Mitochondrial   1.943 *
                                       Chloroplast     0.631
                                       Peroxisomal     0.087
                                       Vacuole         0.042
                                       Nuclear         0.626


*******************************************************************
************
SeqID: li|LinJ_04_0680_mRNA|
       CELLO prediction:
                       (predictor      location        reliable-index)
                       Composition     Nuclear         0.516
                       Di-peptide      Nuclear         0.288
                       part-Comp.      Nuclear         0.409
                       chemo-typy      Mitochondrial   0.467
```

Table S1. Subcellular localization of proteins retrieved for TriTypDB database showing >80% homology among *L. infantum, L. major* and *L. braziliensis*.

```
                       Neighboring    Nuclear        0.781

        Combined SVM classifier:
                                      Extracellular  0.333
                                      PlasmaMembrane 0.088
                                      Cytoplasmic    0.896
                                      Cytoskeletal   0.022
                                      ER             0.017
                                      Golgi          0.013
                                      Lysosomal              0.011
                                      Mitochondrial  1.165
                                      Chloroplast    0.241
                                      Peroxisomal    0.058
                                      Vacuole        0.008
                                      Nuclear        2.148 *


**********************************************************************
************
SeqID: li|LinJ_28_0260_mRNA|
        CELLO prediction:
                       (predictor     location       reliable-index)
                       Composition    Mitochondrial  0.832
                       Di-peptide     Mitochondrial  0.758
                       part-Comp.     Mitochondrial  0.364
                       chemo-typy     Cytoplasmic    0.440
                       Neighboring    Mitochondrial  0.876

        Combined SVM classifier:
                                      Extracellular  0.215
                                      PlasmaMembrane 0.048
                                      Cytoplasmic    0.936
                                      Cytoskeletal   0.015
                                      ER             0.029
                                      Golgi          0.014
                                      Lysosomal              0.011
                                      Mitochondrial  2.932 *
                                      Chloroplast    0.386
                                      Peroxisomal    0.243
                                      Vacuole        0.021
                                      Nuclear        0.148


**********************************************************************
************
SeqID: li|LinJ_06_1210_mRNA|
        CELLO prediction:
                       (predictor     location       reliable-index)
                       Composition    Nuclear        0.490
                       Di-peptide     Cytoplasmic    0.333
                       part-Comp.     Nuclear        0.918
                       chemo-typy     Cytoplasmic    0.388
                       Neighboring    Nuclear        0.398

        Combined SVM classifier:
                                      Extracellular  0.290
                                      PlasmaMembrane 0.035
                                      Cytoplasmic    1.176
                                      Cytoskeletal   0.023
                                      ER             0.053
                                      Golgi          0.017
```

Table S1. Subcellular localization of proteins retrieved for TriTypDB database showing >80% homology among *L. infantum, L. major* and *L. braziliensis*.

```
                                      Lysosomal              0.036
                                      Mitochondrial   0.734
                                      Chloroplast     0.344
                                      Peroxisomal     0.179
                                      Vacuole         0.032
                                      Nuclear         2.082 *


**********************************************************************
************
SeqID: li|LinJ_24_2300_mRNA|
        CELLO prediction:
                       (predictor     location        reliable-index)
                       Composition    Mitochondrial   0.416
                       Di-peptide     Chloroplast     0.403
                       part-Comp.     Mitochondrial   0.448
                       chemo-typy     Extracellular   0.535
                       Neighboring    Cytoplasmic     0.448

        Combined SVM classifier:
                                      Extracellular   0.631
                                      PlasmaMembrane  0.027
                                      Cytoplasmic     1.379 *
                                      Cytoskeletal    0.014
                                      ER              0.023
                                      Golgi           0.014
                                      Lysosomal              0.013
                                      Mitochondrial   1.401 *
                                      Chloroplast     0.901
                                      Peroxisomal     0.122
                                      Vacuole         0.017
                                      Nuclear         0.457


**********************************************************************
************
SeqID: li|LinJ_16_0150_mRNA|
        CELLO prediction:
                       (predictor     location        reliable-index)
                       Composition    Nuclear         0.770
                       Di-peptide     Cytoplasmic     0.515
                       part-Comp.     Nuclear         0.620
                       chemo-typy     Chloroplast     0.388
                       Neighboring    Cytoplasmic     0.302

        Combined SVM classifier:
                                      Extracellular   0.060
                                      PlasmaMembrane  0.029
                                      Cytoplasmic     1.063
                                      Cytoskeletal    0.030
                                      ER              0.052
                                      Golgi           0.016
                                      Lysosomal              0.007
                                      Mitochondrial   0.367
                                      Chloroplast     0.847
                                      Peroxisomal     0.052
                                      Vacuole         0.051
                                      Nuclear         2.427 *
```

Table S1. Subcellular localization of proteins retrieved for TriTypDB database showing >80% homology among *L. infantum, L. major* and *L. braziliensis*.

```
**********************************************************************
************
SeqID: li|LinJ_35_1030_mRNA|
        CELLO prediction:
                        (predictor      location        reliable-index)
                        Composition     Cytoplasmic     0.423
                        Di-peptide      Cytoplasmic     0.562
                        part-Comp.      Cytoplasmic     0.559
                        chemo-typy      Cytoplasmic     0.493
                        Neighboring     Mitochondrial   0.504

        Combined SVM classifier:
                                        Extracellular   0.056
                                        PlasmaMembrane  0.083
                                        Cytoplasmic     2.390 *
                                        Cytoskeletal    0.014
                                        ER              0.043
                                        Golgi           0.008
                                        Lysosomal               0.036
                                        Mitochondrial   0.995
                                        Chloroplast     0.091
                                        Peroxisomal     0.078
                                        Vacuole         0.045
                                        Nuclear         1.160


**********************************************************************
************
SeqID: li|LinJ_25_0990_mRNA|
        CELLO prediction:
                        (predictor      location        reliable-index)
                        Composition     Nuclear         0.420
                        Di-peptide      PlasmaMembrane  0.194
                        part-Comp.      Nuclear         0.414
                        chemo-typy      Cytoplasmic     0.469
                        Neighboring     Nuclear         0.313

        Combined SVM classifier:
                                        Extracellular   0.305
                                        PlasmaMembrane  0.550
                                        Cytoplasmic     1.461 *
                                        Cytoskeletal    0.014
                                        ER              0.067
                                        Golgi           0.037
                                        Lysosomal               0.132
                                        Mitochondrial   0.416
                                        Chloroplast     0.235
                                        Peroxisomal     0.105
                                        Vacuole         0.094
                                        Nuclear         1.583 *


**********************************************************************
************
SeqID: li|LinJ_36_3520_mRNA|
        CELLO prediction:
                        (predictor      location        reliable-index)
                        Composition     Nuclear         0.857
                        Di-peptide      Nuclear         0.493
                        part-Comp.      Nuclear         0.610
                        chemo-typy      Cytoplasmic     0.383
```

Table S1. Subcellular localization of proteins retrieved for TriTypDB database showing >80% homology among *L. infantum, L. major* and *L. braziliensis*.

```
                       Neighboring    Cytoplasmic    0.506

        Combined SVM classifier:
                                      Extracellular  0.147
                                      PlasmaMembrane 0.049
                                      Cytoplasmic    1.382
                                      Cytoskeletal   0.041
                                      ER             0.024
                                      Golgi          0.049
                                      Lysosomal              0.009
                                      Mitochondrial  0.251
                                      Chloroplast    0.303
                                      Peroxisomal    0.142
                                      Vacuole        0.025
                                      Nuclear        2.579 *


*********************************************************************
************
SeqID: li|LinJ_01_0790_mRNA|
        CELLO prediction:
                       (predictor     location       reliable-index)
                       Composition    Cytoplasmic    0.282
                       Di-peptide     Chloroplast    0.720
                       part-Comp.     Nuclear        0.426
                       chemo-typy     Cytoplasmic    0.818
                       Neighboring    Nuclear        0.288

        Combined SVM classifier:
                                      Extracellular  0.030
                                      PlasmaMembrane 0.192
                                      Cytoplasmic    1.805 *
                                      Cytoskeletal   0.029
                                      ER             0.054
                                      Golgi          0.039
                                      Lysosomal              0.010
                                      Mitochondrial  0.676
                                      Chloroplast    1.031
                                      Peroxisomal    0.110
                                      Vacuole        0.023
                                      Nuclear        1.003


*********************************************************************
************
SeqID: li|LinJ_22_0140_mRNA|
        CELLO prediction:
                       (predictor     location       reliable-index)
                       Composition    Extracellular  0.697
                       Di-peptide     Mitochondrial  0.492
                       part-Comp.     Nuclear        0.817
                       chemo-typy     Nuclear        0.361
                       Neighboring    Nuclear        0.763

        Combined SVM classifier:
                                      Extracellular  0.815
                                      PlasmaMembrane 0.027
                                      Cytoplasmic    0.757
                                      Cytoskeletal   0.015
                                      ER             0.015
                                      Golgi          0.009
```

Table S1. Subcellular localization of proteins retrieved for TriTypDB database showing >80% homology among *L. infantum, L. major* and *L. braziliensis*.

```
                                       Lysosomal              0.012
                                       Mitochondrial   0.889
                                       Chloroplast     0.166
                                       Peroxisomal     0.011
                                       Vacuole         0.008
                                       Nuclear         2.275 *


**********************************************************************
************
SeqID: li|LinJ_09_1190_mRNA|
        CELLO prediction:
                       (predictor      location        reliable-index)
                       Composition     PlasmaMembrane 0.386
                       Di-peptide      Mitochondrial   0.517
                       part-Comp.      PlasmaMembrane 0.561
                       chemo-typy      PlasmaMembrane 0.693
                       Neighboring     Mitochondrial   0.358

        Combined SVM classifier:
                                       Extracellular   0.142
                                       PlasmaMembrane 2.258 *
                                       Cytoplasmic     0.262
                                       Cytoskeletal    0.013
                                       ER              0.036
                                       Golgi           0.013
                                       Lysosomal              0.033
                                       Mitochondrial   1.386
                                       Chloroplast     0.565
                                       Peroxisomal     0.107
                                       Vacuole         0.051
                                       Nuclear         0.133


**********************************************************************
************
SeqID: li|LinJ_34_4150_mRNA|
        CELLO prediction:
                       (predictor      location        reliable-index)
                       Composition     Mitochondrial   0.319
                       Di-peptide      Mitochondrial   0.461
                       part-Comp.      Mitochondrial   0.445
                       chemo-typy      Mitochondrial   0.463
                       Neighboring     Mitochondrial   0.447

        Combined SVM classifier:
                                       Extracellular   0.269
                                       PlasmaMembrane 0.186
                                       Cytoplasmic     0.696
                                       Cytoskeletal    0.021
                                       ER              0.020
                                       Golgi           0.012
                                       Lysosomal              0.029
                                       Mitochondrial   2.135 *
                                       Chloroplast     0.651
                                       Peroxisomal     0.080
                                       Vacuole         0.021
                                       Nuclear         0.881
```

Table S1. Subcellular localization of proteins retrieved for TriTypDB database showing >80% homology among *L. infantum, L. major* and *L. braziliensis*.

```
**********************************************************************
************
SeqID: li|LinJ_21_0930_mRNA|
        CELLO prediction:
                        (predictor      location        reliable-index)
                        Composition     Nuclear         0.528
                        Di-peptide      Mitochondrial   0.367
                        part-Comp.      Nuclear         0.799
                        chemo-typy      Nuclear         0.782
                        Neighboring     Extracellular   0.353

        Combined SVM classifier:
                                        Extracellular   0.868
                                        PlasmaMembrane  0.012
                                        Cytoplasmic     0.352
                                        Cytoskeletal    0.011
                                        ER              0.013
                                        Golgi           0.006
                                        Lysosomal               0.038
                                        Mitochondrial   0.861
                                        Chloroplast     0.034
                                        Peroxisomal     0.054
                                        Vacuole         0.037
                                        Nuclear         2.713 *


**********************************************************************
************
SeqID: li|LinJ_13_1120_mRNA|
        CELLO prediction:
                        (predictor      location        reliable-index)
                        Composition     Mitochondrial   0.611
                        Di-peptide      Mitochondrial   0.540
                        part-Comp.      Cytoplasmic     0.545
                        chemo-typy      Mitochondrial   0.676
                        Neighboring     Cytoplasmic     0.496

        Combined SVM classifier:
                                        Extracellular   0.039
                                        PlasmaMembrane  0.011
                                        Cytoplasmic     1.891 *
                                        Cytoskeletal    0.065
                                        ER              0.012
                                        Golgi           0.007
                                        Lysosomal               0.006
                                        Mitochondrial   2.489 *
                                        Chloroplast     0.130
                                        Peroxisomal     0.151
                                        Vacuole         0.007
                                        Nuclear         0.193


**********************************************************************
************
SeqID: li|LinJ_36_1000_mRNA|
        CELLO prediction:
                        (predictor      location        reliable-index)
                        Composition     Nuclear         0.332
                        Di-peptide      Mitochondrial   0.479
                        part-Comp.      Cytoplasmic     0.485
                        chemo-typy      Cytoplasmic     0.543
```

Table S1. Subcellular localization of proteins retrieved for TriTypDB database showing >80% homology among *L. infantum, L. major* and *L. braziliensis*.

```
                       Neighboring     Mitochondrial  0.400

        Combined SVM classifier:
                                       Extracellular  0.086
                                       PlasmaMembrane 0.020
                                       Cytoplasmic    1.868 *
                                       Cytoskeletal   0.038
                                       ER             0.016
                                       Golgi          0.009
                                       Lysosomal              0.008
                                       Mitochondrial  1.679 *
                                       Chloroplast    0.246
                                       Peroxisomal    0.094
                                       Vacuole        0.008
                                       Nuclear        0.927


************************************************************************
************
SeqID: li|LinJ_21_1320_mRNA|
        CELLO prediction:
                       (predictor      location       reliable-index)
                       Composition     Cytoplasmic    0.434
                       Di-peptide      Cytoplasmic    0.455
                       part-Comp.      Nuclear        0.449
                       chemo-typy      Cytoplasmic    0.672
                       Neighboring     Mitochondrial  0.403

        Combined SVM classifier:
                                       Extracellular  0.403
                                       PlasmaMembrane 0.143
                                       Cytoplasmic    2.199 *
                                       Cytoskeletal   0.010
                                       ER             0.038
                                       Golgi          0.028
                                       Lysosomal              0.023
                                       Mitochondrial  0.779
                                       Chloroplast    0.110
                                       Peroxisomal    0.108
                                       Vacuole        0.018
                                       Nuclear        1.141


************************************************************************
************
SeqID: li|LinJ_28_2280_mRNA|
        CELLO prediction:
                       (predictor      location       reliable-index)
                       Composition     Cytoplasmic    0.300
                       Di-peptide      Mitochondrial  0.354
                       part-Comp.      Cytoplasmic    0.285
                       chemo-typy      Nuclear        0.279
                       Neighboring     Cytoplasmic    0.372

        Combined SVM classifier:
                                       Extracellular  0.485
                                       PlasmaMembrane 0.179
                                       Cytoplasmic    1.390 *
                                       Cytoskeletal   0.017
                                       ER             0.044
                                       Golgi          0.021
```

Table S1. Subcellular localization of proteins retrieved for TriTypDB database showing >80% homology among *L. infantum, L. major* and *L. braziliensis*.

```
                                        Lysosomal              0.041
                                        Mitochondrial   1.290 *
                                        Chloroplast     0.788
                                        Peroxisomal     0.097
                                        Vacuole         0.038
                                        Nuclear         0.608


************************************************************************
************
SeqID: li|LinJ_21_2190_mRNA|
        CELLO prediction:
                        (predictor      location        reliable-index)
                        Composition     Cytoplasmic     0.328
                        Di-peptide      Mitochondrial   0.514
                        part-Comp.      Nuclear         0.356
                        chemo-typy      Nuclear         0.533
                        Neighboring     Nuclear         0.285

        Combined SVM classifier:
                                        Extracellular   0.228
                                        PlasmaMembrane  0.075
                                        Cytoplasmic     0.717
                                        Cytoskeletal    0.014
                                        ER              0.014
                                        Golgi           0.008
                                        Lysosomal              0.019
                                        Mitochondrial   1.827 *
                                        Chloroplast     0.309
                                        Peroxisomal     0.065
                                        Vacuole         0.021
                                        Nuclear         1.703 *


************************************************************************
************
SeqID: li|LinJ_28_2940_mRNA|
        CELLO prediction:
                        (predictor      location        reliable-index)
                        Composition     Extracellular   0.598
                        Di-peptide      Extracellular   0.640
                        part-Comp.      Cytoplasmic     0.347
                        chemo-typy      Chloroplast     0.524
                        Neighboring     Cytoplasmic     0.307

        Combined SVM classifier:
                                        Extracellular   1.645 *
                                        PlasmaMembrane  0.219
                                        Cytoplasmic     0.989
                                        Cytoskeletal    0.013
                                        ER              0.026
                                        Golgi           0.047
                                        Lysosomal              0.221
                                        Mitochondrial   0.414
                                        Chloroplast     0.877
                                        Peroxisomal     0.121
                                        Vacuole         0.079
                                        Nuclear         0.348
```

Table S1. Subcellular localization of proteins retrieved for TriTypDB database showing >80% homology among *L. infantum, L. major* and *L. braziliensis.*

```
**********************************************************************
************
SeqID: li|LinJ_14_1350_mRNA|
       CELLO prediction:
                       (predictor      location        reliable-index)
                       Composition     Cytoplasmic     0.662
                       Di-peptide      Cytoplasmic     0.472
                       part-Comp.      Nuclear         0.353
                       chemo-typy      Extracellular   0.363
                       Neighboring     Nuclear         0.440

       Combined SVM classifier:
                                       Extracellular   0.633
                                       PlasmaMembrane  0.014
                                       Cytoplasmic     1.534 *
                                       Cytoskeletal    0.009
                                       ER              0.042
                                       Golgi           0.006
                                       Lysosomal               0.009
                                       Mitochondrial   0.756
                                       Chloroplast     0.190
                                       Peroxisomal     0.040
                                       Vacuole         0.036
                                       Nuclear         1.732 *


**********************************************************************
************
SeqID: li|LinJ_11_0960_mRNA|
       CELLO prediction:
                       (predictor      location        reliable-index)
                       Composition     Nuclear         0.438
                       Di-peptide      Nuclear         0.599
                       part-Comp.      Mitochondrial   0.881
                       chemo-typy      Cytoplasmic     0.600
                       Neighboring     Mitochondrial   0.473

       Combined SVM classifier:
                                       Extracellular   0.024
                                       PlasmaMembrane  0.011
                                       Cytoplasmic     0.882
                                       Cytoskeletal    0.015
                                       ER              0.015
                                       Golgi           0.007
                                       Lysosomal               0.006
                                       Mitochondrial   2.170 *
                                       Chloroplast     0.413
                                       Peroxisomal     0.052
                                       Vacuole         0.007
                                       Nuclear         1.397


**********************************************************************
************
SeqID: li|LinJ_28_2200_mRNA|
       CELLO prediction:
                       (predictor      location        reliable-index)
                       Composition     Nuclear         0.577
                       Di-peptide      Nuclear         0.429
                       part-Comp.      Nuclear         0.877
                       chemo-typy      Cytoplasmic     0.682
```

Table S1. Subcellular localization of proteins retrieved for TriTypDB database showing >80% homology among *L. infantum, L. major* and *L. braziliensis*.

```
                       Neighboring     Cytoplasmic     0.446

        Combined SVM classifier:
                                       Extracellular  0.225
                                       PlasmaMembrane 0.027
                                       Cytoplasmic    1.658
                                       Cytoskeletal   0.030
                                       ER             0.022
                                       Golgi          0.144
                                       Lysosomal              0.008
                                       Mitochondrial  0.156
                                       Chloroplast    0.269
                                       Peroxisomal    0.048
                                       Vacuole        0.010
                                       Nuclear        2.402 *


*********************************************************************
************
SeqID: li|LinJ_34_4050_mRNA|
        CELLO prediction:
                       (predictor      location       reliable-index)
                       Composition     Nuclear        0.688
                       Di-peptide      Cytoplasmic    0.521
                       part-Comp.      Mitochondrial  0.436
                       chemo-typy      Mitochondrial  0.516
                       Neighboring     Cytoplasmic    0.538

        Combined SVM classifier:
                                       Extracellular  0.463
                                       PlasmaMembrane 0.008
                                       Cytoplasmic    1.363 *
                                       Cytoskeletal   0.022
                                       ER             0.042
                                       Golgi          0.007
                                       Lysosomal              0.005
                                       Mitochondrial  1.449 *
                                       Chloroplast    0.024
                                       Peroxisomal    0.043
                                       Vacuole        0.005
                                       Nuclear        1.568 *


*********************************************************************
************
SeqID: li|LinJ_21_1990_mRNA|
        CELLO prediction:
                       (predictor      location       reliable-index)
                       Composition     Nuclear        0.311
                       Di-peptide      Cytoplasmic    0.695
                       part-Comp.      Cytoplasmic    0.645
                       chemo-typy      Cytoplasmic    0.686
                       Neighboring     Cytoplasmic    0.437

        Combined SVM classifier:
                                       Extracellular  0.168
                                       PlasmaMembrane 0.191
                                       Cytoplasmic    2.604 *
                                       Cytoskeletal   0.020
                                       ER             0.058
                                       Golgi          0.167
```

Table S1. Subcellular localization of proteins retrieved for TriTypDB database showing >80% homology among *L. infantum, L. major* and *L. braziliensis*.

```
                                       Lysosomal               0.011
                                       Mitochondrial   0.417
                                       Chloroplast     0.372
                                       Peroxisomal     0.102
                                       Vacuole         0.185
                                       Nuclear         0.705


**********************************************************************
************
SeqID: li|LinJ_34_3620_mRNA|
        CELLO prediction:
                       (predictor      location        reliable-index)
                       Composition     Extracellular   0.365
                       Di-peptide      Mitochondrial   0.510
                       part-Comp.      Mitochondrial   0.855
                       chemo-typy      Mitochondrial   0.495
                       Neighboring     Mitochondrial   0.673

        Combined SVM classifier:
                                       Extracellular   0.486
                                       PlasmaMembrane  0.075
                                       Cytoplasmic     0.205
                                       Cytoskeletal    0.008
                                       ER              0.009
                                       Golgi           0.005
                                       Lysosomal               0.013
                                       Mitochondrial   2.861 *
                                       Chloroplast     0.138
                                       Peroxisomal     0.087
                                       Vacuole         0.025
                                       Nuclear         1.088


**********************************************************************
************
SeqID: li|LinJ_28_0730_mRNA|
        CELLO prediction:
                       (predictor      location        reliable-index)
                       Composition     Cytoplasmic     0.372
                       Di-peptide      Cytoplasmic     0.597
                       part-Comp.      Cytoplasmic     0.862
                       chemo-typy      Cytoplasmic     0.564
                       Neighboring     Cytoplasmic     0.895

        Combined SVM classifier:
                                       Extracellular   0.345
                                       PlasmaMembrane  0.414
                                       Cytoplasmic     3.290 *
                                       Cytoskeletal    0.006
                                       ER              0.010
                                       Golgi           0.049
                                       Lysosomal               0.012
                                       Mitochondrial   0.113
                                       Chloroplast     0.063
                                       Peroxisomal     0.081
                                       Vacuole         0.011
                                       Nuclear         0.606
```

Table S1. Subcellular localization of proteins retrieved for TriTypDB database showing >80% homology among *L. infantum, L. major* and *L. braziliensis.*

```
**********************************************************************
************
SeqID: li|LinJ_23_1470_mRNA|
       CELLO prediction:
                       (predictor      location        reliable-index)
                       Composition     Extracellular   0.631
                       Di-peptide      Mitochondrial   0.753
                       part-Comp.      Nuclear         0.435
                       chemo-typy      Cytoplasmic     0.371
                       Neighboring     Mitochondrial   0.483

       Combined SVM classifier:
                                       Extracellular   0.748
                                       PlasmaMembrane  0.046
                                       Cytoplasmic     0.864
                                       Cytoskeletal    0.026
                                       ER              0.020
                                       Golgi           0.024
                                       Lysosomal               0.009
                                       Mitochondrial   1.852 *
                                       Chloroplast     0.387
                                       Peroxisomal     0.031
                                       Vacuole         0.024
                                       Nuclear         0.968


**********************************************************************
************
SeqID: li|LinJ_35_0070_mRNA|
       CELLO prediction:
                       (predictor      location        reliable-index)
                       Composition     Mitochondrial   0.816
                       Di-peptide      Mitochondrial   0.749
                       part-Comp.      Mitochondrial   0.505
                       chemo-typy      Chloroplast     0.913
                       Neighboring     Chloroplast     0.465

       Combined SVM classifier:
                                       Extracellular   0.028
                                       PlasmaMembrane  0.191
                                       Cytoplasmic     0.282
                                       Cytoskeletal    0.020
                                       ER              0.010
                                       Golgi           0.022
                                       Lysosomal               0.006
                                       Mitochondrial   2.295 *
                                       Chloroplast     1.701 *
                                       Peroxisomal     0.358
                                       Vacuole         0.028
                                       Nuclear         0.057


**********************************************************************
************
SeqID: li|LinJ_19_0390_mRNA|
       CELLO prediction:
                       (predictor      location        reliable-index)
                       Composition     Mitochondrial   0.584
                       Di-peptide      Nuclear         0.670
                       part-Comp.      Mitochondrial   0.932
                       chemo-typy      Nuclear         0.439
```

Table S1. Subcellular localization of proteins retrieved for TriTypDB database showing >80% homology among *L. infantum, L. major* and *L. braziliensis*.

```
                       Neighboring    Nuclear        0.823

       Combined SVM classifier:
                                      Extracellular  0.152
                                      PlasmaMembrane 0.036
                                      Cytoplasmic    0.241
                                      Cytoskeletal   0.013
                                      ER             0.010
                                      Golgi          0.010
                                      Lysosomal             0.005
                                      Mitochondrial  2.068 *
                                      Chloroplast    0.231
                                      Peroxisomal    0.022
                                      Vacuole        0.006
                                      Nuclear        2.206 *


**********************************************************************
************
SeqID: li|LinJ_35_2270_mRNA|
       CELLO prediction:
                       (predictor     location       reliable-index)
                       Composition    Nuclear        0.272
                       Di-peptide     Mitochondrial  0.515
                       part-Comp.     Nuclear        0.592
                       chemo-typy     Nuclear        0.406
                       Neighboring    Extracellular  0.301

       Combined SVM classifier:
                                      Extracellular  0.680
                                      PlasmaMembrane 0.068
                                      Cytoplasmic    1.166 *
                                      Cytoskeletal   0.046
                                      ER             0.029
                                      Golgi          0.016
                                      Lysosomal             0.011
                                      Mitochondrial  0.941
                                      Chloroplast    0.263
                                      Peroxisomal    0.041
                                      Vacuole        0.019
                                      Nuclear        1.721 *


**********************************************************************
************
SeqID: li|LinJ_20_1020_mRNA|
       CELLO prediction:
                       (predictor     location       reliable-index)
                       Composition    Nuclear        0.736
                       Di-peptide     Cytoplasmic    0.257
                       part-Comp.     Mitochondrial  0.560
                       chemo-typy     Cytoplasmic    0.354
                       Neighboring    Mitochondrial  0.396

       Combined SVM classifier:
                                      Extracellular  0.315
                                      PlasmaMembrane 0.198
                                      Cytoplasmic    0.953
                                      Cytoskeletal   0.019
                                      ER             0.022
                                      Golgi          0.024
```

Table S1. Subcellular localization of proteins retrieved for TriTypDB database showing >80% homology among *L. infantum, L. major* and *L. braziliensis*.

```
                                        Lysosomal             0.015
                                        Mitochondrial  1.623 *
                                        Chloroplast    0.267
                                        Peroxisomal    0.073
                                        Vacuole        0.012
                                        Nuclear        1.478 *


************************************************************************
************
SeqID: li|LinJ_32_2850_mRNA|
        CELLO prediction:
                        (predictor      location       reliable-index)
                        Composition     Mitochondrial  0.693
                        Di-peptide      Mitochondrial  0.838
                        part-Comp.      Mitochondrial  0.299
                        chemo-typy      Mitochondrial  0.827
                        Neighboring     Mitochondrial  0.798

        Combined SVM classifier:
                                        Extracellular  0.095
                                        PlasmaMembrane 0.063
                                        Cytoplasmic    0.586
                                        Cytoskeletal   0.027
                                        ER             0.010
                                        Golgi          0.007
                                        Lysosomal             0.008
                                        Mitochondrial  3.454 *
                                        Chloroplast    0.117
                                        Peroxisomal    0.111
                                        Vacuole        0.012
                                        Nuclear        0.508


************************************************************************
************
SeqID: li|LinJ_34_3670_mRNA|
        CELLO prediction:
                        (predictor      location       reliable-index)
                        Composition     Mitochondrial  0.749
                        Di-peptide      Mitochondrial  0.390
                        part-Comp.      Cytoplasmic    0.430
                        chemo-typy      Cytoplasmic    0.313
                        Neighboring     Cytoplasmic    0.311

        Combined SVM classifier:
                                        Extracellular  0.173
                                        PlasmaMembrane 0.072
                                        Cytoplasmic    1.263 *
                                        Cytoskeletal   0.015
                                        ER             0.070
                                        Golgi          0.020
                                        Lysosomal             0.006
                                        Mitochondrial  1.707 *
                                        Chloroplast    0.546
                                        Peroxisomal    0.045
                                        Vacuole        0.020
                                        Nuclear        1.062 *
```

Table S1. Subcellular localization of proteins retrieved for TriTypDB database showing >80% homology among *L. infantum, L. major* and *L. braziliensis*.

```
**********************************************************************
************
SeqID: li|LinJ_33_0860_mRNA|
       CELLO prediction:
                       (predictor     location       reliable-index)
                       Composition    Cytoplasmic    0.518
                       Di-peptide     Cytoplasmic    0.851
                       part-Comp.     Cytoplasmic    0.841
                       chemo-typy     Cytoplasmic    0.691
                       Neighboring    Cytoplasmic    0.814

       Combined SVM classifier:
                                      Extracellular  0.097
                                      PlasmaMembrane 0.180
                                      Cytoplasmic    3.714 *
                                      Cytoskeletal   0.008
                                      ER             0.026
                                      Golgi          0.017
                                      Lysosomal              0.045
                                      Mitochondrial  0.072
                                      Chloroplast    0.104
                                      Peroxisomal    0.031
                                      Vacuole        0.041
                                      Nuclear        0.664


**********************************************************************
************
SeqID: li|LinJ_22_1280_mRNA|
       CELLO prediction:
                       (predictor     location       reliable-index)
                       Composition    Cytoplasmic    0.363
                       Di-peptide     Cytoplasmic    0.616
                       part-Comp.     Chloroplast    0.628
                       chemo-typy     Cytoplasmic    0.499
                       Neighboring    Chloroplast    0.595

       Combined SVM classifier:
                                      Extracellular  0.086
                                      PlasmaMembrane 0.304
                                      Cytoplasmic    1.959 *
                                      Cytoskeletal   0.009
                                      ER             0.038
                                      Golgi          0.022
                                      Lysosomal              0.040
                                      Mitochondrial  0.310
                                      Chloroplast    1.809 *
                                      Peroxisomal    0.056
                                      Vacuole        0.024
                                      Nuclear        0.343


**********************************************************************
************
SeqID: li|LinJ_27_0180_mRNA|
       CELLO prediction:
                       (predictor     location       reliable-index)
                       Composition    Nuclear        0.553
                       Di-peptide     Mitochondrial  0.507
                       part-Comp.     Nuclear        0.411
                       chemo-typy     Mitochondrial  0.515
```

Table S1. Subcellular localization of proteins retrieved for TriTypDB database showing >80% homology among *L. infantum, L. major* and *L. braziliensis*.

```
                       Neighboring     Mitochondrial   0.573

        Combined SVM classifier:
                                       Extracellular   0.123
                                       PlasmaMembrane  0.027
                                       Cytoplasmic     0.555
                                       Cytoskeletal    0.063
                                       ER              0.022
                                       Golgi           0.008
                                       Lysosomal               0.011
                                       Mitochondrial   2.066 *
                                       Chloroplast     0.056
                                       Peroxisomal     0.064
                                       Vacuole         0.015
                                       Nuclear         1.991 *


**********************************************************************
************
SeqID: li|LinJ_03_0090_mRNA|
        CELLO prediction:
                       (predictor      location        reliable-index)
                       Composition     Chloroplast     0.249
                       Di-peptide      Mitochondrial   0.375
                       part-Comp.      Nuclear         0.440
                       chemo-typy      Cytoplasmic     0.380
                       Neighboring     Mitochondrial   0.301

        Combined SVM classifier:
                                       Extracellular   0.586
                                       PlasmaMembrane  0.511
                                       Cytoplasmic     0.826
                                       Cytoskeletal    0.029
                                       ER              0.070
                                       Golgi           0.045
                                       Lysosomal               0.038
                                       Mitochondrial   1.094 *
                                       Chloroplast     0.690
                                       Peroxisomal     0.189
                                       Vacuole         0.073
                                       Nuclear         0.849


**********************************************************************
************
SeqID: li|LinJ_28_0680_mRNA|
        CELLO prediction:
                       (predictor      location        reliable-index)
                       Composition     PlasmaMembrane  0.975
                       Di-peptide      PlasmaMembrane  0.532
                       part-Comp.      PlasmaMembrane  0.393
                       chemo-typy      PlasmaMembrane  0.772
                       Neighboring     PlasmaMembrane  0.342

        Combined SVM classifier:
                                       Extracellular   0.591
                                       PlasmaMembrane  3.015 *
                                       Cytoplasmic     0.308
                                       Cytoskeletal    0.010
                                       ER              0.017
                                       Golgi           0.014
```

Table S1. Subcellular localization of proteins retrieved for TriTypDB database showing >80% homology among *L. infantum, L. major* and *L. braziliensis*.

```
                                    Lysosomal              0.015
                                    Mitochondrial   0.342
                                    Chloroplast     0.369
                                    Peroxisomal     0.032
                                    Vacuole         0.008
                                    Nuclear         0.279


*********************************************************************
************
SeqID: li|LinJ_26_1610_mRNA|
       CELLO prediction:
                      (predictor      location        reliable-index)
                      Composition     Cytoplasmic     0.297
                      Di-peptide      Chloroplast     0.428
                      part-Comp.      Nuclear         0.938
                      chemo-typy      Nuclear         0.481
                      Neighboring     Nuclear         0.668

       Combined SVM classifier:
                                    Extracellular   0.061
                                    PlasmaMembrane  0.040
                                    Cytoplasmic     1.110
                                    Cytoskeletal    0.020
                                    ER              0.013
                                    Golgi           0.012
                                    Lysosomal              0.010
                                    Mitochondrial   0.489
                                    Chloroplast     0.688
                                    Peroxisomal     0.026
                                    Vacuole         0.018
                                    Nuclear         2.513  *


*********************************************************************
************
SeqID: li|LinJ_36_3070_mRNA|
       CELLO prediction:
                      (predictor      location        reliable-index)
                      Composition     Extracellular   0.945
                      Di-peptide      Extracellular   0.698
                      part-Comp.      Extracellular   0.343
                      chemo-typy      Nuclear         0.366
                      Neighboring     Extracellular   0.714

       Combined SVM classifier:
                                    Extracellular   2.728  *
                                    PlasmaMembrane  0.113
                                    Cytoplasmic     0.485
                                    Cytoskeletal    0.015
                                    ER              0.017
                                    Golgi           0.018
                                    Lysosomal              0.034
                                    Mitochondrial   0.404
                                    Chloroplast     0.253
                                    Peroxisomal     0.024
                                    Vacuole         0.015
                                    Nuclear         0.892
```

Table S1. Subcellular localization of proteins retrieved for TriTypDB database showing >80% homology among *L. infantum, L. major* and *L. braziliensis.*

```
**********************************************************************
************
SeqID: li|LinJ_15_1010_mRNA|
       CELLO prediction:
                       (predictor      location        reliable-index)
                       Composition     Mitochondrial   0.401
                       Di-peptide      Mitochondrial   0.599
                       part-Comp.      Mitochondrial   0.478
                       chemo-typy      Nuclear         0.432
                       Neighboring     Cytoplasmic     0.534

       Combined SVM classifier:
                                       Extracellular   0.032
                                       PlasmaMembrane  0.089
                                       Cytoplasmic     1.509 *
                                       Cytoskeletal    0.014
                                       ER              0.015
                                       Golgi           0.014
                                       Lysosomal               0.013
                                       Mitochondrial   1.817 *
                                       Chloroplast     0.352
                                       Peroxisomal     0.154
                                       Vacuole         0.014
                                       Nuclear         0.977


**********************************************************************
************
SeqID: li|LinJ_30_0710_mRNA|
       CELLO prediction:
                       (predictor      location        reliable-index)
                       Composition     Cytoplasmic     0.343
                       Di-peptide      Mitochondrial   0.441
                       part-Comp.      Nuclear         0.710
                       chemo-typy      Mitochondrial   0.432
                       Neighboring     Mitochondrial   0.396

       Combined SVM classifier:
                                       Extracellular   0.206
                                       PlasmaMembrane  0.061
                                       Cytoplasmic     0.980
                                       Cytoskeletal    0.016
                                       ER              0.017
                                       Golgi           0.011
                                       Lysosomal               0.011
                                       Mitochondrial   1.504 *
                                       Chloroplast     0.168
                                       Peroxisomal     0.028
                                       Vacuole         0.014
                                       Nuclear         1.983 *


**********************************************************************
************
SeqID: li|LinJ_25_1050_mRNA|
       CELLO prediction:
                       (predictor      location        reliable-index)
                       Composition     PlasmaMembrane  0.643
                       Di-peptide      PlasmaMembrane  0.569
                       part-Comp.      PlasmaMembrane  0.402
                       chemo-typy      PlasmaMembrane  0.506
```

Table S1. Subcellular localization of proteins retrieved for TriTypDB database showing >80% homology among *L. infantum, L. major* and *L. braziliensis*.

```
                       Neighboring    Nuclear        0.243

        Combined SVM classifier:
                                      Extracellular  0.517
                                      PlasmaMembrane 2.210 *
                                      Cytoplasmic    0.426
                                      Cytoskeletal   0.017
                                      ER             0.024
                                      Golgi          0.018
                                      Lysosomal              0.023
                                      Mitochondrial  0.568
                                      Chloroplast    0.360
                                      Peroxisomal    0.040
                                      Vacuole        0.023
                                      Nuclear        0.776


************************************************************************
************
SeqID: li|LinJ_05_0030_mRNA|
        CELLO prediction:
                       (predictor     location       reliable-index)
                       Composition    Lysosomal              0.273
                       Di-peptide     Extracellular  0.380
                       part-Comp.     Lysosomal              0.375
                       chemo-typy     Cytoplasmic    0.362
                       Neighboring    Lysosomal              0.219

        Combined SVM classifier:
                                      Extracellular  0.911
                                      PlasmaMembrane 0.095
                                      Cytoplasmic    0.864
                                      Cytoskeletal   0.013
                                      ER             0.247
                                      Golgi          0.040
                                      Lysosomal              0.892
                                      Mitochondrial  0.942 *
                                      Chloroplast    0.595
                                      Peroxisomal    0.121
                                      Vacuole        0.072
                                      Nuclear        0.208


************************************************************************
************
SeqID: li|LinJ_18_1180_mRNA|
        CELLO prediction:
                       (predictor     location       reliable-index)
                       Composition    Mitochondrial  0.296
                       Di-peptide     Extracellular  0.566
                       part-Comp.     PlasmaMembrane 0.465
                       chemo-typy     Extracellular  0.730
                       Neighboring    Mitochondrial  0.572

        Combined SVM classifier:
                                      Extracellular  1.513 *
                                      PlasmaMembrane 0.660
                                      Cytoplasmic    1.036 *
                                      Cytoskeletal   0.047
                                      ER             0.051
                                      Golgi          0.019
```

Table S1. Subcellular localization of proteins retrieved for TriTypDB database showing >80% homology among *L. infantum, L. major* and *L. braziliensis*.

```
                                       Lysosomal              0.055
                                       Mitochondrial  0.966
                                       Chloroplast    0.234
                                       Peroxisomal    0.114
                                       Vacuole        0.083
                                       Nuclear        0.222


**********************************************************************
************
SeqID: li|LinJ_22_0340_mRNA|
        CELLO prediction:
                        (predictor      location        reliable-index)
                        Composition     Mitochondrial   0.636
                        Di-peptide      Mitochondrial   0.711
                        part-Comp.      Cytoplasmic     0.428
                        chemo-typy      Nuclear         0.849
                        Neighboring     Mitochondrial   0.605

        Combined SVM classifier:
                                        Extracellular   0.058
                                        PlasmaMembrane  0.025
                                        Cytoplasmic     1.210
                                        Cytoskeletal    0.019
                                        ER              0.019
                                        Golgi           0.009
                                        Lysosomal               0.009
                                        Mitochondrial   2.167 *
                                        Chloroplast     0.115
                                        Peroxisomal     0.068
                                        Vacuole         0.007
                                        Nuclear         1.296


**********************************************************************
************
SeqID: li|LinJ_13_1530_mRNA|
        CELLO prediction:
                        (predictor      location        reliable-index)
                        Composition     Cytoplasmic     0.380
                        Di-peptide      Cytoplasmic     0.819
                        part-Comp.      Nuclear         0.643
                        chemo-typy      Cytoplasmic     0.691
                        Neighboring     Cytoplasmic     0.636

        Combined SVM classifier:
                                        Extracellular   0.193
                                        PlasmaMembrane  0.021
                                        Cytoplasmic     2.797 *
                                        Cytoskeletal    0.012
                                        ER              0.151
                                        Golgi           0.023
                                        Lysosomal               0.012
                                        Mitochondrial   0.404
                                        Chloroplast     0.179
                                        Peroxisomal     0.045
                                        Vacuole         0.036
                                        Nuclear         1.126
```

Table S1. Subcellular localization of proteins retrieved for TriTypDB database showing >80% homology among *L. infantum, L. major* and *L. braziliensis*.

```
**********************************************************************
************
SeqID: li|LinJ_35_3810_mRNA|
        CELLO prediction:
                        (predictor      location        reliable-index)
                        Composition     Mitochondrial   0.519
                        Di-peptide      Nuclear         0.820
                        part-Comp.      Mitochondrial   0.319
                        chemo-typy      Nuclear         0.991
                        Neighboring     Mitochondrial   0.757

        Combined SVM classifier:
                                        Extracellular   0.051
                                        PlasmaMembrane  0.013
                                        Cytoplasmic     0.755
                                        Cytoskeletal    0.010
                                        ER              0.013
                                        Golgi           0.007
                                        Lysosomal               0.016
                                        Mitochondrial   1.691 *
                                        Chloroplast     0.057
                                        Peroxisomal     0.080
                                        Vacuole         0.013
                                        Nuclear         2.293 *


**********************************************************************
************
SeqID: li|LinJ_25_0670_mRNA|
        CELLO prediction:
                        (predictor      location        reliable-index)
                        Composition     Mitochondrial   0.287
                        Di-peptide      Mitochondrial   0.617
                        part-Comp.      Mitochondrial   0.385
                        chemo-typy      Mitochondrial   0.571
                        Neighboring     Extracellular   0.844

        Combined SVM classifier:
                                        Extracellular   1.187
                                        PlasmaMembrane  0.402
                                        Cytoplasmic     0.126
                                        Cytoskeletal    0.011
                                        ER              0.054
                                        Golgi           0.009
                                        Lysosomal               0.079
                                        Mitochondrial   1.921 *
                                        Chloroplast     0.471
                                        Peroxisomal     0.068
                                        Vacuole         0.036
                                        Nuclear         0.637


**********************************************************************
************
SeqID: li|LinJ_22_0004_mRNA|
        CELLO prediction:
                        (predictor      location        reliable-index)
                        Composition     Cytoplasmic     0.578
                        Di-peptide      Cytoplasmic     0.851
                        part-Comp.      Cytoplasmic     0.668
                        chemo-typy      Nuclear         0.685
```

Table S1. Subcellular localization of proteins retrieved for TriTypDB database showing >80% homology among *L. infantum, L. major* and *L. braziliensis*.

```
                       Neighboring     Cytoplasmic     0.538

        Combined SVM classifier:
                                       Extracellular  0.035
                                       PlasmaMembrane 0.016
                                       Cytoplasmic    2.909 *
                                       Cytoskeletal   0.017
                                       ER             0.023
                                       Golgi          0.010
                                       Lysosomal              0.006
                                       Mitochondrial  0.364
                                       Chloroplast    0.126
                                       Peroxisomal    0.037
                                       Vacuole        0.007
                                       Nuclear        1.452


**********************************************************************
************
SeqID: li|LinJ_30_2000_mRNA|
        CELLO prediction:
                       (predictor      location        reliable-index)
                       Composition     Cytoplasmic     0.378
                       Di-peptide      Cytoplasmic     0.624
                       part-Comp.      Nuclear         0.407
                       chemo-typy      Cytoplasmic     0.479
                       Neighboring     Cytoplasmic     0.466

        Combined SVM classifier:
                                       Extracellular  0.431
                                       PlasmaMembrane 0.063
                                       Cytoplasmic    2.150 *
                                       Cytoskeletal   0.274
                                       ER             0.020
                                       Golgi          0.070
                                       Lysosomal              0.010
                                       Mitochondrial  0.435
                                       Chloroplast    0.378
                                       Peroxisomal    0.038
                                       Vacuole        0.020
                                       Nuclear        1.111


**********************************************************************
************
SeqID: li|LinJ_22_0440_mRNA|
        CELLO prediction:
                       (predictor      location        reliable-index)
                       Composition     Cytoplasmic     0.428
                       Di-peptide      Cytoplasmic     0.831
                       part-Comp.      Cytoplasmic     0.639
                       chemo-typy      Cytoplasmic     0.889
                       Neighboring     Cytoplasmic     0.536

        Combined SVM classifier:
                                       Extracellular  0.015
                                       PlasmaMembrane 0.011
                                       Cytoplasmic    3.324 *
                                       Cytoskeletal   0.020
                                       ER             0.014
                                       Golgi          0.012
```

Table S1. Subcellular localization of proteins retrieved for TriTypDB database showing >80% homology among *L. infantum, L. major* and *L. braziliensis*.

```
                                       Lysosomal              0.004
                                       Mitochondrial   0.626
                                       Chloroplast     0.176
                                       Peroxisomal     0.030
                                       Vacuole         0.005
                                       Nuclear         0.763


**********************************************************************
************
SeqID: li|LinJ_34_2160_mRNA|
        CELLO prediction:
                       (predictor      location        reliable-index)
                       Composition     Cytoplasmic     0.463
                       Di-peptide      Nuclear         0.937
                       part-Comp.      Nuclear         0.390
                       chemo-typy      Cytoplasmic     0.693
                       Neighboring     Nuclear         0.285

        Combined SVM classifier:
                                       Extracellular   0.142
                                       PlasmaMembrane  0.031
                                       Cytoplasmic     1.758 *
                                       Cytoskeletal    0.070
                                       ER              0.053
                                       Golgi           0.038
                                       Lysosomal              0.007
                                       Mitochondrial   0.449
                                       Chloroplast     0.377
                                       Peroxisomal     0.032
                                       Vacuole         0.052
                                       Nuclear         1.991 *


**********************************************************************
************
SeqID: li|LinJ_06_0010_mRNA|
        CELLO prediction:
                       (predictor      location        reliable-index)
                       Composition     Cytoplasmic     0.557
                       Di-peptide      Nuclear         0.808
                       part-Comp.      Nuclear         0.567
                       chemo-typy      Nuclear         0.793
                       Neighboring     Nuclear         0.511

        Combined SVM classifier:
                                       Extracellular   0.113
                                       PlasmaMembrane  0.036
                                       Cytoplasmic     0.889
                                       Cytoskeletal    0.013
                                       ER              0.013
                                       Golgi           0.008
                                       Lysosomal              0.008
                                       Mitochondrial   0.798
                                       Chloroplast     0.263
                                       Peroxisomal     0.032
                                       Vacuole         0.015
                                       Nuclear         2.811 *
```

Table S1. Subcellular localization of proteins retrieved for TriTypDB database showing >80% homology among *L. infantum, L. major* and *L. braziliensis*.

```
**********************************************************************
************
SeqID: li|LinJ_21_1790_mRNA|
        CELLO prediction:
                        (predictor      location        reliable-index)
                        Composition     Mitochondrial   0.545
                        Di-peptide      Mitochondrial   0.540
                        part-Comp.      Mitochondrial   0.613
                        chemo-typy      Mitochondrial   0.784
                        Neighboring     Mitochondrial   0.445

        Combined SVM classifier:
                                        Extracellular   0.084
                                        PlasmaMembrane  0.032
                                        Cytoplasmic     0.907
                                        Cytoskeletal    0.010
                                        ER              0.017
                                        Golgi           0.008
                                        Lysosomal               0.008
                                        Mitochondrial   2.927 *
                                        Chloroplast     0.128
                                        Peroxisomal     0.056
                                        Vacuole         0.008
                                        Nuclear         0.814


**********************************************************************
************
SeqID: li|LinJ_35_3720_mRNA|
        CELLO prediction:
                        (predictor      location        reliable-index)
                        Composition     Mitochondrial   0.588
                        Di-peptide      Mitochondrial   0.713
                        part-Comp.      Cytoplasmic     0.456
                        chemo-typy      Cytoplasmic     0.587
                        Neighboring     Mitochondrial   0.652

        Combined SVM classifier:
                                        Extracellular   0.281
                                        PlasmaMembrane  0.157
                                        Cytoplasmic     1.651 *
                                        Cytoskeletal    0.018
                                        ER              0.037
                                        Golgi           0.010
                                        Lysosomal               0.101
                                        Mitochondrial   2.015 *
                                        Chloroplast     0.147
                                        Peroxisomal     0.075
                                        Vacuole         0.009
                                        Nuclear         0.498


**********************************************************************
************
SeqID: li|LinJ_16_1390_mRNA|
        CELLO prediction:
                        (predictor      location        reliable-index)
                        Composition     Mitochondrial   0.537
                        Di-peptide      Mitochondrial   0.499
                        part-Comp.      Mitochondrial   0.827
                        chemo-typy      Mitochondrial   0.472
```

Table S1. Subcellular localization of proteins retrieved for TriTypDB database showing >80% homology among *L. infantum, L. major* and *L. braziliensis*.

```
                       Neighboring    Mitochondrial  0.737

        Combined SVM classifier:
                                      Extracellular  0.121
                                      PlasmaMembrane 0.021
                                      Cytoplasmic    0.914
                                      Cytoskeletal   0.008
                                      ER             0.026
                                      Golgi          0.011
                                      Lysosomal              0.009
                                      Mitochondrial  3.072 *
                                      Chloroplast    0.329
                                      Peroxisomal    0.094
                                      Vacuole        0.019
                                      Nuclear        0.377


**********************************************************************
************
SeqID: li|LinJ_10_0050_mRNA|
        CELLO prediction:
                       (predictor     location       reliable-index)
                       Composition    Mitochondrial  0.337
                       Di-peptide     Nuclear        0.724
                       part-Comp.     Mitochondrial  0.576
                       chemo-typy     Nuclear        0.753
                       Neighboring    Mitochondrial  0.368

        Combined SVM classifier:
                                      Extracellular  0.137
                                      PlasmaMembrane 0.054
                                      Cytoplasmic    0.303
                                      Cytoskeletal   0.017
                                      ER             0.013
                                      Golgi          0.008
                                      Lysosomal              0.010
                                      Mitochondrial  1.701 *
                                      Chloroplast    0.429
                                      Peroxisomal    0.045
                                      Vacuole        0.016
                                      Nuclear        2.267 *


**********************************************************************
************
SeqID: li|LinJ_28_1050_mRNA|
        CELLO prediction:
                       (predictor     location       reliable-index)
                       Composition    Cytoplasmic    0.374
                       Di-peptide     Chloroplast    0.328
                       part-Comp.     Cytoplasmic    0.854
                       chemo-typy     Cytoplasmic    0.824
                       Neighboring    Cytoplasmic    0.468

        Combined SVM classifier:
                                      Extracellular  0.084
                                      PlasmaMembrane 0.025
                                      Cytoplasmic    2.736 *
                                      Cytoskeletal   0.013
                                      ER             0.013
                                      Golgi          0.008
```

Table S1. Subcellular localization of proteins retrieved for TriTypDB database showing >80% homology among *L. infantum, L. major* and *L. braziliensis*.

```
                                       Lysosomal               0.015
                                       Mitochondrial   0.746
                                       Chloroplast     0.682
                                       Peroxisomal     0.049
                                       Vacuole         0.022
                                       Nuclear         0.606


**********************************************************************
************
SeqID: li|LinJ_13_0330_mRNA|
        CELLO prediction:
                        (predictor     location        reliable-index)
                        Composition    Cytoplasmic     0.739
                        Di-peptide     Cytoplasmic     0.775
                        part-Comp.     Cytoplasmic     0.914
                        chemo-typy     Chloroplast     0.354
                        Neighboring    Cytoplasmic     0.367

        Combined SVM classifier:
                                       Extracellular   0.271
                                       PlasmaMembrane  0.107
                                       Cytoplasmic     3.114 *
                                       Cytoskeletal    0.008
                                       ER              0.066
                                       Golgi           0.024
                                       Lysosomal               0.046
                                       Mitochondrial   0.187
                                       Chloroplast     0.507
                                       Peroxisomal     0.137
                                       Vacuole         0.119
                                       Nuclear         0.415


**********************************************************************
************
SeqID: li|LinJ_35_0140_mRNA|
        CELLO prediction:
                        (predictor     location        reliable-index)
                        Composition    Mitochondrial   0.864
                        Di-peptide     Mitochondrial   0.919
                        part-Comp.     Mitochondrial   0.554
                        chemo-typy     Mitochondrial   0.865
                        Neighboring    Mitochondrial   0.468

        Combined SVM classifier:
                                       Extracellular   0.199
                                       PlasmaMembrane  0.490
                                       Cytoplasmic     0.185
                                       Cytoskeletal    0.022
                                       ER              0.016
                                       Golgi           0.008
                                       Lysosomal               0.038
                                       Mitochondrial   3.669 *
                                       Chloroplast     0.059
                                       Peroxisomal     0.062
                                       Vacuole         0.009
                                       Nuclear         0.242
```

Table S1. Subcellular localization of proteins retrieved for TriTypDB database showing >80% homology among *L. infantum, L. major* and *L. braziliensis*.

```
**********************************************************************
************
SeqID: li|LinJ_31_2350_mRNA|
       CELLO prediction:
                      (predictor      location        reliable-index)
                      Composition     Cytoplasmic     0.409
                      Di-peptide      Mitochondrial   0.287
                      part-Comp.      Chloroplast     0.310
                      chemo-typy      Extracellular   0.404
                      Neighboring     Chloroplast     0.324

       Combined SVM classifier:
                                      Extracellular   1.064 *
                                      PlasmaMembrane  0.539
                                      Cytoplasmic     0.924
                                      Cytoskeletal    0.018
                                      ER              0.124
                                      Golgi           0.050
                                      Lysosomal               0.105
                                      Mitochondrial   0.768
                                      Chloroplast     1.076 *
                                      Peroxisomal     0.090
                                      Vacuole         0.070
                                      Nuclear         0.170


**********************************************************************
************
SeqID: li|LinJ_30_3240_mRNA|
       CELLO prediction:
                      (predictor      location        reliable-index)
                      Composition     Cytoplasmic     0.266
                      Di-peptide      Mitochondrial   0.459
                      part-Comp.      Nuclear         0.551
                      chemo-typy      Nuclear         0.467
                      Neighboring     Mitochondrial   0.374

       Combined SVM classifier:
                                      Extracellular   0.110
                                      PlasmaMembrane  0.055
                                      Cytoplasmic     0.846
                                      Cytoskeletal    0.022
                                      ER              0.017
                                      Golgi           0.013
                                      Lysosomal               0.015
                                      Mitochondrial   1.634 *
                                      Chloroplast     0.196
                                      Peroxisomal     0.048
                                      Vacuole         0.018
                                      Nuclear         2.026 *


**********************************************************************
************
SeqID: li|LinJ_13_0450_mRNA|
       CELLO prediction:
                      (predictor      location        reliable-index)
                      Composition     Mitochondrial   0.507
                      Di-peptide      Mitochondrial   0.796
                      part-Comp.      Mitochondrial   0.381
                      chemo-typy      Nuclear         0.549
```

Table S1. Subcellular localization of proteins retrieved for TriTypDB database showing >80% homology among *L. infantum, L. major* and *L. braziliensis*.

```
                       Neighboring     Mitochondrial   0.490

        Combined SVM classifier:
                                       Extracellular   0.068
                                       PlasmaMembrane  0.152
                                       Cytoplasmic     0.659
                                       Cytoskeletal    0.023
                                       ER              0.022
                                       Golgi           0.016
                                       Lysosomal               0.015
                                       Mitochondrial   2.409 *
                                       Chloroplast     0.357
                                       Peroxisomal     0.092
                                       Vacuole         0.018
                                       Nuclear         1.170


**********************************************************************
************
SeqID: li|LinJ_34_0460_mRNA|
        CELLO prediction:
                       (predictor      location        reliable-index)
                       Composition     Nuclear         0.588
                       Di-peptide      Mitochondrial   0.430
                       part-Comp.      Nuclear         0.738
                       chemo-typy      Nuclear         0.361
                       Neighboring     Nuclear         0.385

        Combined SVM classifier:
                                       Extracellular   0.318
                                       PlasmaMembrane  0.111
                                       Cytoplasmic     0.646
                                       Cytoskeletal    0.014
                                       ER              0.009
                                       Golgi           0.010
                                       Lysosomal               0.007
                                       Mitochondrial   1.129
                                       Chloroplast     0.223
                                       Peroxisomal     0.030
                                       Vacuole         0.008
                                       Nuclear         2.494 *


**********************************************************************
************
SeqID: li|LinJ_08_0830_mRNA|
        CELLO prediction:
                       (predictor      location        reliable-index)
                       Composition     Cytoplasmic     0.603
                       Di-peptide      Cytoplasmic     0.732
                       part-Comp.      Mitochondrial   0.438
                       chemo-typy      Cytoplasmic     0.596
                       Neighboring     Cytoplasmic     0.583

        Combined SVM classifier:
                                       Extracellular   0.006
                                       PlasmaMembrane  0.007
                                       Cytoplasmic     2.768 *
                                       Cytoskeletal    0.011
                                       ER              0.016
                                       Golgi           0.014
```

Table S1. Subcellular localization of proteins retrieved for TriTypDB database showing >80% homology among *L. infantum, L. major* and *L. braziliensis*.

```
                                       Lysosomal              0.002
                                       Mitochondrial  1.270
                                       Chloroplast    0.230
                                       Peroxisomal    0.213
                                       Vacuole        0.004
                                       Nuclear        0.458


**********************************************************************
************
SeqID: li|LinJ_28_2360_mRNA|
        CELLO prediction:
                        (predictor     location       reliable-index)
                        Composition    Extracellular  0.372
                        Di-peptide     Mitochondrial  0.747
                        part-Comp.     Nuclear        0.664
                        chemo-typy     Nuclear        0.441
                        Neighboring    Nuclear        0.506

        Combined SVM classifier:
                                       Extracellular  0.598
                                       PlasmaMembrane 0.083
                                       Cytoplasmic    0.465
                                       Cytoskeletal   0.020
                                       ER             0.011
                                       Golgi          0.009
                                       Lysosomal              0.010
                                       Mitochondrial  1.698 *
                                       Chloroplast    0.155
                                       Peroxisomal    0.033
                                       Vacuole        0.010
                                       Nuclear        1.909 *


**********************************************************************
************
SeqID: li|LinJ_31_1420_mRNA|
        CELLO prediction:
                        (predictor     location       reliable-index)
                        Composition    Extracellular  0.735
                        Di-peptide     Nuclear        0.358
                        part-Comp.     Extracellular  0.396
                        chemo-typy     Mitochondrial  0.412
                        Neighboring    Extracellular  0.788

        Combined SVM classifier:
                                       Extracellular  2.308 *
                                       PlasmaMembrane 0.266
                                       Cytoplasmic    0.440
                                       Cytoskeletal   0.017
                                       ER             0.026
                                       Golgi          0.021
                                       Lysosomal              0.040
                                       Mitochondrial  0.653
                                       Chloroplast    0.260
                                       Peroxisomal    0.028
                                       Vacuole        0.017
                                       Nuclear        0.923
```

Table S1. Subcellular localization of proteins retrieved for TriTypDB database showing >80% homology among *L. infantum, L. major* and *L. braziliensis*.

```
**********************************************************************
************
SeqID: li|LinJ_36_3020_mRNA|
        CELLO prediction:
                        (predictor      location        reliable-index)
                        Composition     Cytoplasmic     0.609
                        Di-peptide      Mitochondrial   0.453
                        part-Comp.      Cytoplasmic     0.512
                        chemo-typy      Nuclear         0.843
                        Neighboring     Mitochondrial   0.550

        Combined SVM classifier:
                                        Extracellular   0.102
                                        PlasmaMembrane  0.043
                                        Cytoplasmic     1.625 *
                                        Cytoskeletal    0.012
                                        ER              0.024
                                        Golgi           0.009
                                        Lysosomal               0.010
                                        Mitochondrial   1.484 *
                                        Chloroplast     0.098
                                        Peroxisomal     0.093
                                        Vacuole         0.014
                                        Nuclear         1.486 *


**********************************************************************
************
SeqID: li|LinJ_25_0740_mRNA|
        CELLO prediction:
                        (predictor      location        reliable-index)
                        Composition     Nuclear         0.737
                        Di-peptide      Cytoplasmic     0.459
                        part-Comp.      Cytoplasmic     0.481
                        chemo-typy      Cytoplasmic     0.527
                        Neighboring     Nuclear         0.937

        Combined SVM classifier:
                                        Extracellular   0.104
                                        PlasmaMembrane  0.027
                                        Cytoplasmic     1.681
                                        Cytoskeletal    0.012
                                        ER              0.038
                                        Golgi           0.176
                                        Lysosomal               0.017
                                        Mitochondrial   0.155
                                        Chloroplast     0.290
                                        Peroxisomal     0.046
                                        Vacuole         0.024
                                        Nuclear         2.431 *


**********************************************************************
************
SeqID: li|LinJ_26_2020_mRNA|
        CELLO prediction:
                        (predictor      location        reliable-index)
                        Composition     Cytoplasmic     0.340
                        Di-peptide      Nuclear         0.705
                        part-Comp.      Nuclear         0.602
                        chemo-typy      Nuclear         0.794
```

Table S1. Subcellular localization of proteins retrieved for TriTypDB database showing >80% homology among *L. infantum, L. major* and *L. braziliensis*.

```
                       Neighboring    Nuclear        0.405

        Combined SVM classifier:
                                      Extracellular  0.154
                                      PlasmaMembrane 0.067
                                      Cytoplasmic    0.813
                                      Cytoskeletal   0.014
                                      ER             0.017
                                      Golgi          0.010
                                      Lysosomal              0.012
                                      Mitochondrial  0.966
                                      Chloroplast    0.137
                                      Peroxisomal    0.029
                                      Vacuole        0.013
                                      Nuclear        2.768 *


*********************************************************************
************
SeqID: li|LinJ_35_4570_mRNA|
        CELLO prediction:
                       (predictor     location       reliable-index)
                       Composition    Extracellular  0.511
                       Di-peptide     Extracellular  0.419
                       part-Comp.     Extracellular  0.335
                       chemo-typy     Mitochondrial  0.476
                       Neighboring    Extracellular  0.739

        Combined SVM classifier:
                                      Extracellular  2.028 *
                                      PlasmaMembrane 0.101
                                      Cytoplasmic    0.719
                                      Cytoskeletal   0.014
                                      ER             0.021
                                      Golgi          0.014
                                      Lysosomal              0.022
                                      Mitochondrial  0.974
                                      Chloroplast    0.262
                                      Peroxisomal    0.025
                                      Vacuole        0.009
                                      Nuclear        0.810


*********************************************************************
************
SeqID: li|LinJ_36_3940_mRNA|
        CELLO prediction:
                       (predictor     location       reliable-index)
                       Composition    Extracellular  0.507
                       Di-peptide     Nuclear        0.683
                       part-Comp.     Nuclear        0.422
                       chemo-typy     Nuclear        0.773
                       Neighboring    Nuclear        0.556

        Combined SVM classifier:
                                      Extracellular  1.256
                                      PlasmaMembrane 0.042
                                      Cytoplasmic    0.460
                                      Cytoskeletal   0.011
                                      ER             0.018
                                      Golgi          0.008
```

Table S1. Subcellular localization of proteins retrieved for TriTypDB database showing >80% homology among *L. infantum, L. major* and *L. braziliensis*.

```
                                      Lysosomal               0.030
                                      Mitochondrial   0.335
                                      Chloroplast     0.169
                                      Peroxisomal     0.025
                                      Vacuole         0.016
                                      Nuclear         2.631 *


*********************************************************************
************
SeqID: li|LinJ_25_1350_mRNA|
       CELLO prediction:
                       (predictor     location        reliable-index)
                       Composition    Nuclear         0.441
                       Di-peptide     Nuclear         0.408
                       part-Comp.     Nuclear         0.566
                       chemo-typy     Nuclear         0.362
                       Neighboring    Nuclear         0.693

       Combined SVM classifier:
                                      Extracellular   0.649
                                      PlasmaMembrane  0.083
                                      Cytoplasmic     0.577
                                      Cytoskeletal    0.019
                                      ER              0.021
                                      Golgi           0.018
                                      Lysosomal               0.024
                                      Mitochondrial   0.909
                                      Chloroplast     0.201
                                      Peroxisomal     0.020
                                      Vacuole         0.010
                                      Nuclear         2.470 *


*********************************************************************
************
SeqID: li|LinJ_28_0870_mRNA|
       CELLO prediction:
                       (predictor     location        reliable-index)
                       Composition    Extracellular   0.516
                       Di-peptide     Nuclear         0.556
                       part-Comp.     Extracellular   0.351
                       chemo-typy     Extracellular   0.465
                       Neighboring    Extracellular   0.525

       Combined SVM classifier:
                                      Extracellular   1.986 *
                                      PlasmaMembrane  0.186
                                      Cytoplasmic     0.758
                                      Cytoskeletal    0.015
                                      ER              0.036
                                      Golgi           0.038
                                      Lysosomal               0.058
                                      Mitochondrial   0.263
                                      Chloroplast     0.317
                                      Peroxisomal     0.041
                                      Vacuole         0.043
                                      Nuclear         1.260
```

Table S1. Subcellular localization of proteins retrieved for TriTypDB database showing >80% homology among *L. infantum, L. major* and *L. braziliensis*.

```
**********************************************************************
************
SeqID: li|LinJ_30_3270_mRNA|
       CELLO prediction:
                       (predictor      location        reliable-index)
                       Composition     Mitochondrial   0.509
                       Di-peptide      Mitochondrial   0.739
                       part-Comp.      PlasmaMembrane  0.547
                       chemo-typy      Mitochondrial   0.721
                       Neighboring     Extracellular   0.329

       Combined SVM classifier:
                                       Extracellular   0.458
                                       PlasmaMembrane  0.769
                                       Cytoplasmic     0.603
                                       Cytoskeletal    0.016
                                       ER              0.010
                                       Golgi           0.010
                                       Lysosomal               0.037
                                       Mitochondrial   2.236 *
                                       Chloroplast     0.521
                                       Peroxisomal     0.077
                                       Vacuole         0.018
                                       Nuclear         0.220


**********************************************************************
************
SeqID: li|LinJ_04_0950_mRNA|
       CELLO prediction:
                       (predictor      location        reliable-index)
                       Composition     Nuclear         0.318
                       Di-peptide      Cytoplasmic     0.404
                       part-Comp.      Cytoplasmic     0.469
                       chemo-typy      Cytoplasmic     0.414
                       Neighboring     Mitochondrial   0.431

       Combined SVM classifier:
                                       Extracellular   0.045
                                       PlasmaMembrane  0.025
                                       Cytoplasmic     1.963 *
                                       Cytoskeletal    0.037
                                       ER              0.008
                                       Golgi           0.008
                                       Lysosomal               0.009
                                       Mitochondrial   1.718 *
                                       Chloroplast     0.126
                                       Peroxisomal     0.066
                                       Vacuole         0.008
                                       Nuclear         0.989


**********************************************************************
************
SeqID: li|LinJ_34_3470_mRNA|
       CELLO prediction:
                       (predictor      location        reliable-index)
                       Composition     Mitochondrial   0.899
                       Di-peptide      Mitochondrial   0.833
                       part-Comp.      Mitochondrial   0.759
                       chemo-typy      Mitochondrial   0.422
```

Table S1. Subcellular localization of proteins retrieved for TriTypDB database showing >80% homology among *L. infantum, L. major* and *L. braziliensis*.

```
                       Neighboring     Mitochondrial   0.893

        Combined SVM classifier:
                                       Extracellular   0.151
                                       PlasmaMembrane  0.015
                                       Cytoplasmic     0.509
                                       Cytoskeletal    0.007
                                       ER              0.016
                                       Golgi           0.004
                                       Lysosomal               0.110
                                       Mitochondrial   3.806 *
                                       Chloroplast     0.195
                                       Peroxisomal     0.078
                                       Vacuole         0.005
                                       Nuclear         0.104


**********************************************************************
************
SeqID: li|LinJ_01_0150_mRNA|
        CELLO prediction:
                       (predictor      location        reliable-index)
                       Composition     Mitochondrial   0.833
                       Di-peptide      Mitochondrial   0.710
                       part-Comp.      Mitochondrial   0.588
                       chemo-typy      Extracellular   0.300
                       Neighboring     Nuclear         0.655

        Combined SVM classifier:
                                       Extracellular   0.593
                                       PlasmaMembrane  0.102
                                       Cytoplasmic     0.235
                                       Cytoskeletal    0.045
                                       ER              0.015
                                       Golgi           0.023
                                       Lysosomal               0.022
                                       Mitochondrial   2.539 *
                                       Chloroplast     0.106
                                       Peroxisomal     0.055
                                       Vacuole         0.009
                                       Nuclear         1.256


**********************************************************************
************
SeqID: li|LinJ_07_0160_mRNA|
        CELLO prediction:
                       (predictor      location        reliable-index)
                       Composition     Extracellular   0.521
                       Di-peptide      Extracellular   0.512
                       part-Comp.      Extracellular   0.618
                       chemo-typy      Mitochondrial   0.429
                       Neighboring     Extracellular   0.395

        Combined SVM classifier:
                                       Extracellular   2.157 *
                                       PlasmaMembrane  0.083
                                       Cytoplasmic     0.275
                                       Cytoskeletal    0.010
                                       ER              0.059
                                       Golgi           0.007
```

Table S1. Subcellular localization of proteins retrieved for TriTypDB database showing >80% homology among *L. infantum, L. major* and *L. braziliensis*.

```
                                       Lysosomal              0.193
                                       Mitochondrial  1.607 *
                                       Chloroplast    0.054
                                       Peroxisomal    0.115
                                       Vacuole        0.018
                                       Nuclear        0.423


**********************************************************************
************
SeqID: li|LinJ_11_1200_mRNA|
       CELLO prediction:
                        (predictor     location       reliable-index)
                        Composition    Nuclear        0.853
                        Di-peptide     Extracellular  0.411
                        part-Comp.     Nuclear        0.564
                        chemo-typy     Cytoplasmic    0.711
                        Neighboring    Cytoplasmic    0.402

       Combined SVM classifier:
                                       Extracellular  0.631
                                       PlasmaMembrane 0.052
                                       Cytoplasmic    1.799 *
                                       Cytoskeletal   0.044
                                       ER             0.019
                                       Golgi          0.288
                                       Lysosomal              0.005
                                       Mitochondrial  0.068
                                       Chloroplast    0.138
                                       Peroxisomal    0.020
                                       Vacuole        0.005
                                       Nuclear        1.933 *


**********************************************************************
************
SeqID: li|LinJ_35_1670_mRNA|
       CELLO prediction:
                        (predictor     location       reliable-index)
                        Composition    Cytoplasmic    0.702
                        Di-peptide     Nuclear        0.801
                        part-Comp.     Mitochondrial  0.542
                        chemo-typy     Nuclear        0.481
                        Neighboring    Nuclear        0.641

       Combined SVM classifier:
                                       Extracellular  0.036
                                       PlasmaMembrane 0.023
                                       Cytoplasmic    1.045
                                       Cytoskeletal   0.013
                                       ER             0.009
                                       Golgi          0.007
                                       Lysosomal              0.005
                                       Mitochondrial  1.283
                                       Chloroplast    0.464
                                       Peroxisomal    0.022
                                       Vacuole        0.006
                                       Nuclear        2.087 *
```

Table S1. Subcellular localization of proteins retrieved for TriTypDB database showing >80% homology among *L. infantum, L. major* and *L. braziliensis.*

```
*********************************************************************
************
SeqID: li|LinJ_28_2040_mRNA|
       CELLO prediction:
                       (predictor      location        reliable-index)
                       Composition     Extracellular   0.277
                       Di-peptide      Nuclear         0.726
                       part-Comp.      Mitochondrial   0.428
                       chemo-typy      Cytoplasmic     0.331
                       Neighboring     Nuclear         0.408

       Combined SVM classifier:
                                       Extracellular   0.484
                                       PlasmaMembrane  0.061
                                       Cytoplasmic     0.996
                                       Cytoskeletal    0.044
                                       ER              0.015
                                       Golgi           0.015
                                       Lysosomal               0.011
                                       Mitochondrial   1.025
                                       Chloroplast     0.381
                                       Peroxisomal     0.051
                                       Vacuole         0.017
                                       Nuclear         1.901 *


*********************************************************************
************
SeqID: li|LinJ_25_2590_mRNA|
       CELLO prediction:
                       (predictor      location        reliable-index)
                       Composition     Chloroplast     0.473
                       Di-peptide      Cytoplasmic     0.414
                       part-Comp.      Cytoplasmic     0.694
                       chemo-typy      Chloroplast     0.621
                       Neighboring     Chloroplast     0.519

       Combined SVM classifier:
                                       Extracellular   0.301
                                       PlasmaMembrane  0.057
                                       Cytoplasmic     1.839 *
                                       Cytoskeletal    0.020
                                       ER              0.035
                                       Golgi           0.115
                                       Lysosomal               0.009
                                       Mitochondrial   0.356
                                       Chloroplast     1.841 *
                                       Peroxisomal     0.224
                                       Vacuole         0.037
                                       Nuclear         0.165


*********************************************************************
************
SeqID: li|LinJ_27_0620_mRNA|
       CELLO prediction:
                       (predictor      location        reliable-index)
                       Composition     PlasmaMembrane  0.390
                       Di-peptide      Extracellular   0.176
                       part-Comp.      Golgi           0.832
                       chemo-typy      Cytoplasmic     0.419
```

Table S1. Subcellular localization of proteins retrieved for TriTypDB database showing >80% homology among *L. infantum, L. major* and *L. braziliensis*.

```
                       Neighboring    Golgi          0.230

        Combined SVM classifier:
                                      Extracellular  0.410
                                      PlasmaMembrane 0.601
                                      Cytoplasmic    0.816
                                      Cytoskeletal   0.020
                                      ER             0.068
                                      Golgi          1.494 *
                                      Lysosomal              0.026
                                      Mitochondrial  0.404
                                      Chloroplast    0.362
                                      Peroxisomal    0.062
                                      Vacuole        0.060
                                      Nuclear        0.677


**********************************************************************
************
SeqID: li|LinJ_35_3990_mRNA|
        CELLO prediction:
                       (predictor     location       reliable-index)
                       Composition    Mitochondrial  0.712
                       Di-peptide     Cytoplasmic    0.436
                       part-Comp.     Mitochondrial  0.509
                       chemo-typy     Mitochondrial  0.351
                       Neighboring    Cytoplasmic    0.517

        Combined SVM classifier:
                                      Extracellular  0.047
                                      PlasmaMembrane 0.020
                                      Cytoplasmic    1.727 *
                                      Cytoskeletal   0.034
                                      ER             0.040
                                      Golgi          0.010
                                      Lysosomal              0.015
                                      Mitochondrial  2.160 *
                                      Chloroplast    0.063
                                      Peroxisomal    0.175
                                      Vacuole        0.010
                                      Nuclear        0.699


**********************************************************************
************
SeqID: li|LinJ_28_0210_mRNA|
        CELLO prediction:
                       (predictor     location       reliable-index)
                       Composition    Cytoplasmic    0.546
                       Di-peptide     Nuclear        0.905
                       part-Comp.     Nuclear        0.860
                       chemo-typy     Nuclear        0.823
                       Neighboring    Mitochondrial  0.455

        Combined SVM classifier:
                                      Extracellular  0.041
                                      PlasmaMembrane 0.031
                                      Cytoplasmic    0.682
                                      Cytoskeletal   0.014
                                      ER             0.010
                                      Golgi          0.002
```

Table S1. Subcellular localization of proteins retrieved for TriTypDB database showing >80% homology among *L. infantum, L. major* and *L. braziliensis*.

```
                                      Lysosomal               0.003
                                      Mitochondrial   0.763
                                      Chloroplast     0.041
                                      Peroxisomal     0.047
                                      Vacuole         0.003
                                      Nuclear         3.363 *


*********************************************************************
************
SeqID: li|LinJ_34_3720_mRNA|
       CELLO prediction:
                       (predictor     location        reliable-index)
                       Composition    Nuclear         0.653
                       Di-peptide     Mitochondrial   0.331
                       part-Comp.     Nuclear         0.343
                       chemo-typy     Nuclear         0.491
                       Neighboring    Nuclear         0.691

       Combined SVM classifier:
                                      Extracellular   0.652
                                      PlasmaMembrane  0.059
                                      Cytoplasmic     0.562
                                      Cytoskeletal    0.046
                                      ER              0.022
                                      Golgi           0.016
                                      Lysosomal               0.012
                                      Mitochondrial   0.878
                                      Chloroplast     0.222
                                      Peroxisomal     0.029
                                      Vacuole         0.020
                                      Nuclear         2.481 *


*********************************************************************
************
SeqID: li|LinJ_09_0030_mRNA|
       CELLO prediction:
                       (predictor     location        reliable-index)
                       Composition    Mitochondrial   0.532
                       Di-peptide     Mitochondrial   0.786
                       part-Comp.     Mitochondrial   0.517
                       chemo-typy     Nuclear         0.384
                       Neighboring    Chloroplast     0.318

       Combined SVM classifier:
                                      Extracellular   0.310
                                      PlasmaMembrane  0.376
                                      Cytoplasmic     0.414
                                      Cytoskeletal    0.017
                                      ER              0.056
                                      Golgi           0.011
                                      Lysosomal               0.049
                                      Mitochondrial   2.366 *
                                      Chloroplast     0.626
                                      Peroxisomal     0.097
                                      Vacuole         0.019
                                      Nuclear         0.660
```

Table S1. Subcellular localization of proteins retrieved for TriTypDB database showing >80% homology among *L. infantum, L. major* and *L. braziliensis*.

```
**********************************************************************
************
SeqID: li|LinJ_35_1540_mRNA|
        CELLO prediction:
                        (predictor      location        reliable-index)
                        Composition     Chloroplast     0.596
                        Di-peptide      Nuclear         0.613
                        part-Comp.      Mitochondrial   0.530
                        chemo-typy      Cytoplasmic     0.608
                        Neighboring     Cytoplasmic     0.358

        Combined SVM classifier:
                                        Extracellular   0.087
                                        PlasmaMembrane  0.099
                                        Cytoplasmic     1.308 *
                                        Cytoskeletal    0.013
                                        ER              0.027
                                        Golgi           0.015
                                        Lysosomal               0.011
                                        Mitochondrial   1.216 *
                                        Chloroplast     1.157 *
                                        Peroxisomal     0.180
                                        Vacuole         0.019
                                        Nuclear         0.867


**********************************************************************
************
SeqID: li|LinJ_08_1290_mRNA|
        CELLO prediction:
                        (predictor      location        reliable-index)
                        Composition     Cytoplasmic     0.501
                        Di-peptide      Cytoplasmic     0.827
                        part-Comp.      Cytoplasmic     0.833
                        chemo-typy      Cytoplasmic     0.673
                        Neighboring     Cytoplasmic     0.808

        Combined SVM classifier:
                                        Extracellular   0.113
                                        PlasmaMembrane  0.177
                                        Cytoplasmic     3.642 *
                                        Cytoskeletal    0.008
                                        ER              0.024
                                        Golgi           0.020
                                        Lysosomal               0.050
                                        Mitochondrial   0.074
                                        Chloroplast     0.108
                                        Peroxisomal     0.036
                                        Vacuole         0.043
                                        Nuclear         0.705


**********************************************************************
************
SeqID: li|LinJ_34_0900_mRNA|
        CELLO prediction:
                        (predictor      location        reliable-index)
                        Composition     PlasmaMembrane  0.943
                        Di-peptide      PlasmaMembrane  0.811
                        part-Comp.      PlasmaMembrane  0.390
                        chemo-typy      Extracellular   0.384
```

Table S1. Subcellular localization of proteins retrieved for TriTypDB database showing >80% homology among *L. infantum, L. major* and *L. braziliensis*.

```
                       Neighboring    Cytoplasmic     0.732

       Combined SVM classifier:
                                      Extracellular   0.435
                                      PlasmaMembrane  2.226  *
                                      Cytoplasmic     1.341
                                      Cytoskeletal    0.005
                                      ER              0.025
                                      Golgi           0.019
                                      Lysosomal               0.020
                                      Mitochondrial   0.125
                                      Chloroplast     0.213
                                      Peroxisomal     0.062
                                      Vacuole         0.021
                                      Nuclear         0.508


**********************************************************************
************
SeqID: li|LinJ_36_1690_mRNA|
       CELLO prediction:
                       (predictor     location        reliable-index)
                       Composition    Nuclear         0.538
                       Di-peptide     Nuclear         0.499
                       part-Comp.     Extracellular   0.781
                       chemo-typy     Nuclear         0.658
                       Neighboring    Extracellular   0.623

       Combined SVM classifier:
                                      Extracellular   2.009  *
                                      PlasmaMembrane  0.094
                                      Cytoplasmic     0.263
                                      Cytoskeletal    0.014
                                      ER              0.016
                                      Golgi           0.008
                                      Lysosomal               0.041
                                      Mitochondrial   0.245
                                      Chloroplast     0.076
                                      Peroxisomal     0.014
                                      Vacuole         0.012
                                      Nuclear         2.207  *


**********************************************************************
************
SeqID: li|LinJ_35_5420_mRNA|
       CELLO prediction:
                       (predictor     location        reliable-index)
                       Composition    Nuclear         0.509
                       Di-peptide     Nuclear         0.863
                       part-Comp.     Nuclear         0.637
                       chemo-typy     Nuclear         0.840
                       Neighboring    Nuclear         0.487

       Combined SVM classifier:
                                      Extracellular   0.440
                                      PlasmaMembrane  0.087
                                      Cytoplasmic     0.367
                                      Cytoskeletal    0.013
                                      ER              0.014
                                      Golgi           0.008
```

Table S1. Subcellular localization of proteins retrieved for TriTypDB database showing >80% homology among *L. infantum, L. major* and *L. braziliensis*.

```
                                       Lysosomal              0.011
                                       Mitochondrial  0.517
                                       Chloroplast    0.170
                                       Peroxisomal    0.024
                                       Vacuole        0.013
                                       Nuclear        3.336 *


**********************************************************************
************
SeqID: li|LinJ_35_3060_mRNA|
        CELLO prediction:
                        (predictor     location       reliable-index)
                        Composition    Cytoplasmic    0.596
                        Di-peptide     Cytoplasmic    0.630
                        part-Comp.     Cytoplasmic    0.715
                        chemo-typy     Mitochondrial  0.411
                        Neighboring    Cytoplasmic    0.538

        Combined SVM classifier:
                                       Extracellular  0.313
                                       PlasmaMembrane 0.043
                                       Cytoplasmic    2.681 *
                                       Cytoskeletal   0.011
                                       ER             0.197
                                       Golgi          0.086
                                       Lysosomal              0.020
                                       Mitochondrial  0.785
                                       Chloroplast    0.508
                                       Peroxisomal    0.034
                                       Vacuole        0.026
                                       Nuclear        0.296


**********************************************************************
************
SeqID: li|LinJ_25_1460_mRNA|
        CELLO prediction:
                        (predictor     location       reliable-index)
                        Composition    Cytoplasmic    0.551
                        Di-peptide     Cytoplasmic    0.644
                        part-Comp.     Cytoplasmic    0.353
                        chemo-typy     Nuclear        0.537
                        Neighboring    Cytoplasmic    0.803

        Combined SVM classifier:
                                       Extracellular  0.171
                                       PlasmaMembrane 0.214
                                       Cytoplasmic    2.506 *
                                       Cytoskeletal   0.019
                                       ER             0.056
                                       Golgi          0.023
                                       Lysosomal              0.034
                                       Mitochondrial  0.259
                                       Chloroplast    0.715
                                       Peroxisomal    0.115
                                       Vacuole        0.028
                                       Nuclear        0.860
```

Table S1. Subcellular localization of proteins retrieved for TriTypDB database showing >80% homology among *L. infantum, L. major* and *L. braziliensis.*

```
**********************************************************************
************
SeqID: li|LinJ_31_1930_mRNA|
       CELLO prediction:
                       (predictor      location        reliable-index)
                       Composition     Mitochondrial   0.496
                       Di-peptide      Nuclear         0.421
                       part-Comp.      Nuclear         0.591
                       chemo-typy      Nuclear         0.646
                       Neighboring     Extracellular   0.506

       Combined SVM classifier:
                                       Extracellular   0.609
                                       PlasmaMembrane  0.010
                                       Cytoplasmic     0.958
                                       Cytoskeletal    0.012
                                       ER              0.009
                                       Golgi           0.005
                                       Lysosomal               0.004
                                       Mitochondrial   0.936
                                       Chloroplast     0.152
                                       Peroxisomal     0.040
                                       Vacuole         0.004
                                       Nuclear         2.262 *


**********************************************************************
************
SeqID: li|LinJ_30_3710_mRNA|
       CELLO prediction:
                       (predictor      location        reliable-index)
                       Composition     Mitochondrial   0.400
                       Di-peptide      Mitochondrial   0.935
                       part-Comp.      Mitochondrial   0.814
                       chemo-typy      Nuclear         0.914
                       Neighboring     Mitochondrial   0.610

       Combined SVM classifier:
                                       Extracellular   0.067
                                       PlasmaMembrane  0.033
                                       Cytoplasmic     0.532
                                       Cytoskeletal    0.026
                                       ER              0.014
                                       Golgi           0.010
                                       Lysosomal               0.013
                                       Mitochondrial   2.825 *
                                       Chloroplast     0.077
                                       Peroxisomal     0.075
                                       Vacuole         0.015
                                       Nuclear         1.312


**********************************************************************
************
SeqID: li|LinJ_14_0530_mRNA|
       CELLO prediction:
                       (predictor      location        reliable-index)
                       Composition     Chloroplast     0.279
                       Di-peptide      Mitochondrial   0.384
                       part-Comp.      Cytoplasmic     0.414
                       chemo-typy      Nuclear         0.365
```

Table S1. Subcellular localization of proteins retrieved for TriTypDB database showing >80% homology among *L. infantum, L. major* and *L. braziliensis*.

```
                       Neighboring     Mitochondrial   0.390

        Combined SVM classifier:
                                       Extracellular   0.443
                                       PlasmaMembrane  0.047
                                       Cytoplasmic     1.151 *
                                       Cytoskeletal    0.037
                                       ER              0.036
                                       Golgi           0.029
                                       Lysosomal               0.013
                                       Mitochondrial   1.219 *
                                       Chloroplast     0.733
                                       Peroxisomal     0.058
                                       Vacuole         0.105
                                       Nuclear         1.128 *


**********************************************************************
************
SeqID: li|LinJ_24_0730_mRNA|
        CELLO prediction:
                       (predictor      location        reliable-index)
                       Composition     Nuclear         0.511
                       Di-peptide      Mitochondrial   0.379
                       part-Comp.      Nuclear         0.504
                       chemo-typy      Mitochondrial   0.463
                       Neighboring     Nuclear         0.385

        Combined SVM classifier:
                                       Extracellular   0.617
                                       PlasmaMembrane  0.123
                                       Cytoplasmic     0.785
                                       Cytoskeletal    0.041
                                       ER              0.027
                                       Golgi           0.020
                                       Lysosomal               0.027
                                       Mitochondrial   1.102 *
                                       Chloroplast     0.486
                                       Peroxisomal     0.071
                                       Vacuole         0.027
                                       Nuclear         1.675 *


**********************************************************************
************
SeqID: li|LinJ_20_1350_mRNA|
        CELLO prediction:
                       (predictor      location        reliable-index)
                       Composition     Cytoplasmic     0.518
                       Di-peptide      Cytoplasmic     0.291
                       part-Comp.      Cytoplasmic     0.550
                       chemo-typy      Nuclear         0.604
                       Neighboring     Cytoplasmic     0.602

        Combined SVM classifier:
                                       Extracellular   0.288
                                       PlasmaMembrane  0.045
                                       Cytoplasmic     2.054 *
                                       Cytoskeletal    0.017
                                       ER              0.081
                                       Golgi           0.034
```

Table S1. Subcellular localization of proteins retrieved for TriTypDB database showing >80% homology among *L. infantum, L. major* and *L. braziliensis*.

```
                                       Lysosomal              0.027
                                       Mitochondrial  0.273
                                       Chloroplast    0.525
                                       Peroxisomal    0.028
                                       Vacuole        0.038
                                       Nuclear        1.588 *


**********************************************************************
************
SeqID: li|LinJ_15_1530_mRNA|
        CELLO prediction:
                       (predictor     location       reliable-index)
                       Composition    Chloroplast    0.459
                       Di-peptide     Chloroplast    0.725
                       part-Comp.     Cytoplasmic    0.575
                       chemo-typy     Cytoplasmic    0.583
                       Neighboring    Nuclear        0.367

        Combined SVM classifier:
                                       Extracellular  0.493
                                       PlasmaMembrane 0.135
                                       Cytoplasmic    1.487 *
                                       Cytoskeletal   0.019
                                       ER             0.023
                                       Golgi          0.165
                                       Lysosomal              0.013
                                       Mitochondrial  0.346
                                       Chloroplast    1.564 *
                                       Peroxisomal    0.150
                                       Vacuole        0.013
                                       Nuclear        0.592


**********************************************************************
************
SeqID: li|LinJ_34_0980_mRNA|
        CELLO prediction:
                       (predictor     location       reliable-index)
                       Composition    Cytoplasmic    0.355
                       Di-peptide     Nuclear        0.312
                       part-Comp.     Mitochondrial  0.392
                       chemo-typy     Mitochondrial  0.520
                       Neighboring    Cytoplasmic    0.557

        Combined SVM classifier:
                                       Extracellular  0.349
                                       PlasmaMembrane 0.036
                                       Cytoplasmic    1.602 *
                                       Cytoskeletal   0.027
                                       ER             0.026
                                       Golgi          0.023
                                       Lysosomal              0.009
                                       Mitochondrial  1.633 *
                                       Chloroplast    0.236
                                       Peroxisomal    0.059
                                       Vacuole        0.023
                                       Nuclear        0.976
```

Table S1. Subcellular localization of proteins retrieved for TriTypDB database showing >80% homology among *L. infantum, L. major* and *L. braziliensis.*

```
**********************************************************************
************
SeqID: li|LinJ_31_2650_mRNA|
        CELLO prediction:
                        (predictor      location        reliable-index)
                        Composition     Extracellular   0.900
                        Di-peptide      Cytoplasmic     0.292
                        part-Comp.      Extracellular   0.780
                        chemo-typy      Cytoplasmic     0.421
                        Neighboring     Extracellular   0.520

        Combined SVM classifier:
                                        Extracellular   2.489 *
                                        PlasmaMembrane  0.037
                                        Cytoplasmic     0.928
                                        Cytoskeletal    0.012
                                        ER              0.017
                                        Golgi           0.012
                                        Lysosomal               0.035
                                        Mitochondrial   0.557
                                        Chloroplast     0.233
                                        Peroxisomal     0.028
                                        Vacuole         0.006
                                        Nuclear         0.646


**********************************************************************
************
SeqID: li|LinJ_09_1130_mRNA|
        CELLO prediction:
                        (predictor      location        reliable-index)
                        Composition     Cytoplasmic     0.691
                        Di-peptide      Mitochondrial   0.470
                        part-Comp.      Cytoplasmic     0.860
                        chemo-typy      Cytoplasmic     0.507
                        Neighboring     Nuclear         0.315

        Combined SVM classifier:
                                        Extracellular   0.078
                                        PlasmaMembrane  0.228
                                        Cytoplasmic     2.391 *
                                        Cytoskeletal    0.009
                                        ER              0.019
                                        Golgi           0.065
                                        Lysosomal               0.027
                                        Mitochondrial   0.835
                                        Chloroplast     0.471
                                        Peroxisomal     0.156
                                        Vacuole         0.016
                                        Nuclear         0.705


**********************************************************************
************
SeqID: li|LinJ_04_0930_mRNA|
        CELLO prediction:
                        (predictor      location        reliable-index)
                        Composition     Cytoplasmic     0.305
                        Di-peptide      Mitochondrial   0.445
                        part-Comp.      Nuclear         0.523
                        chemo-typy      Nuclear         0.558
```

Table S1. Subcellular localization of proteins retrieved for TriTypDB database showing >80% homology among *L. infantum, L. major* and *L. braziliensis*.

```
                       Neighboring    Nuclear        0.596

        Combined SVM classifier:
                                      Extracellular  0.189
                                      PlasmaMembrane 0.027
                                      Cytoplasmic    1.243
                                      Cytoskeletal   0.014
                                      ER             0.010
                                      Golgi          0.008
                                      Lysosomal             0.007
                                      Mitochondrial  1.033
                                      Chloroplast    0.388
                                      Peroxisomal    0.019
                                      Vacuole        0.006
                                      Nuclear        2.056 *


*********************************************************************
************
SeqID: li|LinJ_30_2760_mRNA|
        CELLO prediction:
                       (predictor     location       reliable-index)
                       Composition    Nuclear        0.362
                       Di-peptide     Mitochondrial  0.380
                       part-Comp.     Nuclear        0.434
                       chemo-typy     Mitochondrial  0.457
                       Neighboring    Nuclear        0.372

        Combined SVM classifier:
                                      Extracellular  0.482
                                      PlasmaMembrane 0.115
                                      Cytoplasmic    0.785
                                      Cytoskeletal   0.022
                                      ER             0.021
                                      Golgi          0.012
                                      Lysosomal             0.012
                                      Mitochondrial  1.284 *
                                      Chloroplast    0.378
                                      Peroxisomal    0.028
                                      Vacuole        0.015
                                      Nuclear        1.845 *


*********************************************************************
************
SeqID: li|LinJ_03_0940_mRNA|
        CELLO prediction:
                       (predictor     location       reliable-index)
                       Composition    Mitochondrial  0.357
                       Di-peptide     Mitochondrial  0.351
                       part-Comp.     Extracellular  0.291
                       chemo-typy     Chloroplast    0.347
                       Neighboring    Extracellular  0.888

        Combined SVM classifier:
                                      Extracellular  1.628 *
                                      PlasmaMembrane 0.401
                                      Cytoplasmic    0.518
                                      Cytoskeletal   0.018
                                      ER             0.031
                                      Golgi          0.032
```

Table S1. Subcellular localization of proteins retrieved for TriTypDB database showing >80% homology among *L. infantum, L. major* and *L. braziliensis*.

```
                                       Lysosomal              0.094
                                       Mitochondrial  1.155 *
                                       Chloroplast    0.666
                                       Peroxisomal    0.072
                                       Vacuole        0.015
                                       Nuclear        0.370


**********************************************************************
************
SeqID: li|LinJ_05_0070_mRNA|
        CELLO prediction:
                        (predictor      location       reliable-index)
                        Composition     PlasmaMembrane 0.401
                        Di-peptide      Chloroplast    0.341
                        part-Comp.      Cytoplasmic    0.431
                        chemo-typy      Mitochondrial  0.370
                        Neighboring     Nuclear        0.510

        Combined SVM classifier:
                                        Extracellular  0.280
                                        PlasmaMembrane 0.559
                                        Cytoplasmic    1.395 *
                                        Cytoskeletal   0.015
                                        ER             0.028
                                        Golgi          0.042
                                        Lysosomal              0.038
                                        Mitochondrial  0.929
                                        Chloroplast    0.632
                                        Peroxisomal    0.047
                                        Vacuole        0.017
                                        Nuclear        1.018 *


**********************************************************************
************
SeqID: li|LinJ_36_3530_mRNA|
        CELLO prediction:
                        (predictor      location       reliable-index)
                        Composition     Mitochondrial  0.741
                        Di-peptide      Mitochondrial  0.450
                        part-Comp.      Mitochondrial  0.446
                        chemo-typy      Mitochondrial  0.645
                        Neighboring     Mitochondrial  0.458

        Combined SVM classifier:
                                        Extracellular  0.090
                                        PlasmaMembrane 0.015
                                        Cytoplasmic    0.439
                                        Cytoskeletal   0.040
                                        ER             0.008
                                        Golgi          0.022
                                        Lysosomal              0.006
                                        Mitochondrial  2.740 *
                                        Chloroplast    0.295
                                        Peroxisomal    0.030
                                        Vacuole        0.007
                                        Nuclear        1.307
```

Table S1. Subcellular localization of proteins retrieved for TriTypDB database showing >80% homology among *L. infantum, L. major* and *L. braziliensis*.

```
**********************************************************************
************
SeqID: li|LinJ_27_1140_mRNA|
       CELLO prediction:
                      (predictor      location        reliable-index)
                      Composition     Cytoplasmic     0.460
                      Di-peptide      Cytoplasmic     0.695
                      part-Comp.      Chloroplast     0.235
                      chemo-typy      Extracellular   0.314
                      Neighboring     Cytoplasmic     0.532

       Combined SVM classifier:
                                      Extracellular   0.956
                                      PlasmaMembrane  0.260
                                      Cytoplasmic     2.080 *
                                      Cytoskeletal    0.019
                                      ER              0.074
                                      Golgi           0.045
                                      Lysosomal               0.133
                                      Mitochondrial   0.221
                                      Chloroplast     0.558
                                      Peroxisomal     0.307
                                      Vacuole         0.076
                                      Nuclear         0.269


**********************************************************************
************
SeqID: li|LinJ_21_2240_mRNA|
       CELLO prediction:
                      (predictor      location        reliable-index)
                      Composition     Cytoplasmic     0.475
                      Di-peptide      Cytoplasmic     0.813
                      part-Comp.      Cytoplasmic     0.791
                      chemo-typy      Cytoplasmic     0.627
                      Neighboring     Cytoplasmic     0.785

       Combined SVM classifier:
                                      Extracellular   0.126
                                      PlasmaMembrane  0.242
                                      Cytoplasmic     3.491 *
                                      Cytoskeletal    0.009
                                      ER              0.028
                                      Golgi           0.021
                                      Lysosomal               0.054
                                      Mitochondrial   0.082
                                      Chloroplast     0.112
                                      Peroxisomal     0.041
                                      Vacuole         0.054
                                      Nuclear         0.739


**********************************************************************
************
SeqID: li|LinJ_30_2860_mRNA|
       CELLO prediction:
                      (predictor      location        reliable-index)
                      Composition     PlasmaMembrane  0.444
                      Di-peptide      Extracellular   0.407
                      part-Comp.      Extracellular   0.471
                      chemo-typy      PlasmaMembrane  0.420
```

Table S1. Subcellular localization of proteins retrieved for TriTypDB database showing >80% homology among *L. infantum, L. major* and *L. braziliensis*.

```
                       Neighboring     PlasmaMembrane 0.367

        Combined SVM classifier:
                                       Extracellular  1.466 *
                                       PlasmaMembrane 1.621 *
                                       Cytoplasmic    0.098
                                       Cytoskeletal   0.013
                                       ER             0.036
                                       Golgi          0.020
                                       Lysosomal             0.068
                                       Mitochondrial  0.567
                                       Chloroplast    0.667
                                       Peroxisomal    0.042
                                       Vacuole        0.017
                                       Nuclear        0.384


**********************************************************************
************
SeqID: li|LinJ_11_0970_mRNA|
        CELLO prediction:
                       (predictor      location       reliable-index)
                       Composition     Chloroplast    0.446
                       Di-peptide      Nuclear        0.397
                       part-Comp.      Mitochondrial  0.696
                       chemo-typy      Mitochondrial  0.452
                       Neighboring     Chloroplast    0.284

        Combined SVM classifier:
                                       Extracellular  0.131
                                       PlasmaMembrane 0.022
                                       Cytoplasmic    0.852
                                       Cytoskeletal   0.025
                                       ER             0.035
                                       Golgi          0.022
                                       Lysosomal             0.019
                                       Mitochondrial  1.800 *
                                       Chloroplast    1.019
                                       Peroxisomal    0.055
                                       Vacuole        0.024
                                       Nuclear        0.996


**********************************************************************
************
SeqID: li|LinJ_32_0930_mRNA|
        CELLO prediction:
                       (predictor      location       reliable-index)
                       Composition     Mitochondrial  0.361
                       Di-peptide      Mitochondrial  0.412
                       part-Comp.      Nuclear        0.348
                       chemo-typy      Cytoplasmic    0.362
                       Neighboring     Nuclear        0.568

        Combined SVM classifier:
                                       Extracellular  0.258
                                       PlasmaMembrane 0.046
                                       Cytoplasmic    0.947
                                       Cytoskeletal   0.024
                                       ER             0.034
                                       Golgi          0.018
```

Table S1. Subcellular localization of proteins retrieved for TriTypDB database showing >80% homology among *L. infantum, L. major* and *L. braziliensis*.

```
                                       Lysosomal              0.027
                                       Mitochondrial  1.531 *
                                       Chloroplast    0.342
                                       Peroxisomal    0.163
                                       Vacuole        0.020
                                       Nuclear        1.591 *


**********************************************************************
************
SeqID: li|LinJ_34_2080_mRNA|
        CELLO prediction:
                        (predictor     location       reliable-index)
                        Composition    Nuclear        0.407
                        Di-peptide     Nuclear        0.441
                        part-Comp.     Extracellular  0.554
                        chemo-typy     Nuclear        0.463
                        Neighboring    Mitochondrial  0.333

        Combined SVM classifier:
                                       Extracellular  1.162 *
                                       PlasmaMembrane 0.042
                                       Cytoplasmic    0.405
                                       Cytoskeletal   0.012
                                       ER             0.016
                                       Golgi          0.007
                                       Lysosomal              0.011
                                       Mitochondrial  1.531 *
                                       Chloroplast    0.138
                                       Peroxisomal    0.036
                                       Vacuole        0.009
                                       Nuclear        1.630 *


**********************************************************************
************
SeqID: li|LinJ_32_2860_mRNA|
        CELLO prediction:
                        (predictor     location       reliable-index)
                        Composition    Extracellular  0.709
                        Di-peptide     Extracellular  0.390
                        part-Comp.     Extracellular  0.303
                        chemo-typy     Extracellular  0.483
                        Neighboring    Extracellular  0.323

        Combined SVM classifier:
                                       Extracellular  2.209 *
                                       PlasmaMembrane 0.410
                                       Cytoplasmic    0.770
                                       Cytoskeletal   0.015
                                       ER             0.040
                                       Golgi          0.140
                                       Lysosomal              0.051
                                       Mitochondrial  0.222
                                       Chloroplast    0.275
                                       Peroxisomal    0.106
                                       Vacuole        0.049
                                       Nuclear        0.713
```

Table S1. Subcellular localization of proteins retrieved for TriTypDB database showing >80% homology among *L. infantum, L. major* and *L. braziliensis*.

```
**********************************************************************
************
SeqID: li|LinJ_35_1750_mRNA|
       CELLO prediction:
                       (predictor      location        reliable-index)
                       Composition     PlasmaMembrane  0.968
                       Di-peptide      Mitochondrial   0.666
                       part-Comp.      PlasmaMembrane  0.651
                       chemo-typy      PlasmaMembrane  0.408
                       Neighboring     PlasmaMembrane  0.463

       Combined SVM classifier:
                                       Extracellular   0.138
                                       PlasmaMembrane  2.545 *
                                       Cytoplasmic     0.253
                                       Cytoskeletal    0.006
                                       ER              0.025
                                       Golgi           0.009
                                       Lysosomal               0.074
                                       Mitochondrial   1.279
                                       Chloroplast     0.545
                                       Peroxisomal     0.026
                                       Vacuole         0.028
                                       Nuclear         0.071


**********************************************************************
************
SeqID: li|LinJ_35_1380_mRNA|
       CELLO prediction:
                       (predictor      location        reliable-index)
                       Composition     Extracellular   0.923
                       Di-peptide      Nuclear         0.386
                       part-Comp.      Mitochondrial   0.445
                       chemo-typy      Nuclear         0.424
                       Neighboring     Extracellular   0.927

       Combined SVM classifier:
                                       Extracellular   2.273 *
                                       PlasmaMembrane  0.032
                                       Cytoplasmic     0.353
                                       Cytoskeletal    0.010
                                       ER              0.017
                                       Golgi           0.010
                                       Lysosomal               0.026
                                       Mitochondrial   1.094
                                       Chloroplast     0.096
                                       Peroxisomal     0.040
                                       Vacuole         0.015
                                       Nuclear         1.032


**********************************************************************
************
SeqID: li|LinJ_33_1430_mRNA|
       CELLO prediction:
                       (predictor      location        reliable-index)
                       Composition     Mitochondrial   0.820
                       Di-peptide      Mitochondrial   0.610
                       part-Comp.      Nuclear         0.323
                       chemo-typy      Nuclear         0.422
```

Table S1. Subcellular localization of proteins retrieved for TriTypDB database showing >80% homology among *L. infantum, L. major* and *L. braziliensis*.

```
                       Neighboring     Mitochondrial   0.496

        Combined SVM classifier:
                                       Extracellular   0.187
                                       PlasmaMembrane  0.218
                                       Cytoplasmic     0.917
                                       Cytoskeletal    0.013
                                       ER              0.031
                                       Golgi           0.031
                                       Lysosomal               0.027
                                       Mitochondrial   2.208 *
                                       Chloroplast     0.247
                                       Peroxisomal     0.188
                                       Vacuole         0.015
                                       Nuclear         0.919


*********************************************************************
************
SeqID: li|LinJ_28_1120_mRNA|
        CELLO prediction:
                       (predictor      location        reliable-index)
                       Composition     Mitochondrial   0.495
                       Di-peptide      Nuclear         0.604
                       part-Comp.      Mitochondrial   0.611
                       chemo-typy      Nuclear         0.361
                       Neighboring     Mitochondrial   0.521

        Combined SVM classifier:
                                       Extracellular   0.164
                                       PlasmaMembrane  0.049
                                       Cytoplasmic     0.684
                                       Cytoskeletal    0.013
                                       ER              0.014
                                       Golgi           0.014
                                       Lysosomal               0.009
                                       Mitochondrial   1.952 *
                                       Chloroplast     0.469
                                       Peroxisomal     0.069
                                       Vacuole         0.007
                                       Nuclear         1.556 *


*********************************************************************
************
SeqID: li|LinJ_11_0570_mRNA|
        CELLO prediction:
                       (predictor      location        reliable-index)
                       Composition     Extracellular   0.358
                       Di-peptide      Mitochondrial   0.687
                       part-Comp.      Mitochondrial   0.319
                       chemo-typy      Nuclear         0.643
                       Neighboring     Extracellular   0.639

        Combined SVM classifier:
                                       Extracellular   1.123 *
                                       PlasmaMembrane  0.647
                                       Cytoplasmic     0.276
                                       Cytoskeletal    0.014
                                       ER              0.144
                                       Golgi           0.012
```

Table S1. Subcellular localization of proteins retrieved for TriTypDB database showing >80% homology among *L. infantum, L. major* and *L. braziliensis*.

```
                                       Lysosomal              0.152
                                       Mitochondrial  1.476 *
                                       Chloroplast    0.291
                                       Peroxisomal    0.061
                                       Vacuole        0.036
                                       Nuclear        0.769


**********************************************************************
************
SeqID: li|LinJ_35_2230_mRNA|
        CELLO prediction:
                       (predictor     location       reliable-index)
                       Composition    Mitochondrial  0.363
                       Di-peptide     Chloroplast    0.368
                       part-Comp.     Mitochondrial  0.434
                       chemo-typy     Extracellular  0.535
                       Neighboring    Cytoplasmic    0.426

        Combined SVM classifier:
                                       Extracellular  0.649
                                       PlasmaMembrane 0.028
                                       Cytoplasmic    1.343 *
                                       Cytoskeletal   0.015
                                       ER             0.026
                                       Golgi          0.015
                                       Lysosomal              0.015
                                       Mitochondrial  1.329 *
                                       Chloroplast    0.961
                                       Peroxisomal    0.129
                                       Vacuole        0.019
                                       Nuclear        0.473


**********************************************************************
************
SeqID: li|LinJ_36_5660_mRNA|
        CELLO prediction:
                       (predictor     location       reliable-index)
                       Composition    Nuclear        0.476
                       Di-peptide     Cytoplasmic    0.749
                       part-Comp.     Cytoplasmic    0.335
                       chemo-typy     Cytoplasmic    0.505
                       Neighboring    Cytoplasmic    0.526

        Combined SVM classifier:
                                       Extracellular  0.141
                                       PlasmaMembrane 0.032
                                       Cytoplasmic    2.366 *
                                       Cytoskeletal   0.162
                                       ER             0.050
                                       Golgi          0.055
                                       Lysosomal              0.009
                                       Mitochondrial  0.360
                                       Chloroplast    0.279
                                       Peroxisomal    0.099
                                       Vacuole        0.066
                                       Nuclear        1.382
```

Table S1. Subcellular localization of proteins retrieved for TriTypDB database showing >80% homology among *L. infantum, L. major* and *L. braziliensis*.

```
**********************************************************************
************
SeqID: li|LinJ_27_2070_mRNA|
       CELLO prediction:
                       (predictor     location       reliable-index)
                       Composition    Extracellular  0.332
                       Di-peptide     Mitochondrial  0.566
                       part-Comp.     Nuclear        0.339
                       chemo-typy     Chloroplast    0.344
                       Neighboring    Extracellular  0.654

       Combined SVM classifier:
                                      Extracellular  1.235 *
                                      PlasmaMembrane 0.183
                                      Cytoplasmic    0.503
                                      Cytoskeletal   0.014
                                      ER             0.028
                                      Golgi          0.017
                                      Lysosomal              0.036
                                      Mitochondrial  1.422 *
                                      Chloroplast    0.687
                                      Peroxisomal    0.036
                                      Vacuole        0.023
                                      Nuclear        0.816


**********************************************************************
************
SeqID: li|LinJ_31_0960_mRNA|
       CELLO prediction:
                       (predictor     location       reliable-index)
                       Composition    Mitochondrial  0.273
                       Di-peptide     Extracellular  0.268
                       part-Comp.     Chloroplast    0.522
                       chemo-typy     PlasmaMembrane 0.477
                       Neighboring    Extracellular  0.299

       Combined SVM classifier:
                                      Extracellular  1.358 *
                                      PlasmaMembrane 0.858
                                      Cytoplasmic    0.612
                                      Cytoskeletal   0.015
                                      ER             0.067
                                      Golgi          0.022
                                      Lysosomal              0.022
                                      Mitochondrial  0.579
                                      Chloroplast    1.110 *
                                      Peroxisomal    0.049
                                      Vacuole        0.032
                                      Nuclear        0.276


**********************************************************************
************
SeqID: li|LinJ_32_3230_mRNA|
       CELLO prediction:
                       (predictor     location       reliable-index)
                       Composition    PlasmaMembrane 0.486
                       Di-peptide     Cytoplasmic    0.552
                       part-Comp.     Cytoplasmic    0.720
                       chemo-typy     Cytoplasmic    0.858
```

Table S1. Subcellular localization of proteins retrieved for TriTypDB database showing >80% homology among *L. infantum, L. major* and *L. braziliensis*.

```
                       Neighboring     Cytoplasmic     0.460

        Combined SVM classifier:
                                       Extracellular   0.581
                                       PlasmaMembrane  0.691
                                       Cytoplasmic     2.807 *
                                       Cytoskeletal    0.007
                                       ER              0.022
                                       Golgi           0.117
                                       Lysosomal               0.040
                                       Mitochondrial   0.077
                                       Chloroplast     0.115
                                       Peroxisomal     0.078
                                       Vacuole         0.015
                                       Nuclear         0.448


**********************************************************************
************
SeqID: li|LinJ_24_2220_mRNA|
        CELLO prediction:
                       (predictor      location        reliable-index)
                       Composition     Extracellular   0.301
                       Di-peptide      Extracellular   0.834
                       part-Comp.      Cytoplasmic     0.449
                       chemo-typy      Cytoplasmic     0.401
                       Neighboring     Cytoplasmic     0.321

        Combined SVM classifier:
                                       Extracellular   1.330 *
                                       PlasmaMembrane  0.359
                                       Cytoplasmic     1.397 *
                                       Cytoskeletal    0.027
                                       ER              0.044
                                       Golgi           0.055
                                       Lysosomal               0.093
                                       Mitochondrial   0.310
                                       Chloroplast     0.369
                                       Peroxisomal     0.092
                                       Vacuole         0.272
                                       Nuclear         0.651


**********************************************************************
************
SeqID: li|LinJ_16_0470_mRNA|
        CELLO prediction:
                       (predictor      location        reliable-index)
                       Composition     Cytoplasmic     0.725
                       Di-peptide      Mitochondrial   0.691
                       part-Comp.      Mitochondrial   0.501
                       chemo-typy      Nuclear         0.553
                       Neighboring     Mitochondrial   0.459

        Combined SVM classifier:
                                       Extracellular   0.026
                                       PlasmaMembrane  0.022
                                       Cytoplasmic     1.466
                                       Cytoskeletal    0.012
                                       ER              0.010
                                       Golgi           0.009
```

Table S1. Subcellular localization of proteins retrieved for TriTypDB database showing >80% homology among *L. infantum, L. major* and *L. braziliensis*.

```
                                      Lysosomal              0.008
                                      Mitochondrial   2.243 *
                                      Chloroplast     0.163
                                      Peroxisomal     0.108
                                      Vacuole         0.013
                                      Nuclear         0.919


*********************************************************************
************
SeqID: li|LinJ_36_0660_mRNA|
       CELLO prediction:
                       (predictor     location        reliable-index)
                       Composition    Cytoplasmic     0.692
                       Di-peptide     Nuclear         0.444
                       part-Comp.     Nuclear         0.347
                       chemo-typy     Extracellular   0.363
                       Neighboring    Nuclear         0.445

       Combined SVM classifier:
                                      Extracellular   0.612
                                      PlasmaMembrane  0.013
                                      Cytoplasmic     1.490 *
                                      Cytoskeletal    0.008
                                      ER              0.046
                                      Golgi           0.006
                                      Lysosomal              0.010
                                      Mitochondrial   0.748
                                      Chloroplast     0.202
                                      Peroxisomal     0.042
                                      Vacuole         0.037
                                      Nuclear         1.786 *


*********************************************************************
************
SeqID: li|LinJ_35_2960_mRNA|
       CELLO prediction:
                       (predictor     location        reliable-index)
                       Composition    Nuclear         0.811
                       Di-peptide     Cytoplasmic     0.376
                       part-Comp.     Mitochondrial   0.496
                       chemo-typy     Nuclear         0.584
                       Neighboring    Cytoplasmic     0.523

       Combined SVM classifier:
                                      Extracellular   0.164
                                      PlasmaMembrane  0.035
                                      Cytoplasmic     1.386 *
                                      Cytoskeletal    0.019
                                      ER              0.076
                                      Golgi           0.024
                                      Lysosomal              0.007
                                      Mitochondrial   0.935
                                      Chloroplast     0.266
                                      Peroxisomal     0.059
                                      Vacuole         0.018
                                      Nuclear         2.010 *
```

Table S1. Subcellular localization of proteins retrieved for TriTypDB database showing >80% homology among *L. infantum, L. major* and *L. braziliensis*.

```
*********************************************************************
************
SeqID: li|LinJ_34_3280_mRNA|
       CELLO prediction:
                       (predictor      location        reliable-index)
                       Composition     Cytoplasmic     0.691
                       Di-peptide      Cytoplasmic     0.783
                       part-Comp.      Cytoplasmic     0.854
                       chemo-typy      Cytoplasmic     0.704
                       Neighboring     Cytoplasmic     0.819

       Combined SVM classifier:
                                       Extracellular   0.013
                                       PlasmaMembrane  0.014
                                       Cytoplasmic     3.850 *
                                       Cytoskeletal    0.005
                                       ER              0.063
                                       Golgi           0.029
                                       Lysosomal               0.002
                                       Mitochondrial   0.536
                                       Chloroplast     0.357
                                       Peroxisomal     0.017
                                       Vacuole         0.005
                                       Nuclear         0.109


*********************************************************************
************
SeqID: li|LinJ_21_1310_mRNA|
       CELLO prediction:
                       (predictor      location        reliable-index)
                       Composition     Cytoplasmic     0.587
                       Di-peptide      Mitochondrial   0.769
                       part-Comp.      Mitochondrial   0.964
                       chemo-typy      Nuclear         0.571
                       Neighboring     Mitochondrial   0.855

       Combined SVM classifier:
                                       Extracellular   0.014
                                       PlasmaMembrane  0.011
                                       Cytoplasmic     0.934
                                       Cytoskeletal    0.009
                                       ER              0.005
                                       Golgi           0.006
                                       Lysosomal               0.004
                                       Mitochondrial   3.159 *
                                       Chloroplast     0.119
                                       Peroxisomal     0.052
                                       Vacuole         0.005
                                       Nuclear         0.682


*********************************************************************
************
SeqID: li|LinJ_19_1500_mRNA|
       CELLO prediction:
                       (predictor      location        reliable-index)
                       Composition     Nuclear         0.554
                       Di-peptide      Cytoplasmic     0.368
                       part-Comp.      Cytoplasmic     0.392
                       chemo-typy      Nuclear         0.264
```

Table S1. Subcellular localization of proteins retrieved for TriTypDB database showing >80% homology among *L. infantum, L. major* and *L. braziliensis*.

```
                       Neighboring     Extracellular  0.490

        Combined SVM classifier:
                                       Extracellular  0.886
                                       PlasmaMembrane 0.045
                                       Cytoplasmic    1.225 *
                                       Cytoskeletal   0.035
                                       ER             0.043
                                       Golgi          0.021
                                       Lysosomal              0.024
                                       Mitochondrial  0.804
                                       Chloroplast    0.271
                                       Peroxisomal    0.094
                                       Vacuole        0.021
                                       Nuclear        1.531 *


**********************************************************************
************
SeqID: li|LinJ_36_3430_mRNA|
        CELLO prediction:
                       (predictor      location       reliable-index)
                       Composition     Cytoplasmic    0.494
                       Di-peptide      Mitochondrial  0.569
                       part-Comp.      Mitochondrial  0.606
                       chemo-typy      Nuclear        0.811
                       Neighboring     Nuclear        0.413

        Combined SVM classifier:
                                       Extracellular  0.163
                                       PlasmaMembrane 0.065
                                       Cytoplasmic    1.107 *
                                       Cytoskeletal   0.010
                                       ER             0.034
                                       Golgi          0.011
                                       Lysosomal              0.016
                                       Mitochondrial  1.721 *
                                       Chloroplast    0.203
                                       Peroxisomal    0.034
                                       Vacuole        0.027
                                       Nuclear        1.610 *


**********************************************************************
************
SeqID: li|LinJ_32_3940_mRNA|
        CELLO prediction:
                       (predictor      location       reliable-index)
                       Composition     Mitochondrial  0.776
                       Di-peptide      Mitochondrial  0.508
                       part-Comp.      Nuclear        0.447
                       chemo-typy      Nuclear        0.396
                       Neighboring     Mitochondrial  0.408

        Combined SVM classifier:
                                       Extracellular  0.391
                                       PlasmaMembrane 0.044
                                       Cytoplasmic    0.702
                                       Cytoskeletal   0.015
                                       ER             0.020
                                       Golgi          0.016
```

Table S1. Subcellular localization of proteins retrieved for TriTypDB database showing >80% homology among *L. infantum, L. major* and *L. braziliensis*.

```
                                        Lysosomal              0.013
                                        Mitochondrial   2.144 *
                                        Chloroplast     0.192
                                        Peroxisomal     0.070
                                        Vacuole         0.006
                                        Nuclear         1.388


************************************************************************
************
SeqID: li|LinJ_27_0590_mRNA|
        CELLO prediction:
                        (predictor      location        reliable-index)
                        Composition     PlasmaMembrane  0.739
                        Di-peptide      Mitochondrial   0.622
                        part-Comp.      Mitochondrial   0.657
                        chemo-typy      Mitochondrial   0.590
                        Neighboring     Mitochondrial   0.352

        Combined SVM classifier:
                                        Extracellular   0.288
                                        PlasmaMembrane  1.380
                                        Cytoplasmic     0.192
                                        Cytoskeletal    0.008
                                        ER              0.013
                                        Golgi           0.007
                                        Lysosomal              0.121
                                        Mitochondrial   2.334 *
                                        Chloroplast     0.254
                                        Peroxisomal     0.160
                                        Vacuole         0.014
                                        Nuclear         0.227


************************************************************************
************
SeqID: li|LinJ_36_6950_mRNA|
        CELLO prediction:
                        (predictor      location        reliable-index)
                        Composition     Nuclear         0.516
                        Di-peptide      Extracellular   0.765
                        part-Comp.      Cytoplasmic     0.542
                        chemo-typy      Cytoplasmic     0.941
                        Neighboring     Cytoplasmic     0.717

        Combined SVM classifier:
                                        Extracellular   0.835
                                        PlasmaMembrane  0.347
                                        Cytoplasmic     2.606 *
                                        Cytoskeletal    0.029
                                        ER              0.007
                                        Golgi           0.048
                                        Lysosomal              0.007
                                        Mitochondrial   0.118
                                        Chloroplast     0.204
                                        Peroxisomal     0.037
                                        Vacuole         0.009
                                        Nuclear         0.754
```

Table S1. Subcellular localization of proteins retrieved for TriTypDB database showing >80% homology among *L. infantum, L. major* and *L. braziliensis*.

```
**********************************************************************
************
SeqID: li|LinJ_25_0370_mRNA|
       CELLO prediction:
                       (predictor      location        reliable-index)
                       Composition     Extracellular   0.309
                       Di-peptide      Mitochondrial   0.832
                       part-Comp.      Nuclear         0.340
                       chemo-typy      Nuclear         0.467
                       Neighboring     Mitochondrial   0.576

       Combined SVM classifier:
                                       Extracellular   0.395
                                       PlasmaMembrane  0.032
                                       Cytoplasmic     0.575
                                       Cytoskeletal    0.022
                                       ER              0.020
                                       Golgi           0.010
                                       Lysosomal               0.018
                                       Mitochondrial   1.874 *
                                       Chloroplast     0.478
                                       Peroxisomal     0.053
                                       Vacuole         0.024
                                       Nuclear         1.499 *


**********************************************************************
************
SeqID: li|LinJ_32_0440_mRNA|
       CELLO prediction:
                       (predictor      location        reliable-index)
                       Composition     Mitochondrial   0.382
                       Di-peptide      Nuclear         0.760
                       part-Comp.      Nuclear         0.558
                       chemo-typy      Mitochondrial   0.590
                       Neighboring     Mitochondrial   0.689

       Combined SVM classifier:
                                       Extracellular   0.044
                                       PlasmaMembrane  0.021
                                       Cytoplasmic     0.602
                                       Cytoskeletal    0.036
                                       ER              0.005
                                       Golgi           0.005
                                       Lysosomal               0.008
                                       Mitochondrial   2.178 *
                                       Chloroplast     0.087
                                       Peroxisomal     0.027
                                       Vacuole         0.006
                                       Nuclear         1.979 *


**********************************************************************
************
SeqID: li|LinJ_22_1260_mRNA|
       CELLO prediction:
                       (predictor      location        reliable-index)
                       Composition     Cytoplasmic     0.299
                       Di-peptide      Cytoplasmic     0.671
                       part-Comp.      Cytoplasmic     0.680
                       chemo-typy      Cytoplasmic     0.761
```

Table S1. Subcellular localization of proteins retrieved for TriTypDB database showing >80% homology among *L. infantum, L. major* and *L. braziliensis*.

```
                       Neighboring     Cytoplasmic     0.791

        Combined SVM classifier:
                                       Extracellular   0.319
                                       PlasmaMembrane  0.056
                                       Cytoplasmic     3.203 *
                                       Cytoskeletal    0.019
                                       ER              0.231
                                       Golgi           0.043
                                       Lysosomal               0.007
                                       Mitochondrial   0.173
                                       Chloroplast     0.406
                                       Peroxisomal     0.036
                                       Vacuole         0.034
                                       Nuclear         0.474


**********************************************************************
************
SeqID: li|LinJ_13_1020_mRNA|
        CELLO prediction:
                       (predictor      location        reliable-index)
                       Composition     Extracellular   0.197
                       Di-peptide      Extracellular   0.774
                       part-Comp.      Extracellular   0.314
                       chemo-typy      Chloroplast     0.279
                       Neighboring     Extracellular   0.357

        Combined SVM classifier:
                                       Extracellular   1.828 *
                                       PlasmaMembrane  0.389
                                       Cytoplasmic     0.598
                                       Cytoskeletal    0.023
                                       ER              0.046
                                       Golgi           0.059
                                       Lysosomal               0.036
                                       Mitochondrial   0.587
                                       Chloroplast     0.705
                                       Peroxisomal     0.039
                                       Vacuole         0.036
                                       Nuclear         0.656


**********************************************************************
************
SeqID: li|LinJ_32_3260_mRNA|
        CELLO prediction:
                       (predictor      location        reliable-index)
                       Composition     PlasmaMembrane  0.397
                       Di-peptide      Extracellular   0.613
                       part-Comp.      PlasmaMembrane  0.743
                       chemo-typy      PlasmaMembrane  0.757
                       Neighboring     PlasmaMembrane  0.545

        Combined SVM classifier:
                                       Extracellular   1.125
                                       PlasmaMembrane  2.749 *
                                       Cytoplasmic     0.079
                                       Cytoskeletal    0.011
                                       ER              0.020
                                       Golgi           0.013
```

Table S1. Subcellular localization of proteins retrieved for TriTypDB database showing >80% homology among *L. infantum, L. major* and *L. braziliensis*.

```
                                       Lysosomal               0.027
                                       Mitochondrial   0.365
                                       Chloroplast     0.373
                                       Peroxisomal     0.049
                                       Vacuole         0.030
                                       Nuclear         0.160


**********************************************************************
************
SeqID: li|LinJ_26_1080_mRNA|
        CELLO prediction:
                        (predictor      location        reliable-index)
                        Composition     Mitochondrial   0.689
                        Di-peptide      Mitochondrial   0.763
                        part-Comp.      Mitochondrial   0.602
                        chemo-typy      Mitochondrial   0.493
                        Neighboring     Mitochondrial   0.466

        Combined SVM classifier:
                                       Extracellular   0.090
                                       PlasmaMembrane  0.442
                                       Cytoplasmic     0.307
                                       Cytoskeletal    0.008
                                       ER              0.033
                                       Golgi           0.009
                                       Lysosomal               0.100
                                       Mitochondrial   3.013 *
                                       Chloroplast     0.607
                                       Peroxisomal     0.121
                                       Vacuole         0.018
                                       Nuclear         0.251


**********************************************************************
************
SeqID: li|LinJ_36_3560_mRNA|
        CELLO prediction:
                        (predictor      location        reliable-index)
                        Composition     Nuclear         0.424
                        Di-peptide      Mitochondrial   0.563
                        part-Comp.      Nuclear         0.497
                        chemo-typy      Nuclear         0.505
                        Neighboring     Nuclear         0.503

        Combined SVM classifier:
                                       Extracellular   0.265
                                       PlasmaMembrane  0.110
                                       Cytoplasmic     0.392
                                       Cytoskeletal    0.019
                                       ER              0.018
                                       Golgi           0.009
                                       Lysosomal               0.012
                                       Mitochondrial   1.556 *
                                       Chloroplast     0.319
                                       Peroxisomal     0.032
                                       Vacuole         0.014
                                       Nuclear         2.252 *
```

Table S1. Subcellular localization of proteins retrieved for TriTypDB database showing >80% homology among *L. infantum, L. major* and *L. braziliensis*.

```
**********************************************************************
************
SeqID: li|LinJ_31_0070_mRNA|
        CELLO prediction:
                        (predictor      location        reliable-index)
                        Composition     Mitochondrial   0.799
                        Di-peptide      Mitochondrial   0.794
                        part-Comp.      Mitochondrial   0.333
                        chemo-typy      Mitochondrial   0.509
                        Neighboring     Mitochondrial   0.396

        Combined SVM classifier:
                                        Extracellular   0.121
                                        PlasmaMembrane  0.187
                                        Cytoplasmic     0.576
                                        Cytoskeletal    0.014
                                        ER              0.016
                                        Golgi           0.011
                                        Lysosomal               0.022
                                        Mitochondrial   2.831 *
                                        Chloroplast     0.504
                                        Peroxisomal     0.087
                                        Vacuole         0.020
                                        Nuclear         0.611


**********************************************************************
************
SeqID: li|LinJ_08_1280_mRNA|
        CELLO prediction:
                        (predictor      location        reliable-index)
                        Composition     Cytoplasmic     0.524
                        Di-peptide      Cytoplasmic     0.857
                        part-Comp.      Cytoplasmic     0.816
                        chemo-typy      Cytoplasmic     0.648
                        Neighboring     Cytoplasmic     0.820

        Combined SVM classifier:
                                        Extracellular   0.100
                                        PlasmaMembrane  0.191
                                        Cytoplasmic     3.664 *
                                        Cytoskeletal    0.008
                                        ER              0.028
                                        Golgi           0.018
                                        Lysosomal               0.048
                                        Mitochondrial   0.067
                                        Chloroplast     0.104
                                        Peroxisomal     0.027
                                        Vacuole         0.047
                                        Nuclear         0.698


**********************************************************************
************
SeqID: li|LinJ_33_3340_mRNA|
        CELLO prediction:
                        (predictor      location        reliable-index)
                        Composition     Mitochondrial   0.558
                        Di-peptide      Extracellular   0.617
                        part-Comp.      Nuclear         0.556
                        chemo-typy      Nuclear         0.406
```

Table S1. Subcellular localization of proteins retrieved for TriTypDB database showing >80% homology among *L. infantum, L. major* and *L. braziliensis*.

```
                       Neighboring    Nuclear        0.362

        Combined SVM classifier:
                                      Extracellular  0.852
                                      PlasmaMembrane 0.041
                                      Cytoplasmic    0.833
                                      Cytoskeletal   0.018
                                      ER             0.020
                                      Golgi          0.032
                                      Lysosomal              0.011
                                      Mitochondrial  1.389 *
                                      Chloroplast    0.158
                                      Peroxisomal    0.053
                                      Vacuole        0.009
                                      Nuclear        1.584 *


*********************************************************************
************
SeqID: li|LinJ_29_0910_mRNA|
        CELLO prediction:
                       (predictor     location       reliable-index)
                       Composition    Cytoplasmic    0.446
                       Di-peptide     Mitochondrial  0.505
                       part-Comp.     Nuclear        0.468
                       chemo-typy     Nuclear        0.865
                       Neighboring    Mitochondrial  0.674

        Combined SVM classifier:
                                      Extracellular  0.149
                                      PlasmaMembrane 0.063
                                      Cytoplasmic    0.740
                                      Cytoskeletal   0.021
                                      ER             0.019
                                      Golgi          0.009
                                      Lysosomal              0.011
                                      Mitochondrial  1.714 *
                                      Chloroplast    0.132
                                      Peroxisomal    0.128
                                      Vacuole        0.026
                                      Nuclear        1.987 *


*********************************************************************
************
SeqID: li|LinJ_07_0210_mRNA|
        CELLO prediction:
                       (predictor     location       reliable-index)
                       Composition    Mitochondrial  0.355
                       Di-peptide     Cytoplasmic    0.539
                       part-Comp.     Mitochondrial  0.752
                       chemo-typy     Nuclear        0.414
                       Neighboring    Extracellular  0.365

        Combined SVM classifier:
                                      Extracellular  0.511
                                      PlasmaMembrane 0.054
                                      Cytoplasmic    0.910
                                      Cytoskeletal   0.010
                                      ER             0.037
                                      Golgi          0.011
```

Table S1. Subcellular localization of proteins retrieved for TriTypDB database showing >80% homology among *L. infantum, L. major* and *L. braziliensis*.

```
                                       Lysosomal              0.617
                                       Mitochondrial  1.472 *
                                       Chloroplast    0.438
                                       Peroxisomal    0.179
                                       Vacuole        0.083
                                       Nuclear        0.678


**********************************************************************
************
SeqID: li|LinJ_36_3960_mRNA|
        CELLO prediction:
                        (predictor     location       reliable-index)
                        Composition    Chloroplast    0.338
                        Di-peptide     Nuclear        0.663
                        part-Comp.     Mitochondrial  0.387
                        chemo-typy     Nuclear        0.681
                        Neighboring    Nuclear        0.300

        Combined SVM classifier:
                                       Extracellular  0.231
                                       PlasmaMembrane 0.033
                                       Cytoplasmic    0.918
                                       Cytoskeletal   0.013
                                       ER             0.021
                                       Golgi          0.011
                                       Lysosomal              0.010
                                       Mitochondrial  0.935
                                       Chloroplast    0.555
                                       Peroxisomal    0.117
                                       Vacuole        0.023
                                       Nuclear        2.133 *


**********************************************************************
************
SeqID: li|LinJ_15_0090_mRNA|
        CELLO prediction:
                        (predictor     location       reliable-index)
                        Composition    Cytoplasmic    0.713
                        Di-peptide     Cytoplasmic    0.916
                        part-Comp.     Mitochondrial  0.585
                        chemo-typy     Mitochondrial  0.433
                        Neighboring    Cytoplasmic    0.618

        Combined SVM classifier:
                                       Extracellular  0.012
                                       PlasmaMembrane 0.011
                                       Cytoplasmic    2.930 *
                                       Cytoskeletal   0.013
                                       ER             0.028
                                       Golgi          0.018
                                       Lysosomal              0.001
                                       Mitochondrial  1.435
                                       Chloroplast    0.220
                                       Peroxisomal    0.021
                                       Vacuole        0.002
                                       Nuclear        0.308
```

Table S1. Subcellular localization of proteins retrieved for TriTypDB database showing >80% homology among *L. infantum, L. major* and *L. braziliensis*.

```
********************************************************************
************
SeqID: li|LinJ_16_1330_mRNA|
       CELLO prediction:
                       (predictor      location        reliable-index)
                       Composition     Mitochondrial   0.545
                       Di-peptide      Mitochondrial   0.626
                       part-Comp.      Cytoplasmic     0.408
                       chemo-typy      Extracellular   0.678
                       Neighboring     Cytoplasmic     0.361

       Combined SVM classifier:
                                       Extracellular   0.988
                                       PlasmaMembrane  0.255
                                       Cytoplasmic     1.092 *
                                       Cytoskeletal    0.026
                                       ER              0.043
                                       Golgi           0.023
                                       Lysosomal               0.045
                                       Mitochondrial   1.576 *
                                       Chloroplast     0.562
                                       Peroxisomal     0.063
                                       Vacuole         0.015
                                       Nuclear         0.313

********************************************************************
************
SeqID: li|LinJ_36_3550_mRNA|
       CELLO prediction:
                       (predictor      location        reliable-index)
                       Composition     Nuclear         0.417
                       Di-peptide      Mitochondrial   0.462
                       part-Comp.      Nuclear         0.467
                       chemo-typy      Nuclear         0.535
                       Neighboring     Nuclear         0.475

       Combined SVM classifier:
                                       Extracellular   0.295
                                       PlasmaMembrane  0.122
                                       Cytoplasmic     0.431
                                       Cytoskeletal    0.018
                                       ER              0.019
                                       Golgi           0.010
                                       Lysosomal               0.014
                                       Mitochondrial   1.393
                                       Chloroplast     0.331
                                       Peroxisomal     0.035
                                       Vacuole         0.015
                                       Nuclear         2.319 *

********************************************************************
************
SeqID: li|LinJ_28_0780_mRNA|
       CELLO prediction:
                       (predictor      location        reliable-index)
                       Composition     Mitochondrial   0.669
                       Di-peptide      Mitochondrial   0.708
                       part-Comp.      Mitochondrial   0.931
                       chemo-typy      Mitochondrial   0.723
```

Table S1. Subcellular localization of proteins retrieved for TriTypDB database showing >80% homology among *L. infantum, L. major* and *L. braziliensis*.

```
                    Neighboring    Mitochondrial  0.895

        Combined SVM classifier:
                                   Extracellular  0.120
                                   PlasmaMembrane 0.009
                                   Cytoplasmic    0.114
                                   Cytoskeletal   0.010
                                   ER             0.011
                                   Golgi          0.005
                                   Lysosomal              0.007
                                   Mitochondrial  3.926 *
                                   Chloroplast    0.127
                                   Peroxisomal    0.048
                                   Vacuole        0.004
                                   Nuclear        0.619


**********************************************************************
************
SeqID: li|LinJ_01_0270_mRNA|
        CELLO prediction:
                    (predictor     location       reliable-index)
                    Composition    Chloroplast    0.338
                    Di-peptide     Cytoplasmic    0.318
                    part-Comp.     Cytoplasmic    0.428
                    chemo-typy     Cytoplasmic    0.525
                    Neighboring    Extracellular  0.394

        Combined SVM classifier:
                                   Extracellular  0.625
                                   PlasmaMembrane 0.570
                                   Cytoplasmic    1.705 *
                                   Cytoskeletal   0.036
                                   ER             0.039
                                   Golgi          0.041
                                   Lysosomal              0.025
                                   Mitochondrial  0.479
                                   Chloroplast    0.795
                                   Peroxisomal    0.166
                                   Vacuole        0.018
                                   Nuclear        0.502


**********************************************************************
************
SeqID: li|LinJ_35_3490_mRNA|
        CELLO prediction:
                    (predictor     location       reliable-index)
                    Composition    Nuclear        0.326
                    Di-peptide     Cytoplasmic    0.541
                    part-Comp.     Nuclear        0.351
                    chemo-typy     Mitochondrial  0.332
                    Neighboring    Cytoplasmic    0.450

        Combined SVM classifier:
                                   Extracellular  0.508
                                   PlasmaMembrane 0.113
                                   Cytoplasmic    1.457 *
                                   Cytoskeletal   0.020
                                   ER             0.041
                                   Golgi          0.045
```

Table S1. Subcellular localization of proteins retrieved for TriTypDB database showing >80% homology among *L. infantum, L. major* and *L. braziliensis*.

```
                                      Lysosomal              0.045
                                      Mitochondrial  0.600
                                      Chloroplast    0.719
                                      Peroxisomal    0.076
                                      Vacuole        0.062
                                      Nuclear        1.313 *


**********************************************************************
************
SeqID: li|LinJ_20_0250_mRNA|
       CELLO prediction:
                       (predictor     location       reliable-index)
                       Composition    Nuclear        0.746
                       Di-peptide     Nuclear        0.590
                       part-Comp.     Mitochondrial  0.492
                       chemo-typy     Cytoplasmic    0.610
                       Neighboring    Extracellular  0.188

       Combined SVM classifier:
                                      Extracellular  0.283
                                      PlasmaMembrane 0.212
                                      Cytoplasmic    1.357 *
                                      Cytoskeletal   0.015
                                      ER             0.074
                                      Golgi          0.017
                                      Lysosomal              0.009
                                      Mitochondrial  0.631
                                      Chloroplast    0.445
                                      Peroxisomal    0.037
                                      Vacuole        0.029
                                      Nuclear        1.892 *


**********************************************************************
************
SeqID: li|LinJ_20_0340_mRNA|
       CELLO prediction:
                       (predictor     location       reliable-index)
                       Composition    Mitochondrial  0.712
                       Di-peptide     Extracellular  0.378
                       part-Comp.     Nuclear        0.465
                       chemo-typy     Mitochondrial  0.620
                       Neighboring    Mitochondrial  0.432

       Combined SVM classifier:
                                      Extracellular  0.837
                                      PlasmaMembrane 0.341
                                      Cytoplasmic    0.277
                                      Cytoskeletal   0.011
                                      ER             0.083
                                      Golgi          0.016
                                      Lysosomal              0.022
                                      Mitochondrial  2.428 *
                                      Chloroplast    0.097
                                      Peroxisomal    0.091
                                      Vacuole        0.013
                                      Nuclear        0.785
```

Table S1. Subcellular localization of proteins retrieved for TriTypDB database showing >80% homology among *L. infantum, L. major* and *L. braziliensis.*

```
**********************************************************************
************
SeqID: li|LinJ_24_0040_mRNA|
        CELLO prediction:
                        (predictor      location        reliable-index)
                        Composition     Mitochondrial   0.429
                        Di-peptide      Nuclear         0.736
                        part-Comp.      Nuclear         0.540
                        chemo-typy      Mitochondrial   0.646
                        Neighboring     Mitochondrial   0.705

        Combined SVM classifier:
                                        Extracellular   0.050
                                        PlasmaMembrane  0.020
                                        Cytoplasmic     0.539
                                        Cytoskeletal    0.034
                                        ER              0.005
                                        Golgi           0.006
                                        Lysosomal               0.008
                                        Mitochondrial   2.340 *
                                        Chloroplast     0.087
                                        Peroxisomal     0.030
                                        Vacuole         0.007
                                        Nuclear         1.875 *


**********************************************************************
************
SeqID: li|LinJ_03_0210_mRNA|
        CELLO prediction:
                        (predictor      location        reliable-index)
                        Composition     Mitochondrial   0.651
                        Di-peptide      Mitochondrial   0.618
                        part-Comp.      Mitochondrial   0.464
                        chemo-typy      Nuclear         0.346
                        Neighboring     Mitochondrial   0.697

        Combined SVM classifier:
                                        Extracellular   0.046
                                        PlasmaMembrane  0.277
                                        Cytoplasmic     0.528
                                        Cytoskeletal    0.031
                                        ER              0.012
                                        Golgi           0.010
                                        Lysosomal               0.008
                                        Mitochondrial   2.747 *
                                        Chloroplast     0.210
                                        Peroxisomal     0.155
                                        Vacuole         0.009
                                        Nuclear         0.967


**********************************************************************
************
SeqID: li|LinJ_14_1010_mRNA|
        CELLO prediction:
                        (predictor      location        reliable-index)
                        Composition     Mitochondrial   0.918
                        Di-peptide      Mitochondrial   0.306
                        part-Comp.      Mitochondrial   0.718
                        chemo-typy      Mitochondrial   0.673
```

Table S1. Subcellular localization of proteins retrieved for TriTypDB database showing >80% homology among *L. infantum, L. major* and *L. braziliensis*.

```
                        Neighboring     Mitochondrial   0.797

        Combined SVM classifier:
                                        Extracellular   0.067
                                        PlasmaMembrane  0.446
                                        Cytoplasmic     0.147
                                        Cytoskeletal    0.013
                                        ER              0.010
                                        Golgi           0.008
                                        Lysosomal               0.008
                                        Mitochondrial   3.413 *
                                        Chloroplast     0.450
                                        Peroxisomal     0.165
                                        Vacuole         0.025
                                        Nuclear         0.248


************************************************************************
************
SeqID: li|LinJ_35_0210_mRNA|
        CELLO prediction:
                        (predictor      location        reliable-index)
                        Composition     PlasmaMembrane  0.547
                        Di-peptide      PlasmaMembrane  0.424
                        part-Comp.      PlasmaMembrane  0.511
                        chemo-typy      Chloroplast     0.371
                        Neighboring     PlasmaMembrane  0.339

        Combined SVM classifier:
                                        Extracellular   1.099
                                        PlasmaMembrane  1.879 *
                                        Cytoplasmic     0.341
                                        Cytoskeletal    0.012
                                        ER              0.028
                                        Golgi           0.024
                                        Lysosomal               0.023
                                        Mitochondrial   0.421
                                        Chloroplast     0.440
                                        Peroxisomal     0.079
                                        Vacuole         0.013
                                        Nuclear         0.641


************************************************************************
************
SeqID: li|LinJ_36_1680_mRNA|
        CELLO prediction:
                        (predictor      location        reliable-index)
                        Composition     Nuclear         0.538
                        Di-peptide      Extracellular   0.515
                        part-Comp.      Extracellular   0.667
                        chemo-typy      Nuclear         0.658
                        Neighboring     Extracellular   0.563

        Combined SVM classifier:
                                        Extracellular   1.882 *
                                        PlasmaMembrane  0.100
                                        Cytoplasmic     0.263
                                        Cytoskeletal    0.014
                                        ER              0.016
                                        Golgi           0.008
```

Table S1. Subcellular localization of proteins retrieved for TriTypDB database showing >80% homology among *L. infantum, L. major* and *L. braziliensis*.

```
                                        Lysosomal               0.041
                                        Mitochondrial   0.254
                                        Chloroplast     0.076
                                        Peroxisomal     0.013
                                        Vacuole         0.012
                                        Nuclear         2.320 *


*********************************************************************
************
SeqID: li|LinJ_31_2890_mRNA|
        CELLO prediction:
                        (predictor      location        reliable-index)
                        Composition     Chloroplast     0.280
                        Di-peptide      Mitochondrial   0.777
                        part-Comp.      Chloroplast     0.544
                        chemo-typy      Cytoplasmic     0.452
                        Neighboring     Mitochondrial   0.417

        Combined SVM classifier:
                                        Extracellular   0.491
                                        PlasmaMembrane  0.173
                                        Cytoplasmic     0.889
                                        Cytoskeletal    0.017
                                        ER              0.143
                                        Golgi           0.027
                                        Lysosomal               0.072
                                        Mitochondrial   1.587 *
                                        Chloroplast     1.152 *
                                        Peroxisomal     0.115
                                        Vacuole         0.032
                                        Nuclear         0.302


*********************************************************************
************
SeqID: li|LinJ_04_1190_mRNA|
        CELLO prediction:
                        (predictor      location        reliable-index)
                        Composition     Nuclear         0.597
                        Di-peptide      Nuclear         0.645
                        part-Comp.      Nuclear         0.955
                        chemo-typy      Extracellular   0.805
                        Neighboring     Nuclear         0.521

        Combined SVM classifier:
                                        Extracellular   1.162
                                        PlasmaMembrane  0.131
                                        Cytoplasmic     0.492
                                        Cytoskeletal    0.013
                                        ER              0.039
                                        Golgi           0.014
                                        Lysosomal               0.018
                                        Mitochondrial   0.174
                                        Chloroplast     0.106
                                        Peroxisomal     0.045
                                        Vacuole         0.045
                                        Nuclear         2.761 *
```

Table S1. Subcellular localization of proteins retrieved for TriTypDB database showing >80% homology among *L. infantum, L. major* and *L. braziliensis.*

```
**********************************************************************
************
SeqID: li|LinJ_32_3370_mRNA|
        CELLO prediction:
                        (predictor      location        reliable-index)
                        Composition     Cytoplasmic     0.419
                        Di-peptide      Extracellular   0.228
                        part-Comp.      PlasmaMembrane  0.256
                        chemo-typy      Chloroplast     0.355
                        Neighboring     Extracellular   0.366

        Combined SVM classifier:
                                        Extracellular   1.033 *
                                        PlasmaMembrane  0.389
                                        Cytoplasmic     0.938
                                        Cytoskeletal    0.039
                                        ER              0.032
                                        Golgi           0.038
                                        Lysosomal               0.401
                                        Mitochondrial   0.649
                                        Chloroplast     0.827
                                        Peroxisomal     0.092
                                        Vacuole         0.050
                                        Nuclear         0.512


**********************************************************************
************
SeqID: li|LinJ_28_1620_mRNA|
        CELLO prediction:
                        (predictor      location        reliable-index)
                        Composition     PlasmaMembrane  0.822
                        Di-peptide      PlasmaMembrane  0.577
                        part-Comp.      PlasmaMembrane  0.833
                        chemo-typy      PlasmaMembrane  0.887
                        Neighboring     PlasmaMembrane  0.763

        Combined SVM classifier:
                                        Extracellular   0.291
                                        PlasmaMembrane  3.883 *
                                        Cytoplasmic     0.038
                                        Cytoskeletal    0.004
                                        ER              0.015
                                        Golgi           0.006
                                        Lysosomal               0.029
                                        Mitochondrial   0.426
                                        Chloroplast     0.198
                                        Peroxisomal     0.036
                                        Vacuole         0.005
                                        Nuclear         0.068


**********************************************************************
************
SeqID: li|LinJ_36_5970_mRNA|
        CELLO prediction:
                        (predictor      location        reliable-index)
                        Composition     Cytoplasmic     0.395
                        Di-peptide      Nuclear         0.707
                        part-Comp.      Nuclear         0.475
                        chemo-typy      Nuclear         0.907
```

Table S1. Subcellular localization of proteins retrieved for TriTypDB database showing >80% homology among *L. infantum, L. major* and *L. braziliensis*.

```
                       Neighboring    Nuclear        0.369

       Combined SVM classifier:
                                      Extracellular  0.253
                                      PlasmaMembrane 0.059
                                      Cytoplasmic    0.900
                                      Cytoskeletal   0.014
                                      ER             0.020
                                      Golgi          0.012
                                      Lysosomal              0.013
                                      Mitochondrial  0.883
                                      Chloroplast    0.215
                                      Peroxisomal    0.024
                                      Vacuole        0.015
                                      Nuclear        2.592 *


*********************************************************************
************
SeqID: li|LinJ_34_1880_mRNA|
       CELLO prediction:
                       (predictor     location       reliable-index)
                       Composition    Chloroplast    0.331
                       Di-peptide     Cytoplasmic    0.517
                       part-Comp.     Chloroplast    0.862
                       chemo-typy     Chloroplast    0.425
                       Neighboring    Cytoplasmic    0.334

       Combined SVM classifier:
                                      Extracellular  0.075
                                      PlasmaMembrane 0.311
                                      Cytoplasmic    1.158
                                      Cytoskeletal   0.015
                                      ER             0.020
                                      Golgi          0.021
                                      Lysosomal              0.006
                                      Mitochondrial  0.822
                                      Chloroplast    2.081 *
                                      Peroxisomal    0.018
                                      Vacuole        0.006
                                      Nuclear        0.467


*********************************************************************
************
SeqID: li|LinJ_36_4570_mRNA|
       CELLO prediction:
                       (predictor     location       reliable-index)
                       Composition    Mitochondrial  0.474
                       Di-peptide     Cytoplasmic    0.612
                       part-Comp.     Mitochondrial  0.652
                       chemo-typy     Cytoplasmic    0.795
                       Neighboring    Cytoplasmic    0.792

       Combined SVM classifier:
                                      Extracellular  0.009
                                      PlasmaMembrane 0.006
                                      Cytoplasmic    2.623 *
                                      Cytoskeletal   0.010
                                      ER             0.018
                                      Golgi          0.020
```

Table S1. Subcellular localization of proteins retrieved for TriTypDB database showing >80% homology among *L. infantum, L. major* and *L. braziliensis*.

```
                                       Lysosomal              0.003
                                       Mitochondrial  1.519
                                       Chloroplast    0.449
                                       Peroxisomal    0.029
                                       Vacuole        0.004
                                       Nuclear        0.310


**********************************************************************
************
SeqID: li|LinJ_32_0870_mRNA|
        CELLO prediction:
                        (predictor     location       reliable-index)
                        Composition    PlasmaMembrane 0.657
                        Di-peptide     Extracellular  0.277
                        part-Comp.     PlasmaMembrane 0.841
                        chemo-typy     PlasmaMembrane 0.289
                        Neighboring    PlasmaMembrane 0.519

        Combined SVM classifier:
                                       Extracellular  0.684
                                       PlasmaMembrane 2.486 *
                                       Cytoplasmic    0.149
                                       Cytoskeletal   0.012
                                       ER             0.023
                                       Golgi          0.128
                                       Lysosomal              0.042
                                       Mitochondrial  0.429
                                       Chloroplast    0.356
                                       Peroxisomal    0.052
                                       Vacuole        0.014
                                       Nuclear        0.624


**********************************************************************
************
SeqID: li|LinJ_30_1650_mRNA|
        CELLO prediction:
                        (predictor     location       reliable-index)
                        Composition    PlasmaMembrane 0.715
                        Di-peptide     Mitochondrial  0.404
                        part-Comp.     Extracellular  0.329
                        chemo-typy     Mitochondrial  0.285
                        Neighboring    PlasmaMembrane 0.526

        Combined SVM classifier:
                                       Extracellular  1.144
                                       PlasmaMembrane 1.952 *
                                       Cytoplasmic    0.182
                                       Cytoskeletal   0.016
                                       ER             0.026
                                       Golgi          0.013
                                       Lysosomal              0.074
                                       Mitochondrial  0.891
                                       Chloroplast    0.464
                                       Peroxisomal    0.051
                                       Vacuole        0.088
                                       Nuclear        0.099
```

Table S1. Subcellular localization of proteins retrieved for TriTypDB database showing >80% homology among *L. infantum, L. major* and *L. braziliensis*.

```
**********************************************************************
************
SeqID: li|LinJ_28_3220_mRNA|
       CELLO prediction:
                       (predictor      location        reliable-index)
                       Composition     Mitochondrial   0.591
                       Di-peptide      Mitochondrial   0.732
                       part-Comp.      Mitochondrial   0.744
                       chemo-typy      Mitochondrial   0.569
                       Neighboring     Mitochondrial   0.751

       Combined SVM classifier:
                                       Extracellular   0.172
                                       PlasmaMembrane  0.021
                                       Cytoplasmic     0.361
                                       Cytoskeletal    0.010
                                       ER              0.016
                                       Golgi           0.006
                                       Lysosomal               0.012
                                       Mitochondrial   3.386 *
                                       Chloroplast     0.206
                                       Peroxisomal     0.072
                                       Vacuole         0.016
                                       Nuclear         0.723


**********************************************************************
************
SeqID: li|LinJ_08_0340_mRNA|
       CELLO prediction:
                       (predictor      location        reliable-index)
                       Composition     Extracellular   0.722
                       Di-peptide      Mitochondrial   0.265
                       part-Comp.      Cytoplasmic     0.303
                       chemo-typy      Nuclear         0.419
                       Neighboring     Extracellular   0.366

       Combined SVM classifier:
                                       Extracellular   1.580 *
                                       PlasmaMembrane  0.045
                                       Cytoplasmic     0.924
                                       Cytoskeletal    0.017
                                       ER              0.029
                                       Golgi           0.028
                                       Lysosomal               0.020
                                       Mitochondrial   0.827
                                       Chloroplast     0.321
                                       Peroxisomal     0.033
                                       Vacuole         0.012
                                       Nuclear         1.163 *


**********************************************************************
************
SeqID: li|LinJ_36_6680_mRNA|
       CELLO prediction:
                       (predictor      location        reliable-index)
                       Composition     Cytoplasmic     0.537
                       Di-peptide      Cytoplasmic     0.441
                       part-Comp.      Mitochondrial   0.639
                       chemo-typy      Nuclear         0.952
```

Table S1. Subcellular localization of proteins retrieved for TriTypDB database showing >80% homology among *L. infantum, L. major* and *L. braziliensis*.

```
                        Neighboring     Cytoplasmic     0.491

        Combined SVM classifier:
                                        Extracellular   0.014
                                        PlasmaMembrane  0.010
                                        Cytoplasmic     1.508 *
                                        Cytoskeletal    0.024
                                        ER              0.010
                                        Golgi           0.007
                                        Lysosomal               0.004
                                        Mitochondrial   1.479 *
                                        Chloroplast     0.070
                                        Peroxisomal     0.035
                                        Vacuole         0.006
                                        Nuclear         1.831 *


************************************************************************
SeqID: li|LinJ_24_1240_mRNA|
        CELLO prediction:
                        (predictor      location        reliable-index)
                        Composition     Nuclear         0.314
                        Di-peptide      Cytoplasmic     0.375
                        part-Comp.      Nuclear         0.431
                        chemo-typy      Nuclear         0.426
                        Neighboring     Nuclear         0.606

        Combined SVM classifier:
                                        Extracellular   0.258
                                        PlasmaMembrane  0.051
                                        Cytoplasmic     1.353
                                        Cytoskeletal    0.039
                                        ER              0.032
                                        Golgi           0.013
                                        Lysosomal               0.013
                                        Mitochondrial   0.895
                                        Chloroplast     0.211
                                        Peroxisomal     0.061
                                        Vacuole         0.009
                                        Nuclear         2.064 *


************************************************************************
SeqID: li|LinJ_22_1300_mRNA|
        CELLO prediction:
                        (predictor      location        reliable-index)
                        Composition     Extracellular   0.496
                        Di-peptide      Cytoplasmic     0.730
                        part-Comp.      Extracellular   0.791
                        chemo-typy      Extracellular   0.835
                        Neighboring     Cytoplasmic     0.330

        Combined SVM classifier:
                                        Extracellular   2.281 *
                                        PlasmaMembrane  0.057
                                        Cytoplasmic     1.334
                                        Cytoskeletal    0.007
                                        ER              0.084
                                        Golgi           0.013
```

Table S1. Subcellular localization of proteins retrieved for TriTypDB database showing >80% homology among *L. infantum, L. major* and *L. braziliensis*.

```
                                       Lysosomal              0.080
                                       Mitochondrial  0.286
                                       Chloroplast    0.245
                                       Peroxisomal    0.072
                                       Vacuole        0.224
                                       Nuclear        0.317


**********************************************************************
************
SeqID: li|LinJ_21_0440_mRNA|
        CELLO prediction:
                        (predictor     location       reliable-index)
                        Composition    Nuclear        0.711
                        Di-peptide     Chloroplast    0.591
                        part-Comp.     PlasmaMembrane 0.265
                        chemo-typy     Nuclear        0.443
                        Neighboring    Nuclear        0.633

        Combined SVM classifier:
                                       Extracellular  0.191
                                       PlasmaMembrane 0.499
                                       Cytoplasmic    0.344
                                       Cytoskeletal   0.237
                                       ER             0.017
                                       Golgi          0.030
                                       Lysosomal              0.020
                                       Mitochondrial  0.596
                                       Chloroplast    0.920
                                       Peroxisomal    0.073
                                       Vacuole        0.015
                                       Nuclear        2.058 *


**********************************************************************
************
SeqID: li|LinJ_36_2100_mRNA|
        CELLO prediction:
                        (predictor     location       reliable-index)
                        Composition    Extracellular  0.503
                        Di-peptide     Extracellular  0.643
                        part-Comp.     Nuclear        0.473
                        chemo-typy     Chloroplast    0.360
                        Neighboring    Extracellular  0.690

        Combined SVM classifier:
                                       Extracellular  1.921 *
                                       PlasmaMembrane 0.059
                                       Cytoplasmic    0.811
                                       Cytoskeletal   0.019
                                       ER             0.018
                                       Golgi          0.057
                                       Lysosomal              0.016
                                       Mitochondrial  0.337
                                       Chloroplast    0.462
                                       Peroxisomal    0.036
                                       Vacuole        0.015
                                       Nuclear        1.250 *
```

Table S1. Subcellular localization of proteins retrieved for TriTypDB database showing >80% homology among *L. infantum, L. major* and *L. braziliensis*.

```
**********************************************************************
************
SeqID: li|LinJ_35_5230_mRNA|
        CELLO prediction:
                        (predictor      location        reliable-index)
                        Composition     Extracellular   0.488
                        Di-peptide      Nuclear         0.470
                        part-Comp.      Cytoplasmic     0.575
                        chemo-typy      Nuclear         0.483
                        Neighboring     Nuclear         0.299

        Combined SVM classifier:
                                        Extracellular   0.663
                                        PlasmaMembrane  0.057
                                        Cytoplasmic     1.404 *
                                        Cytoskeletal    0.041
                                        ER              0.034
                                        Golgi           0.037
                                        Lysosomal               0.009
                                        Mitochondrial   0.825
                                        Chloroplast     0.257
                                        Peroxisomal     0.033
                                        Vacuole         0.031
                                        Nuclear         1.610 *


**********************************************************************
************
SeqID: li|LinJ_24_1870_mRNA|
        CELLO prediction:
                        (predictor      location        reliable-index)
                        Composition     Cytoplasmic     0.321
                        Di-peptide      Extracellular   0.424
                        part-Comp.      Cytoplasmic     0.476
                        chemo-typy      Mitochondrial   0.346
                        Neighboring     Nuclear         0.377

        Combined SVM classifier:
                                        Extracellular   0.939
                                        PlasmaMembrane  0.252
                                        Cytoplasmic     1.178 *
                                        Cytoskeletal    0.024
                                        ER              0.032
                                        Golgi           0.055
                                        Lysosomal               0.026
                                        Mitochondrial   0.870
                                        Chloroplast     0.566
                                        Peroxisomal     0.109
                                        Vacuole         0.021
                                        Nuclear         0.927


**********************************************************************
************
SeqID: li|LinJ_36_0510_mRNA|
        CELLO prediction:
                        (predictor      location        reliable-index)
                        Composition     Cytoplasmic     0.354
                        Di-peptide      Cytoplasmic     0.590
                        part-Comp.      Mitochondrial   0.277
                        chemo-typy      Mitochondrial   0.280
```

Table S1. Subcellular localization of proteins retrieved for TriTypDB database showing >80% homology among *L. infantum, L. major* and *L. braziliensis*.

```
                   Neighboring    Nuclear        0.334

       Combined SVM classifier:
                                  Extracellular  0.646
                                  PlasmaMembrane 0.074
                                  Cytoplasmic    1.499 *
                                  Cytoskeletal   0.022
                                  ER             0.148
                                  Golgi          0.029
                                  Lysosomal             0.163
                                  Mitochondrial  0.983
                                  Chloroplast    0.376
                                  Peroxisomal    0.121
                                  Vacuole        0.066
                                  Nuclear        0.873


*******************************************************************
************
SeqID: li|LinJ_34_0670_mRNA|
       CELLO prediction:
                   (predictor     location       reliable-index)
                   Composition    Mitochondrial  0.460
                   Di-peptide     Chloroplast    0.383
                   part-Comp.     Nuclear        0.546
                   chemo-typy     Cytoplasmic    0.569
                   Neighboring    Mitochondrial  0.600

       Combined SVM classifier:
                                  Extracellular  0.136
                                  PlasmaMembrane 0.058
                                  Cytoplasmic    1.235 *
                                  Cytoskeletal   0.014
                                  ER             0.026
                                  Golgi          0.019
                                  Lysosomal             0.018
                                  Mitochondrial  1.642 *
                                  Chloroplast    0.903
                                  Peroxisomal    0.112
                                  Vacuole        0.015
                                  Nuclear        0.822


*******************************************************************
************
SeqID: li|LinJ_13_0460_mRNA|
       CELLO prediction:
                   (predictor     location       reliable-index)
                   Composition    Chloroplast    0.255
                   Di-peptide     Chloroplast    0.402
                   part-Comp.     Cytoplasmic    0.711
                   chemo-typy     Cytoplasmic    0.871
                   Neighboring    Cytoplasmic    0.459

       Combined SVM classifier:
                                  Extracellular  0.460
                                  PlasmaMembrane 0.072
                                  Cytoplasmic    2.619 *
                                  Cytoskeletal   0.019
                                  ER             0.044
                                  Golgi          0.052
```

Table S1. Subcellular localization of proteins retrieved for TriTypDB database showing >80% homology among *L. infantum, L. major* and *L. braziliensis*.

```
                                        Lysosomal               0.013
                                        Mitochondrial   0.318
                                        Chloroplast     0.905
                                        Peroxisomal     0.089
                                        Vacuole         0.044
                                        Nuclear         0.366


**********************************************************************
************
SeqID: li|LinJ_13_1080_mRNA|
        CELLO prediction:
                        (predictor      location        reliable-index)
                        Composition     Cytoplasmic     0.290
                        Di-peptide      Mitochondrial   0.370
                        part-Comp.      Cytoplasmic     0.454
                        chemo-typy      Cytoplasmic     0.597
                        Neighboring     Nuclear         0.247

        Combined SVM classifier:
                                        Extracellular   0.354
                                        PlasmaMembrane  0.405
                                        Cytoplasmic     1.756 *
                                        Cytoskeletal    0.020
                                        ER              0.130
                                        Golgi           0.273
                                        Lysosomal               0.035
                                        Mitochondrial   0.506
                                        Chloroplast     0.264
                                        Peroxisomal     0.064
                                        Vacuole         0.107
                                        Nuclear         1.085 *


**********************************************************************
************
SeqID: li|LinJ_22_0490_mRNA|
        CELLO prediction:
                        (predictor      location        reliable-index)
                        Composition     Cytoplasmic     0.532
                        Di-peptide      Cytoplasmic     0.733
                        part-Comp.      Cytoplasmic     0.540
                        chemo-typy      Cytoplasmic     0.928
                        Neighboring     Cytoplasmic     0.752

        Combined SVM classifier:
                                        Extracellular   0.023
                                        PlasmaMembrane  0.009
                                        Cytoplasmic     3.485 *
                                        Cytoskeletal    0.024
                                        ER              0.040
                                        Golgi           0.035
                                        Lysosomal               0.002
                                        Mitochondrial   0.653
                                        Chloroplast     0.182
                                        Peroxisomal     0.036
                                        Vacuole         0.014
                                        Nuclear         0.496
```

Table S1. Subcellular localization of proteins retrieved for TriTypDB database showing >80% homology among *L. infantum, L. major* and *L. braziliensis.*

```
**********************************************************************
************
SeqID: li|LinJ_34_3440_mRNA|
        CELLO prediction:
                        (predictor      location        reliable-index)
                        Composition     Cytoplasmic     0.707
                        Di-peptide      Mitochondrial   0.687
                        part-Comp.      Mitochondrial   0.445
                        chemo-typy      Nuclear         0.553
                        Neighboring     Cytoplasmic     0.408

        Combined SVM classifier:
                                        Extracellular   0.033
                                        PlasmaMembrane  0.026
                                        Cytoplasmic     1.484 *
                                        Cytoskeletal    0.013
                                        ER              0.012
                                        Golgi           0.012
                                        Lysosomal               0.009
                                        Mitochondrial   2.116 *
                                        Chloroplast     0.190
                                        Peroxisomal     0.115
                                        Vacuole         0.015
                                        Nuclear         0.975


**********************************************************************
************
SeqID: li|LinJ_31_0930_mRNA|
        CELLO prediction:
                        (predictor      location        reliable-index)
                        Composition     Mitochondrial   0.244
                        Di-peptide      Cytoplasmic     0.308
                        part-Comp.      Chloroplast     0.500
                        chemo-typy      Extracellular   0.479
                        Neighboring     Extracellular   0.256

        Combined SVM classifier:
                                        Extracellular   1.288 *
                                        PlasmaMembrane  0.714
                                        Cytoplasmic     0.711
                                        Cytoskeletal    0.014
                                        ER              0.066
                                        Golgi           0.022
                                        Lysosomal               0.022
                                        Mitochondrial   0.645
                                        Chloroplast     1.096 *
                                        Peroxisomal     0.044
                                        Vacuole         0.034
                                        Nuclear         0.344


**********************************************************************
************
SeqID: li|LinJ_27_1450_mRNA|
        CELLO prediction:
                        (predictor      location        reliable-index)
                        Composition     Nuclear         0.368
                        Di-peptide      Nuclear         0.778
                        part-Comp.      Nuclear         0.729
                        chemo-typy      Cytoplasmic     0.674
```

Table S1. Subcellular localization of proteins retrieved for TriTypDB database showing >80% homology among *L. infantum, L. major* and *L. braziliensis*.

```
                       Neighboring     Nuclear         0.516

        Combined SVM classifier:
                                       Extracellular   0.107
                                       PlasmaMembrane  0.016
                                       Cytoplasmic     1.238
                                       Cytoskeletal    0.082
                                       ER              0.012
                                       Golgi           0.153
                                       Lysosomal               0.007
                                       Mitochondrial   0.784
                                       Chloroplast     0.086
                                       Peroxisomal     0.022
                                       Vacuole         0.007
                                       Nuclear         2.484 *


**********************************************************************
************
SeqID: li|LinJ_21_0800_mRNA|
        CELLO prediction:
                       (predictor      location        reliable-index)
                       Composition     Cytoplasmic     0.742
                       Di-peptide      Nuclear         0.588
                       part-Comp.      Nuclear         0.464
                       chemo-typy      Nuclear         0.901
                       Neighboring     Nuclear         0.392

        Combined SVM classifier:
                                       Extracellular   0.072
                                       PlasmaMembrane  0.023
                                       Cytoplasmic     1.346
                                       Cytoskeletal    0.018
                                       ER              0.015
                                       Golgi           0.008
                                       Lysosomal               0.008
                                       Mitochondrial   0.907
                                       Chloroplast     0.103
                                       Peroxisomal     0.024
                                       Vacuole         0.011
                                       Nuclear         2.464 *


**********************************************************************
************
SeqID: li|LinJ_35_0240_mRNA|
        CELLO prediction:
                       (predictor      location        reliable-index)
                       Composition     Extracellular   0.659
                       Di-peptide      Nuclear         0.297
                       part-Comp.      Mitochondrial   0.315
                       chemo-typy      Nuclear         0.694
                       Neighboring     Nuclear         0.466

        Combined SVM classifier:
                                       Extracellular   1.447 *
                                       PlasmaMembrane  0.049
                                       Cytoplasmic     0.428
                                       Cytoskeletal    0.009
                                       ER              0.013
                                       Golgi           0.014
```

Table S1. Subcellular localization of proteins retrieved for TriTypDB database showing >80% homology among *L. infantum, L. major* and *L. braziliensis*.

```
                                        Lysosomal             0.016
                                        Mitochondrial  0.902
                                        Chloroplast    0.261
                                        Peroxisomal    0.050
                                        Vacuole        0.009
                                        Nuclear        1.802 *


************************************************************************************
SeqID: li|LinJ_10_0830_mRNA|
        CELLO prediction:
                        (predictor     location       reliable-index)
                        Composition    Extracellular  0.449
                        Di-peptide     Extracellular  0.206
                        part-Comp.     Extracellular  0.912
                        chemo-typy     Nuclear        0.462
                        Neighboring    Cytoplasmic    0.390

        Combined SVM classifier:
                                        Extracellular  2.017 *
                                        PlasmaMembrane 0.584
                                        Cytoplasmic    0.833
                                        Cytoskeletal   0.014
                                        ER             0.161
                                        Golgi          0.066
                                        Lysosomal             0.102
                                        Mitochondrial  0.185
                                        Chloroplast    0.139
                                        Peroxisomal    0.099
                                        Vacuole        0.104
                                        Nuclear        0.695


************************************************************************************
SeqID: li|LinJ_36_4070_mRNA|
        CELLO prediction:
                        (predictor     location       reliable-index)
                        Composition    PlasmaMembrane 0.359
                        Di-peptide     Extracellular  0.593
                        part-Comp.     Extracellular  0.609
                        chemo-typy     Chloroplast    0.230
                        Neighboring    Extracellular  0.606

        Combined SVM classifier:
                                        Extracellular  2.224 *
                                        PlasmaMembrane 0.546
                                        Cytoplasmic    0.484
                                        Cytoskeletal   0.010
                                        ER             0.101
                                        Golgi          0.041
                                        Lysosomal             0.251
                                        Mitochondrial  0.421
                                        Chloroplast    0.377
                                        Peroxisomal    0.125
                                        Vacuole        0.131
                                        Nuclear        0.287
```

Table S1. Subcellular localization of proteins retrieved for TriTypDB database showing >80% homology among *L. infantum*, *L. major* and *L. braziliensis*.

```
*********************************************************************
************
SeqID: li|LinJ_35_1310_mRNA|
       CELLO prediction:
                       (predictor     location       reliable-index)
                       Composition    Mitochondrial  0.359
                       Di-peptide     Nuclear        0.288
                       part-Comp.     Nuclear        0.425
                       chemo-typy     Nuclear        0.323
                       Neighboring    Mitochondrial  0.342

       Combined SVM classifier:
                                      Extracellular  0.541
                                      PlasmaMembrane 0.112
                                      Cytoplasmic    0.899
                                      Cytoskeletal   0.022
                                      ER             0.029
                                      Golgi          0.019
                                      Lysosomal              0.061
                                      Mitochondrial  1.263 *
                                      Chloroplast    0.484
                                      Peroxisomal    0.120
                                      Vacuole        0.059
                                      Nuclear        1.391 *

*********************************************************************
************
SeqID: li|LinJ_34_2720_mRNA|
       CELLO prediction:
                       (predictor     location       reliable-index)
                       Composition    Cytoplasmic    0.804
                       Di-peptide     Mitochondrial  0.867
                       part-Comp.     Mitochondrial  0.806
                       chemo-typy     Mitochondrial  0.635
                       Neighboring    Mitochondrial  0.567

       Combined SVM classifier:
                                      Extracellular  0.043
                                      PlasmaMembrane 0.023
                                      Cytoplasmic    1.373
                                      Cytoskeletal   0.012
                                      ER             0.013
                                      Golgi          0.007
                                      Lysosomal              0.005
                                      Mitochondrial  2.979 *
                                      Chloroplast    0.175
                                      Peroxisomal    0.039
                                      Vacuole        0.004
                                      Nuclear        0.325

*********************************************************************
************
```
