# Supplementary material for: Immunoinformatics Approach to Design a Multi-Epitope Nanovaccine against Leishmania Parasite: Elicitation of Cellular Immune Responses
Source: Vaccines (Basel). 2023 Jan 30;11(2):304. doi: 10.3390/vaccines11020304 (PMC9960668; doi:10.3390/vaccines11020304)
Supplement: Supplementary file 1 [file vaccines-11-00304-s001.zip › Table S2.pdf]

Table S2. MHCI and MHCII epitopes selected as candidates for vaccine construct

No	Protein (Code)	MHCI Epitopes	MHCII Epitopes
1-3	LinJ.08.1290/ LinJ.08.1280/ LinJ.21.2240	60-VPRAVLMDL-68	57-GRYVPRAVLMDLEPG-71
		78-AGPYGQLFR-86	160-PDRIMMTFSVIPSPR-174
		105-HYTEGAELI-113	161-DRIMMTFSVIPSPRV-175
		143-TGSGMGTLL-151	162-RIMMTFSVIPSPRVS-176
		159-YPDRIMMTF-167	163-IMMTFSVIPSPRVSD-177
		242-FPGQLNSDL-250	221-TFGDLNHLVAAVMSG-235
		258-VPFPRLHFF-266	305-PRHGRYLTASALFRG-319
		259-PFPRLHFFM-267	306-RHGRYLTASALFRGR-320
		260-FPRLHFFMM-268	307-HGRYLTASALFRGRM-321
		345-IPNNIKSSI-353	308-GRYLTASALFRGRMS-322
		366-TFIGNNTCI-374	309-RYLTASALFRGRMST-323
		381-VGEQFTGMF-389	382-GEQFTGMFRRKAFLH-396
		386-TGMFRRKAF-394	
4	LinJ.36.5660	21-SYVPSEALM-29	30-HEDGQFLRAFLLRVL-44
		169-NPNAAAMAL-177	31-EDGQFLRAFLLRVLA-45
			32-DGQFLRAFLLRVLAN-46
			33-GQFLRAFLLRVLAND-47
			34-QFLRAFLLRVLANDM-48
			35-FLRAFLLRVLANDMQ-49
			37-RAFLLRVLANDMQRM-51
			46-NDMQRMLATNTCAAY-60
			96-SKLLRTYVERAEYAA-110
			209-AVEFRENNRRRMAQV-223
5	LinJ.35.1030	103-RRLSLKTTL-111	94-LEDLFSFCGRRLSLK-108
		170-IPYKEGKSL-178	95-EDLFSFCGRRLSLKT-109
		18-GSGSFGEIF-26	96-DLFSFCGRRLSLKTT-110
		52-TYESRFYRI-60	121-IEFVHSKSVLHRDIK-135
		90-LGPSLEDLF-98	254-AAYMNYVRALRFEDK-268
		122-EFVHSKSVL-130	255-AYMNYVRALRFEDKP-269
		160-KYRDPRTHA-168	
		162-RDPRTHAHI-170	
		179-TGTARYCSI-187	
		183-RYCSINTHM-191	
		255-AYMNYVRAL-269	
6	LinJ.28.0210	128-KYNASRGEA-136	41-RSQHWDLYIHRALRR-55
			42-SQHWDLYIHRALRRF-56
			43-QHWDLYIHRALRRFF-57
			44-HWDLYIHRALRRFFK-58
			45-WDLYIHRALRRFFKR-59
			46-DLYIHRALRRFFKRG-60
			51-RALRRFFKRGTLSKA-65
			53-LRRFFKRGTLSKAAV-67
			56-FFKRGTLSKAAVRVL-70

Table S2. MHCI and MHCII epitopes selected as candidates for vaccine construct

			61-TLSKAAVRVLSSFIE-75
			63-SKAAVRVLSSFIEDM-77
			75-EDMFNRIQTEAVFVA-89
			76-DMFNRIQTEAVFVAK-90
			77-MFNRIQTEAVFVAKI-91
			81-IQTEAVFVAKINKVK-95
			83-TEAVFVAKINKVKTL-97
			84-EAVFVAKINKVKTLT-98
			85-AVFVAKINKVKTLTA-99
			86-VFVAKINKVKTLTAR-100
			88-VAKINKVKTLTAREI-102
			89-AKINKVKTLTAREIQ-103
			91-INKVKTLTAREIQTS-115
7	LinJ.34.3620	58-VPLSDQVKL-66	1-MLRFFRARLAPTTTD-15
		177-KYHLKAVVL-185	2-LRFFRARLAPTTTDA-16
			26-VNKTWFRHNLIIRRK-40
			74-CKHVRLIAKATAERF-88
			75-KHVRLIAKATAERFA-89
			76-HVRLIAKATAERFAH-90
			87-RFAHCRVFPAVAHRV-101
			88-FAHCRVFPAVAHRVS-102
			90-HCRVFPAVAHRVSVQ-114
			91-CRVFPAVAHRVSVQR-115
			92-RVFPAVAHRVSVQRF-116
			176-HKYHLKAVVLANFFV-190
8	LinJ.35.1540	244-VPIPNEGLF-252	1-MFRRSFISAFQATRA-15
		246-IPNEGLFNG-252	2-FRRSFISAFQATRAA-16
		281-VPPYRWIDD-289	3-RRSFISAFQATRAAR-17
			4-RSFISAFQATRAARV-18
			5-SFISAFQATRAARVS-19
			6-FISAFQATRAARVSL-20
			7-ISAFQATRAARVSLV-21
			8-SAFQATRAARVSLVF-22
			9-AFQATRAARVSLVFK-23
			10-FQATRAARVSLVFKQ-24
			111-EFFRKHILKPKLADD-125
			141-MLGFVILMARYAVLP-155
			142-LGFVILMARYAVLPL-156
			143-GFVILMARYAVLPLW-157
			146-ILMARYAVLPLWYVG-160
			163-AMSMVGQMNIEAEIG-177
			186-TVVWRGKPVFVYRRS-190
			191-GKPVFVYRRSARQMK-105
			201-ARQMKEVMETPLSAL-215
9	LinJ.21.0440	5-SYQAGFLSI-13	74-PFLVMIIKNMKKYFS-90
		9-GFLSILYSI-17	146-TNYVETLRVQVHANC-160

Table S2. MHCI and MHCII epitopes selected as candidates for vaccine construct

		14-LYSIGSKPL-22	161-RIRRLYFSDRLYAEE-175
		81-KNMKKYFSF-89	
		85-KYFSFEVTI-99	
		177-LPPEFKLFL-191	
10	LinJ.36.6680	37-NYLKRVRGI-45	36-ANYLKRVRGIKAKLA-50
		81-GPKNALPGF-89	37-NYLKRVRGIKAKLAQ-51
		184-THPELKATF-192	38-YLKRVRGIKAKLAQK-52
		230-VTPSGKVVW-238	39-LKRVRGIKAKLAQKA-53
		238-WGKYAQVTN-246	41-RVRGIKAKLAQKARY-55
		240-KYAQVTNNP-248	165-PKMERFIRPMALRFK-179
			166-KMERFIRPMALRFKK-180
			167-MERFIRPMALRFKKA-181
			168-ERFIRPMALRFKKAH-182
			169-RFIRPMALRFKKAHV-183
			205-GKMYTGLGVITKGSV-219
11	LinJ.16.0470	6-GYKSGTRHL-14	106-KAKEHQFQAYLAAKK-120
		22-HGAPSVSTI-30	140-PKNVEVLARRVADYE-154
		55-KYYHGRTGI-63	
		67-VTPRGVGVI-75	
		114-AYLAAKKAG-122	
12	LinJ.32.0930	3-KPHLRHYQV-11	36-FVVAKSRFWRMMRVK-50
		21-NPEPTVYKF-29	41-SRFWRMMRVKNKVKA-55
		131-IPDHEVKHL-139	117-RARYHNIEVLNVKSI-131
		145-HAPNLSFPL-153	136-VKHLSIAQYHAPNLS-150
		146-APNLSFPLL-154	148-NLSFPLLQRRIKAAR-162
13	LinJ.24.0040	39-MPLRKAQQL-47	45-QQLYRQVLAKTRCIP-59
		87-SVVAMMSLL-95	82-RWPRKSVVAMMSLLK-96
		138-PYMCSPCHV-146	83-WPRKSVVAMMSLLKN-97
		142-SPCHVQLFM-150	84-PRKSVVAMMSLLKNA-98
			85-RKSVVAMMSLLKNAE-99
			86-KSVVAMMSLLKNAEA-100
			89-VAMMSLLKNAEANAI-103
			92-MSLLKNAEANAIEKG-106
			111-QMVIKHVQVDQAARM125
14	LinJ.22.1300	51-VVPRTSENF-59	1-MWCLSRVALQSNRAA-15
		108-GYPFPDESF-116	2-WCLSRVALQSNRAAL-16
		134-GPNQNGSQF-142	3-CLSRVALQSNRAALY-17
		136-NQNGSQFFF-142	4-LSRVALQSNRAALYM-18
		155-KFVVVGQVL-163	5-SRVALQSNRAALYMP-19
		216-MPGKEVLDL-222	6-RVALQSNRAALYMPY-20
			7-VALQSNRAALYMPYT-21
			13-RAALYMPYTPVAANP-27
15	LinJ.27.1450		61-ESPFVQIAGYRVPHP-75
16	LinJ.28.2200	4-FHPRDAFIL-12	61-NPLRHVLMQNAAVTS-75
		80-IPHPLEPKM-88	66-VLMQNAAVTSAGYAI-70
		82-HPLEPKMLL-90	86-PKMLLHVQASDYAVE-100

Table S2. MHCI and MHCII epitopes selected as candidates for vaccine construct

			121-DNCMVEQHRMAAATQ-135
17	LinJ.34.0420	109-QNAAAYFPL-117	19-SQALREIAANIYNHG-33
		113-AYFPLDTFT-121	58-AITYARYVTLQLDMG-72
		115-FPLDTFTRL-123	
		119-TFTRLEEEI-127	
18	LinJ.28.2940	235-ESPINQIAF-243	246-NRFWMCVATERSLSV-260
		19-CPQQAGSYI-27	247-RFWMCVATERSLSVY-261
		25-SYIKVVSTS-33	249-WMCVATERSLSVYDL-263
		195-NYVSTVTVS-203	
		230-FKINVESPI-238	
19	LinJ.10.0050	109-RPHGNAGMM-117	90-MRAVGGVRTKSPGPG-113
20	LinJ.33.1560	102-SPFGYEHAM-110	
21	LinJ.18.0650	81-HYYLVLELV-89	51-EDQMLREVAIMRSLR-65
		121-YYCHSKGFA-129	52-DQMLREVAIMRSLRQ-66
		181-GYNGLSADI-189	53-QMLREVAIMRSLRQQ-67
		196-LYVMLAGRL-204	54-MLREVAIMRSLRQQN-68
			55-LREVAIMRSLRQQNV-69
			56-REVAIMRSLRQQNVV-70
			57-EVAIMRSLRQQNVVK-71
			58-VAIMRSLRQQNVVKL-72
			195-VLYVMLAGRLPFEDR-209
22	LinJ.20.0250	71-YYWAILCAV-79	69-REYYWAILCAVLGYL-83
		81-GYLAAHALV-89	72-YWAILCAVLGYLAAH-86
		134-VYSKRKAAV-142	76-LCAVLGYLAAHALVF-90
			77-CAVLGYLAAHALVFN-91
			78-AVLGYLAAHALVFNY-92
			79-VLGYLAAHALVFNYY-93
			121-AVDEMVGLSVLESVY-135
			122-VDEMVGLSVLESVYS-136
			125-MVGLSVLESVYSKRK-139
23	LinJ.35.3810	70-KPTINLNNL-78	76-NNLSRLIAAEEAAKA-90
			78-LSRLIAAEEAAKATK-92
			114-HIQVPCIVKARYVSK-128
24	LinJ.20.0600	56-SPITCNFEY-64	16-RANAFFSRLPIARIQ-30
			17-ANAFFSRLPIARIQR-31
			18-NAFFSRLPIARIQRA-32
			19-AFFSRLPIARIQRAL-33
			20-FFSRLPIARIQRALA-34
			21-FSRLPIARIQRALAM-35
			22-SRLPIARIQRALAME-36
			23-RLPIARIQRALAMEA-37
			24-LPIARIQRALAMEAI-38
			25-PIARIQRALAMEAIK-39
			26-IARIQRALAMEAIKK-40
			27-ARIQRALAMEAIKKG-41
			32-ALAMEAIKKGSMKPW-46

Table S2. MHCI and MHCII epitopes selected as candidates for vaccine construct

			66-PRPVRLIGTVMDAHT-80
25	LinJ.28.0780	34-TNPYNRMFW-42	3-QCTSLLCRKALQAYP-17
		100-KPQRESRHL-108	4-CTSLLCRKALQAYPV-18
		111-MPMTPRVVF-119	5-TSLLCRKALQAYPVP-19
			6-SLLCRKALQAYPVPP-20
			7-LLCRKALQAYPVPPR-21
			8-LCRKALQAYPVPPRA-22
			9-CRKALQAYPVPPRAR-23
			10-RKALQAYPVPPRARN-24
			19-PPRARNYERRWSSSR-33
			20-PRARNYERRWSSSRT-34
			21-RARNYERRWSSSRTN-35
			22-ARNYERRWSSSRTNP-36
			33-RTNPYNRMFWRTVLN-47
			34-TNPYNRMFWRTVLNE-48
			35-NPYNRMFWRTVLNED-49
			49-DFARPSFWVSDFRHK-63
			50-FARPSFWVSDFRHKY-64
			51-ARPSFWVSDFRHKYL-65
			52-RPSFWVSDFRHKYLA-66
			53-PSFWVSDFRHKYLAK-67
			54-SFWVSDFRHKYLAKH-68
			55-FWVSDFRHKYLAKHG-69
			57-VSDFRHKYLAKHGMD-71
			60-FRHKYLAKHGMDYQG-74
			69-GMDYQGRVPASPAPG-83
			70-MDYQGRVPASPAPGM-84
			71-DYQGRVPASPAPGMY-85
			101-PQRESRHLPVMPMTP-115
			102-QRESRHLPVMPMTPR-116
			103-RESRHLPVMPMTPRV-117
			104-ESRHLPVMPMTPRVV-118
			105-SRHLPVMPMTPRVVF-119
			106-RHLPVMPMTPRVVFE-120
			115-PRVVFEHAQEKRIDY-129
			124-EKRIDYAKKMHRDRR-138
			125-KRIDYAKKMHRDRRL-139
			126-RIDYAKKMHRDRRLV-140
			127-IDYAKKMHRDRRLVE-141
			128-DYAKKMHRDRRLVEQ-142
			129-YAKKMHRDRRLVEQL-143
			134-HRDRRLVEQLRTHEF-148
			135-RDRRLVEQLRTHEFW-149
			145-THEFWGWYMKLQRVR-159
			146-HEFWGWYMKLQRVRG-160
			147-EFWGWYMKLQRVRGR-161

Table S2. MHCI and MHCII epitopes selected as candidates for vaccine construct

			148-FWGWYMKLQRVRGRW-162
			149-WGWYMKLQRVRGRWC-163
			150-GWYMKLQRVRGRWCK-164
			151-WYMKLQRVRGRWCKE-165
			152-YMKLQRVRGRWCKEH-166
26	LinJ.33.3340	57-IAFDKHFNL-65	46-CRNSKALLAHVIAFD-60
		89-LFLRGESVI-97	
