# Supplementary material for: Immunoinformatics Approach to Design a Multi-Epitope Nanovaccine against Leishmania Parasite: Elicitation of Cellular Immune Responses
Source: Vaccines (Basel). 2023 Jan 30;11(2):304. doi: 10.3390/vaccines11020304 (PMC9960668; doi:10.3390/vaccines11020304)
Supplement: Supplementary file 1 [file vaccines-11-00304-s001.zip › Table S3.pdf]

Table S3. Linear and conformational B cell epitopes predicted for LeishChim with ElliPro server

Protein	A/A	Linear epitopes	Score	Conformational epitopes	Score
LeishChim	1	109-RDADNTAITYARYVT-123	0.788	A:E405, A:D406, A:R407	0.995
	2	347-IVAAKRFDEPTARRYFHQLIAGMYYCHSKGFAGYNGLSADIWSCGVVLYVMLAGRLPFEDR-407	0.787	A:S106, A:D110, A:A111, A:D112, A:N113, A:T114, A:A115, A:I116, A:T117	0.78
	3	181-EEEIRANAF-189	0.745	A:A308, A:I309, A:R311, A:S312, A:L313, A:Q315, A:N317, A:F344, A:I347, A:V348, A:A349, A:A350, A:K351, A:R352, A:F353, A:D354, A:E355, A:P356, A:T357, A:A358, A:R359, A:R360, A:F362, A:H363, A:L365, A:I366, A:A367, A:G368, A:M369, A:Y370, A:Y371, A:C372, A:H373, A:S374, A:K375, A:G376, A:F377, A:A378, A:G379, A:Y380, A:N381, A:G382, A:L383, A:S384, A:A385, A:D386, A:I387, A:W388, A:S389, A:C390, A:G391, A:V392, A:V393, A:L394, A:Y395, A:V396, A:M397, A:L398, A:A399, A:G400, A:R401, A:L402, A:P403, A:F404	0.755
	4	292-PMTPRVVFEDQMLREVAIMRSL-313	0.715	A:P269, A:S270, A:F271, A:W272, A:P292, A:M293, A:T294, A:P295, A:R296, A:V297, A:V298, A:F299, A:E300, A:D301, A:Q302, A:M303, A:L304, A:R305, A:E306	0.743
	5	51-EICDDTISSFDNCMVEQH-68	0.692	A:H217, A:T218, A:K219, A:E220, A:Q221, A:G224, A:S225, A:P226, A:I227, A:T228, A:C229, A:N230, A:F231, A:E232, A:Y233, A:N234, A:P235, A:R236, A:P237, A:V238, A:R239	0.66
	6	224-GSPITCNFEYNPRPV-238	0.668	A:N1, A:P2, A:L3, A:R4, A:H5, A:V6, A:Q9, A:N10, A:A11, A:A12, A:V13, A:K27, A:V32, A:Q33, A:A34, A:S35, A:D36, A:Y37, A:A38, A:V39, A:E40, A:A41, A:V42, A:A43, A:K44, A:G45, A:E47, A:R48, A:E51, A:I52, A:C53, A:D54, A:D55, A:T56, A:I57, A:S58, A:S59, A:F60, A:D61, A:N62, A:C63, A:M64, A:V65, A:E66, A:Q67, A:H68, A:A71, A:A72, A:A73, A:T74, A:Q75, A:S76, A:Q77, A:A78, A:L79, A:R80, A:E81, A:I86, A:F177, A:E181, A:E182, A:E183, A:R185, A:A186, A:N187, A:A188, A:F189	0.632
	7	71-AAATQSQALRE-81	0.666	A:T249, A:T250, A:N251, A:P252, A:Y253, A:N254, A:R255, A:M256, A:L325, A:Q326, A:S327, A:S328, A:N329, A:H330	0.601
	8	325-LQSSNH-330	0.659	A:M212, A:K213, A:P214, A:W215	0.587
	9	32-VQASDYAVEAVAKGLER-48	0.625	A:T243, A:M245, A:D246, A:H248	0.586
	10	212-MKPWKHTKEQ-221	0.615	A:Y118, A:A119, A:R120, A:Y121, A:V122, A:T123, A:L124, A:E142, A:A144, A:D145, A:V146, A:N147, A:I148, A:T149, A:L150, A:R151, A:N153, A:N154, A:L155, A:R157, A:P158, A:T159, A:G160, A:V161, A:K162, A:H163, A:N164, A:Q165, A:Q166, A:N167, A:A168, A:A169, A:A170, A:F257	0.552
	11	157-RPTGVKHNQQN-167	0.599	A:A83, A:Y87, A:N88, A:H89, A:G90, A:G91, A:I93	0.541
	12	245-MDAHTTNPYNR-255	0.599		

	13	1-NPLRHV-6	0.563
	14	86-IYNHGG-91	0.557
