# Supplementary material for: Immunoinformatics Approach to Design a Multi-Epitope Nanovaccine against Leishmania Parasite: Elicitation of Cellular Immune Responses
Source: Vaccines (Basel). 2023 Jan 30;11(2):304. doi: 10.3390/vaccines11020304 (PMC9960668; doi:10.3390/vaccines11020304)
Supplement: Supplementary file 1 [file vaccines-11-00304-s001.zip › Table S4.pdf]

Table S4. CTL and HTL epitopes predicted for LeishChim with NetCTL and IEDB servers, respectively. Epitopes originally retrieved from *Leishmania* proteins are shown in blue font

	CTL		HTL	
No	Residue No	Epitope	Residue No	Epitope
1	8	MQNAAVTSA	1	NPLRHVLMQNAAVTS
2	10	NAAVTSAGY	116	ITYARYVTLQLDMGE
3	12	AVTSAGYAI	166	QNAAAYFPLDTFTRL
4	19	AIPHPLEPK	186	ANAFFSRLPIARIQR
5	22	HPLEPKMLL	191	SRLPIARIQRALAME
6	24	LEPKMLLHV	196	ARIQRALAMEAIKKG
7	29	LLHVQASDY	201	ALAMEAIKKGSMKPW
8	34	ASDYAVEAV	251	NPYNRMFWRTVLNED
9	41	AVAKGLERL	301	DQMLREVAIMRSLRQ
10	69	RMAAATQSQ	306	EVAIMRSLRQQNVVK
11	71	AAATQSQAL	356	PTARRYFHQLIAGMY
12	78	ALREIAANI		
13	79	LREIAANIY		
14	81	EIAANIYNH		
15	85	NIYNHGGLI		
16	88	NHGGLIRKL		
17	95	KLSNEGILR		
18	97	SNEGILRPY		
19	101	ILRPYSRFR		
20	103	RPYSRFRDA		
21	108	FRDADNTAI		
22	110	DADNTAITY		
23	113	NTAITYARY		
24	116	ITYARYVTL		
25	117	TYARYVTLQ		
26	118	YARYVTLQL		
27	120	RYVTLQLDM		
28	142	EHADVNITL		
29	144	ADVNITLRL		
30	154	NLERPTGVK		
31	163	HNQQNAAAY		
32	164	NQQNAAAYF		
33	166	QNAAAYFPL		
34	169	AAYFPLDTF		
35	171	YFPLDTFTR		
36	172	FPLDTFTRL		
37	181	EEEIRANAF		
38	182	EEIRANAFF		
39	184	IRANAFFSR		
40	185	RANAFFSRL		
41	185	RANAFFSRL		
42	192	RLPIARIQR		
43	194	PIARIQRAL		
44	196	ARIQRALAM		
45	198	IQRALAMEA		
46	199	QRALAMEAI		

Table S4. CTL and HTL epitopes predicted for LeishChim with NetCTL and IEDB servers, respectively. Epitopes originally retrieved from *Leishmania* proteins are shown in blue font

47	200	RALAMEAIK		
48	201	ALAMEAIKK		
49	211	SMKPWKHTK		
50	213	KPWKHTKEQ		
51	219	KEQIIGSPI		
52	223	IGSPITCNF		
53	225	SPITCNFEY		
54	232	EYNPRPVRL		
55	234	NPRPVRLIG		
56	236	RPVRLIGTV		
57	237	PVRLIGTVM		
58	239	RLIGTVMDA		
59	245	MDAHTTNPY		
60	248	HTTNPYNRM		
61	249	TTNPYNRMF		
62	254	NRMFWRTVL		
63	255	RMFWRTVLN		
64	258	WRTVLNEDF		
65	263	NEDFARPSF		
66	265	DFARPSFWV		
67	268	RPSFWVSDF		
68	280	KPQRESRHL		
69	282	QRESRHLPV		
70	283	RESRHLPVM		
71	285	SRHLPVMPM		
72	288	LPVMPMTPR		
73	291	MPMTPRVVF		
74	297	VVFEDQMLR		
75	299	FEDQMLREV		
76	302	QMLREVAIM		
77	303	MLREVAIMR		
78	305	REVAIMRSL		
79	312	SLRQQNVVK		
80	313	LRQQNVVKL		
81	317	NVVKLQEVL		
82	323	EVLQSSNHY		
83	324	VLQSSNHYY		
84	325	LQSSNHYYL		
85	326	QSSNHYYLV		
86	327	SSNHYYLVL		
87	329	NHYYLVLEL		
88	330	HYYLVLELV		
89	331	YYLVLELVT		
90	335	LELVTGGEL		
91	336	ELVTGGELF		
92	341	GELFDKIVA		
93	342	ELFDKIVAA		
94	351	KRFDEPTAR		
95	357	TARRYFHQ		

Table S4. CTL and HTL epitopes predicted for LeishChim with NetCTL and IEDB servers, respectively. Epitopes originally retrieved from *Leishmania* proteins are shown in blue font

96	359	RRYFHQLIA		
97	361	YFHQLIAGM		
98	363	HQLIAGMYY		
99	369	MYYCHSKGF		
100	379	GYNGLSADI		
101	384	SADIWSCGV		
102	387	IWSCGVVLY		
103	388	WSCGVVLYV		
104	393	VLYVMLAGR		
105	394	LYVMLAGRL		
106	396	VMLAGRLPF		
